# QBP1 Peptide as a Potential Anti‐Amyloidogenic Therapy for Type 2 Diabetes: An In Vitro Study

**DOI:** 10.1002/advs.202508344

**Published:** 2026-04-22

**Authors:** María M. Tejero‐Ojeda, Ada Bernaus Vives, Michał Wojciechowski, Dinh Quoc Huy Pham, Mateusz Chwastyk, Mario Vallejo, Anna Novials, Douglas V. Laurents, Mariano Carrión‐Vázquez

**Affiliations:** ^1^ Instituto Cajal Consejo Superior de Investigaciones Científicas (CSIC) Madrid Spain; ^2^ PhD Program in Neuroscience Universidad Autónoma de Madrid‐Cajal Institute Madrid Spain; ^3^ Institute of Physics Polish Academy of Sciences Warsaw Poland; ^4^ Centro de Investigación Biomédica en Red de Diabetes y Enfermedades Metabólicas Asociadas CIBERDEM Madrid Spain; ^5^ Instituto de Investigaciones Biomédicas Sols‐Morreale CSIC/Universidad Autónoma de Madrid Madrid Spain; ^6^ Institut d'Investigacions Biomèdiques August Pi i Sunyer (IDIBAPS) Barcelona Spain; ^7^ Instituto de Química Física Blas Cabrera CSIC Madrid Spain

**Keywords:** aggregation, amyloidogenic disease, anti‐amyloidogenic peptide, fibrils, human islet amyloid polypeptide (hIAPP), oligomers, pancreatic β‐cells, protein misfolding, QBP1, type 2 diabetes (T2D)

## Abstract

Self‐assembly and aggregation of human islet amyloid polypeptide (hIAPP, or amylin) into β‐sheet‐rich oligomers and fibrils have been implicated in pancreatic β‐cell dysfunction and failure, contributing to the pathogenesis of type 2 diabetes (T2D). Substantial effort has gone into developing inhibitors, particularly short peptides, capable of targeting hIAPP and disrupting its amyloidogenic process, offering potential therapeutic strategies to prevent or slow T2D progression. Here, we demonstrate the effectiveness of the anti‐amyloidogenic peptide QBP1 in halting the conformational conversion of hIAPP into β‐structured aggregates, thereby preventing amyloidogenesis and its associated cytotoxicity. First, we evaluated the anti‐amyloidogenic effects of QBP1 through cell‐free in vitro aggregation experiments, including Thioflavin‐T fluorescence, A11/OC dot blotting, and negative‐stain electron microscopy. Circular dichroism and nuclear magnetic resonance spectroscopy corroborated that QBP1 delays hIAPP β‐sheet formation and oligomerization, respectively, with an efficacy comparable to that of epigallocatechin‐3‐gallate. Second, we performed cell‐based in vitro experiments to investigate cytoprotection in INS‐1E β‐cells by fusing QBP1 to the cell‐penetrating peptide penetratin (*Antp*‐QBP1). Viability assays, immunocytochemistry, and gene expression analysis showed that under amyloidogenic stress *Antp*‐QBP1 preserves β‐cell viability and metabolic homeostasis by preventing the formation of early toxic hIAPP intermediates. Finally, in silico experiments using molecular dynamics simulations revealed stable QBP1–hIAPP interactions mediated by van der Waals forces and π–H contacts involving hydrophobic and aromatic residues (e.g., W, F), supported by favorable non‐polar solvation and structural complementarity. Taken together, these findings identify QBP1 as a promising candidate for strategies aimed at reducing islet amyloid burden and preserving β‐cell integrity in T2D.

## Graphical abstract

The anti‐amyloidogenic peptide QBP1 effectively halts human islet amyloid polypeptide (hIAPP) aggregation, preventing the formation of toxic β‐structured intermediates. Through a combination of biophysical assays, molecular dynamics, and cell‐based studies, QBP1 is shown to preserve β‐cell viability and metabolic homeostasis, positioning it as a promising therapeutic candidate for reducing amyloid burden in type 2 diabetes.

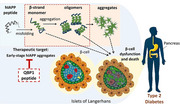

## 1 Introduction

Amyloidogenic diseases represent a diverse group of disorders characterized by misfolding and aggregation of proteins into toxic oligomers and fibrils, which leads to dysfunction and tissue degeneration [1]. In type 2 diabetes (T2D), the aggregation‐prone human islet amyloid polypeptide (hIAPP, or amylin) constitutes the major component of amyloid deposits in pancreatic islets [2, 3]. This 37‐residue peptide is co‐secreted with insulin by pancreatic β‐cells and contributes to postprandial glucose regulation by modulating glucagon secretion and gastric emptying [4, 5]. While the hIAPP monomer is not inherently cytotoxic, its aggregated forms—particularly soluble oligomers—are key contributors to β‐cell dysfunction and death, which ultimately lead to islet failure in T2D [6, 7]. These oligomers disrupt cellular membranes and induce organelle stress [8], effects that are exacerbated by hIAPP hypersecretion, calcium dysregulation, and the activation of proteolytic enzymes [9, 10].

hIAPP has a high propensity for amyloid formation, primarily driven by a central hydrophobic region enriched in β‐sheet–promoting residues that enables formation of the cross‐β structure characteristic of amyloid fibrils [11, 12, 13]. In contrast, rodent IAPP (rIAPP) contains three proline residues (Pro25, Pro28, and Pro29)—absent in the human protein—within this region, which enhance its solubility and prevent its pathological aggregation [4, 14]. A key breakthrough in the development of related therapeutic strategies was the substitution of positions 25, 28, and 29 in hIAPP by proline residues, which was shown to disrupt β‐sheet structure. This modification led to the development of pramlintide (Symlin), an FDA‐approved hIAPP analogue for treating type 1 and type 2 diabetes [15]. In addition, several amyloidogenic “hot spots” have been identified in the hIAPP sequence, particularly in regions spanning residues 8–20, 20–29, and 30–37, which form β‐sheet‐prone domains critical for nucleation, fibril growth, and stabilization of toxic intermediates [16, 17].

Based on this structural knowledge, considerable efforts have been focused on developing inhibitors that prevent hIAPP misfolding and amyloid formation without disrupting its physiological function [18]. Among these, short peptide inhibitors have emerged as a promising strategy, offering high specificity, low toxicity, rapid clearance, efficient permeation, and excellent in vivo biocompatibility [19, 20, 21]. Several sequence‐based peptides effectively block hIAPP aggregation by targeting its critical amyloidogenic regions [22, 23]. Notably, the D‐ANFLVH inhibitor has shown in vivo potential by preventing islet amyloid deposition, reducing β‐cell apoptosis, and improving glucose tolerance in transgenic T2D mouse models [24, 25]. Incorporation of β‐sheet breaker elements such as proline [26] or α‐Aminoisobutyric acid [27, 28] further enhanced their potency. Although pramlintide represents a successful clinical application of this approach, its limitations in solubility, oral bioavailability, and production cost [15, 29] have prompted the search for improved peptide‐based therapeutics, including next‐generation amylinomimetics such as davalintide, cagrilintide and DACRAs [30, 31]. However, these strategies primarily aim to enhance metabolic control rather than directly targeting hIAPP aggregation, highlighting the need for approaches that specifically modulate amyloidogenesis.

Polyglutamine‐binding peptide 1 (**QBP1**; SNWKWWPGIFD), whose minimal active core sequence is the octapeptide QBP1‐M8 (**WKWWPGIF**; hereafter referred to as QBP1), was originally developed as an inhibitor of the early stages of polyglutamine (polyQ) aggregation [32, 33, 34]. QBP1 was shown to interact with polyQ tracts in a random coil conformation, preventing their transition to β‐sheet structures and thereby inhibiting oligomerization, amyloidogenesis, and cytotoxicity in both in vitro and in vivo models [35, 36, 37, 38, 39]. Although its systemic administration was initially hindered by proteolytic degradation and poor blood–brain barrier penetration [34, 40], subsequent findings showed that intranasal delivery significantly improved brain uptake and therapeutic efficacy [39]. Beyond polyQ diseases, QBP1 has also shown an anti‐amyloidogenic effect on other pathological amyloids, including α‐synuclein [35] and TDP‐43 [41], as well as on some functional amyloids such as Sup35 and neuronal CPEBs [42, 43, 44]. Notably, QBP1 does not affect amyloid β 42 (Aβ42) or tau aggregation [35], indicating a degree of selectivity in its mechanism of action.

Nuclear magnetic resonance (NMR) has revealed that, in solution, QBP1 adopts a preferential conformation stabilized by a hydrophobic cluster formed by the indole side chains of its tryptophan (W) residues [45]. A scrambled variant (**SC‐M8**; **WPIWKGWF**), which lacks this structure [38, 45], retains partial activity against some amyloids, suggesting that QBP1's efficacy is not solely dependent on its sequence and conformation but also on its amino acid composition, particularly its high W content. Its mechanism likely involves aromatic‐driven hydrophobic interactions and disruption of amyloid‐stabilizing hydrogen bonds, particularly in glutamine‐ and asparagine‐rich (Q/N‐rich) amyloids such as TDP‐43 [41, 46].

In this study, we investigated whether the inhibitory properties of the anti‐amyloidogenic peptide QBP1 could be extended to hIAPP, with the aim of blocking its critical conformational transition to a β‐structure and thus preventing its amyloidogenesis from the earliest stages. To assess its effect on hIAPP aggregation, we combined biophysical, biochemical, and imaging approaches, complemented by pancreatic β‐cell viability and targeted reverse transcription‐quantitative polymerase chain reaction (RT‐qPCR) gene expression analysis. For efficient intracellular delivery, QBP1 was fused to the penetratin protein transduction domain (PTD) derived from the *Antennapedia* (*Antp*) homeodomain of *Drosophila* [38, 40]. Our results show that QBP1 delays the formation of early hIAPP intermediates, slows amyloid progression, and preserves β‐cell metabolic fitness and viability. Circular dichroism (CD) spectroscopy confirms attenuation of the coil‐to‐β transition, while NMR corroborates inhibition under physiological conditions. Molecular dynamics (MD) simulations reveal a stable interaction network mediated by van der Waals forces and π–H contacts at key hydrophobic and aromatic residues, supported by favorable non‐polar solvation and structural complementarity. Together, these findings identify QBP1 as a potent inhibitor of hIAPP aggregation in vitro and in silico, highlighting its potential as an anti‐amyloid strategy to protect β‐cells in T2D.

## 2 Materials and Methods

### 2.1 Peptide Preparation

Synthetic peptides—hIAPP (1–37; cat. #  RP11278), rat IAPP (rIAPP, 1–37), QBP1 (WKWWPGIF) [46, 47], SC‐M8 (WPIWKGWF) [37], SC‐M11 (WPIWSKGNDWF), a SC variant of the original 11‐residue QBP1 [32], and *Antp*‐QBP1 (RQIKIWFQNRRMKWKKGGWKWWPGIF) [40]—were synthesized by GenScript (Netherlands) at >95% purity. Following a disaggregation protocol, hIAPP and rIAPP were dissolved in formic acid (0.5 mg mL^−^^1^) and incubated for 1 h at room temperature (RT) [48], followed by 5 min sonication in a water‐bath sonicator. The solution was then aliquoted, dried under vacuum, and stored at −80°C. Stock solutions of QBP1, SC‐M8, SC‐M11 and *Antp*‐QBP1 were prepared in DMSO (10 mm) and stored at ‐20°C until use.

### 2.2 Amyloid Formation

Dried hIAPP or rIAPP peptide (0.5 mg) was reconstituted in 10 mm phosphate‐buffered saline (PBS, pH 7.4) with 2% DMSO and 2 mm sodium azide to a final concentration of 300 µM. rIAPP was included as a non‐amyloidogenic control peptide. After centrifugation at 16 000 x g for 50 min at 4°C to remove preformed aggregates, the supernatant (designated as “time zero”)—primarily populated by monomers—was used to initiate amyloidogenesis. Samples were vortexed and incubated at 25°C for 7 days without agitation to allow gradual formation of oligomers and fibrils. QBP1 and its SC variants were added at varying molar ratios at the beginning of the incubation. Aliquots were collected at different time points for biochemical characterization and β‐cells assays. For β‐cell assays involving extracellular hIAPP oligomers, samples were sonicated before use to disrupt early fibrils and enrich for oligomeric species [49, 50].

### 2.3 Thioflavin‐T (ThT) Fluorescence Assay

ThT fluorescence kinetics were monitored in vitro following established protocols [51, 52]. hIAPP (or rIAPP) stock solutions were diluted in PBS (pH 7.4) to the desired concentrations, resulting in final DMSO and sodium azide concentrations below 0.5% (v/v) and 0.02% (w/v), respectively. Reactions were prepared in TC‐treated 96‐well white plates with clear bottoms (Corning, cat. #CLS3610, Merck) containing the peptide at the indicated concentrations and 25 µM ThT (from a 1 mM stock). QBP1 and SC controls were added at five different molar ratios relative to hIAPP (including a 1:4 excess of QBP1). Vehicle controls contained matching DMSO and sodium azide concentrations. Fluorescence intensity (arbitrary units, *a.u*.) was monitored over 24–48 h at 30°C using a FLUOstar Omega spectrophotometer (BMG LABTECH), with excitation/emission at 450/482 nm. Data were analysed in OriginPro (OriginLab, Northampton, MA, USA) using non‐linear kinetic models to extract the maximum fluorescence intensity (MFI) and aggregation half‐time (t_1_/_2_), reflecting the extent and rate of ThT fluorescence development [52]. For fluorescence imaging of ThT‐positive structures, aliquots were collected after 24 and 48 h and imaged using a Leitz DM IRB microscope (Leica, Germany) equipped with 10×/0.22, 20×/0.30, and 40×/0.80 objectives. For higher‐resolution analysis, samples were transferred to Ibidi µ‐Slide 4 Well ibiTreat chambered coverslips (#1.5 polymer bottom, Ibidi GmbH, Germany) and imaged by confocal microscopy as described below.

### 2.4 Dot Blot Immunoassay

Two µL of incubated samples at various time points were spotted onto nitrocellulose membranes. After blocking for 1 h at RT with 10% non‐fat milk (Blotting‐Grade Blocker, Bio‐Rad) in Tris‐buffered saline containing 0.01% Tween 20 (TBS‐T), membranes were washed three times for 5 min with TBS‐T and incubated for 1 h at RT with the A11 polyclonal anti‐oligomer antibody (1:2000, Invitrogen, cat. # AHB0052) [53] and the fibril‐specific OC polyclonal antibody (1:2500, Millipore, cat. # AB2286) [54] in 5% milk‐TBS‐T. After three 5‐minute washes with TBS‐T, membranes were incubated for 1 h with the IRDye 680LT anti‐rabbit secondary antibody (LI‐COR) and then revealed in an Odyssey CLx (LI‐COR) imaging system. Final hIAPP (or rIAPP) concentration was 70 µM. Aβ_42_ fibrils (66 µM) and bovine serum albumin (BSA, 66 µM) were used as positive and negative controls, respectively.

### 2.5 Transmission Electron Microscopy (TEM)

For ultrastructural analysis, 10 µL of incubated samples were adsorbed onto carbon‐coated 300‐mesh copper grids (Ted Pella Inc.) for 5 min, followed by negative staining with a 2% (w/v) aqueous uranyl acetate for 5 min. Grids were washed twice with water and air‐dried. Images were acquired at accelerating voltages of 25–120 kV using either a JEOL JEM‐1011 transmission electron microscope equipped with a CCD MegaView III camera or a Thermo Fisher TALOS L120C transmission electron microscope operated at 120 kV and equipped with a CETA‐F camera. Samples were visualized after 72 h of incubation with 70 µM hIAPP (or rIAPP), with QBP1 tested at a five molar ratio relative to hIAPP.

### 2.6 Cell Culture and Viability Assays

Rat insulinoma–derived pancreatic β‐cell lines (INS‐1E, INS‐1E‐hIAPP, and INS‐1E‐Ct) were kindly provided by Dr. Anna Novials (IDIBAPS, Barcelona) and characterized as previously described [55, 56, 57]. INS‐1E‐hIAPP cells, which stably express human IAPP, were used as an overexpression model, whereas INS‐1E‐Ct cells transfected with an empty vector served as controls.

To assess the anti‐amyloidogenic effect of QBP1, we used its minimal active core (WKWWPGIF) fused to the Antp PTD (RQIKIWFQNRRMKWKK), enabling efficient receptor‐independent cellular uptake [38]. The fusion peptide was N‐terminally biotinylated and the two peptides were separated by two glycine residues as flexible linkers to minimize steric hindrance: Biotin‐RQIKIWFQNRRMKWKKGGWKWWPGIF.

– ***Cell culture***. Cells were maintained in RPMI 1640 medium supplemented with 10% (v/v) heat‐inactivated fetal bovine serum, 11 mM glucose, 10 mM HEPES, 2 mM L‐glutamine, 1 mM sodium pyruvate, 50 µM β‐mercaptoethanol, and 1% (v/v) penicillin‐streptomycin, at 37°C in a humidified atmosphere containing 5% CO_2_. INS‐1E‐hIAPP and INS‐1E‐Ct cells were cultured under identical conditions in the presence of 200 µg mL^−^^1^ Geneticin [57]. Culture media were refreshed every 2–3 days, and cells passaged at 80–90% confluence using trypsin‐EDTA. All reagents were purchased from Thermo Fisher Scientific (Gibco or Invitrogen brands), unless otherwise specified.
– ***Extracellular hIAPP conditions***. INS‐1E cells were cultured in high‐glucose medium (16.7 mm) to simulate T2D‐associated hyperglycemia [8, 58], seeded at 200 000 cells/mL in 96‐well plates, and incubated for 16 h before treatment. Cells were then exposed for 48 h to A11‐positive hIAPP oligomers or vehicle controls (buffer containing matched DMSO and sodium azide), with or without *Antp*‐QBP1. This approach enables precise control of hIAPP concentration and aggregation state, facilitating the study of oligomer‐dependent cytotoxic mechanisms and their modulation by *Antp*‐QBP1 [2, 10, 14, 59]. Viability was assessed using the CellTiter‐Glo Luminescent Cell Viability Assay (Promega, Madison, WI, USA), which quantifies ATP content as an indicator of metabolically active cells, and AlamarBlue (Invitrogen, Thermo Fisher), based on the reduction of resazurin to the fluorescent product resorufin in metabolically active cells, following the manufacturer's instructions. Luminescence (CellTiter‐Glo) and fluorescence (AlamarBlue; Ex 560 nm / Em 590 nm) were recorded using a FLUOstar Omega microplate reader (BMG LABTECH). Morphological changes were monitored by phase‐contrast microscopy (Leitz DM IRB, Leica, Germany) equipped with a Peltier temperature controller (Linkam, UK) set to 37°C and a Leica K5 camera. Images from at least three fields per condition were acquired at 10× and 20× magnification and analyzed with Leica LAS AF Lite software. For gene expression analysis, identical treatments were performed in 6‐well plates (Corning, cat. #3506) to ensure consistency.

– ***hIAPP overexpression conditions***. INS‐1E‐hIAPP and control (Ct) cells were seeded at 200 000 cells/mL in 96‐well plates (Corning, cat. #CLS3610, Merck) and incubated for 16 h before treatment. After 48 h of exposure to increasing concentrations of *Antp*‐QBP1, viability was assessed using CellTiter‐Glo assays following the manufacturer's instructions to evaluate the ability of *Antp*‐QBP1 to mitigate hIAPP‐induced cytotoxicity under intracellular overexpression conditions.

### 2.7 Immunocytochemistry (ICC)

Cells were seeded in 24‐well plates (Corning, cat. #3526) on 12 mm coverslips pre‐treated with 1 mg/mL poly‐D‐lysine (Merck, cat. #P2636) in borate buffer (pH 8.0) at a density of 200,000 cells/mL. After 48 h in culture medium containing 10 µM biotin‐tagged *Antp*‐QBP1 [38], alone or co‐incubated with 10 µM exogenous hIAPP oligomers, or vehicle control, cells were fixed with 4% paraformaldehyde (Sigma‐Aldrich) for 15 min at RT, permeabilized with 0.1% Triton X‐100 in PBS for 15 min, and blocked with 2% BSA and 0.1% Triton‐X100 in PBS for 1 h at RT. Primary antibodies against hIAPP (rabbit anti‐hIAPP, 1:400; Sigma‐Aldrich), biotin (goat anti‐biotin, 1:200; Invitrogen, cat. # 31852), oligomers (A11, 1:400, Invitrogen, cat. # AHB0052) and fibrils (OC, 1:200, Millipore, cat. # AB2286) were applied overnight at 4°C. Fluorophore‐conjugated secondary antibodies (Invitrogen) were added for 1 h at RT in darkness, followed by DAPI nuclear staining for 15 min. Coverslips were mounted with ProLong Gold (Life Technologies) and imaged using either a Leica DMI 6000 fluorescence microscope or a Leica Stellaris 8 stimulated emission depletion (STED) super‐resolution microscope, both equipped with a 40× oil‐immersion objective.

### 2.8 Confocal Microscopy and Image Analysis

ThT‐stained and ICC samples were imaged using a Leica Stellaris 8 STED super‐resolution confocal microscope (Leica Microsystems, Germany). For ThT imaging, samples in Ibidi μ‐Slide 4 Well ibiTreat coverslips were excited at 488 nm and emission was collected between 509–580 nm. For ICC, fluorophores were excited at 405 nm (DAPI; 420–480 nm, HyD S1), 488 nm (Alexa Fluor 488/FITC; 500–550 nm, HyD S2) and 653 nm (Alexa Fluor 647; 665–750 nm, HyD X3), all acquired sequentially in counting mode to prevent spectral overlap. Images were acquired at 1024×1024 pixels with a line average of 3, frame average of 1, scan speed of 600 Hz, and pixel dwell time of 875 ns. Z‐stacks were collected in optimized mode to generate 3D reconstructions of ThT fluorescence and ICC labeling. Image processing and quantitative analysis were conducted using ImageJ/Fiji (NIH, USA). Specifically, a custom Fiji macro based on the “Find Maxima” algorithm was implemented to detect local intensity peaks across the selected fluorescence channels. Fluorescence intensity per cell (*a.u*.) was calculated as the total signal per field divided by the number of DAPI‐positive nuclei, and the number of fluorescence spots per cell was defined as total local maxima per field normalized to nuclei count. The macro script (*find_maxima_batch_vfin_yesss.py*) is available from the authors upon reasonable request.

### 2.9 Gene Expression Analyses

– *RNA isolation*. Total RNA from hIAPP‐treated cells was isolated using the NzyTech Total RNA Tissue Kit (NZYtech) according to the manufacturer's protocol, including on‐column DNase digestion (RNase‐Free DNase Set, Promega). RNA quality and concentration were assessed using a Nanodrop One spectrophotometer (Thermo Fisher Scientific).
– *RT‐qPCR*. One µg of total RNA was reverse‐transcribed into complementary DNA (cDNA) using the High‐Capacity cDNA Reverse Transcription Kit (Thermo Fisher). Quantitative PCR reactions were performed using 10 ng of cDNA per reaction using SYBR Green Reagents (NZYtech) on a QuantStudio 3 RQ‐PCR System (Thermo Fisher), following the manufacturer's instructions. Primers for *Ccl2*, *Il1b*, *Slc2a2*, *Hspa5*, and *18S rRNA* (used as the reference housekeeping gene) were obtained from Merck KGaA (Darmstadt, Germany) and are listed in Table S1. Relative gene expression levels were calculated using the 2^−ΔΔCt^ method, normalizing target gene expression to 18S rRNA and expressing values relative to the control condition from at least three independent experiments. Fold‐change values were log_2_‐transformed for graphical representation and statistical analysis, centering the data around zero (log_2_FC = 0 indicates no change) and enabling a symmetrical representation of up‐ and down‐regulation while preserving the quantitative relationships between samples.

### 2.10 Statistical Analysis

Data are presented as mean ± standard error of the mean (SEM) from at least three independent experiments. Statistical comparisons were performed using one‐way or two‐way analysis of variance (ANOVA), as appropriate, with post hoc tests specified in each figure legend. Normality and homogeneity of variance were verified prior to statistical testing. Differences were considered statistically significant at *p* < 0.05. Analyses and graphical representations were generated using GraphPad Prism (version 8.00; GraphPad Software, La Jolla, CA, USA) and OriginPro (OriginLab, Northampton, MA, USA).

### 2.11 Circular Dichroism

Far‐UV CD spectra were recorded on samples of hIAPP with a concentration of 50–100 µM, in the presence or absence of 200–500 µM QBP1, at 37°C in PBS (130 mm NaCl, 8 mm K_2_HPO_4_/ 2 mm KH_2_PO_4_, pH 7.4) on a JASCO J‐810 spectropolarimeter equipped with a Peltier temperature control unit. Because DMSO absorbs strongly in the far‐UV region, samples were initially dissolved in 10 µL of hexafluoroisopropanol (HFIP) prior to dilution with PBS. Treatment with HFIP was previously reported to monomerize hIAPP [60]. Spectra were recorded from 198 to 260 nm using a 1.5 nm bandwidth, a scan speed of 50 nm·min^−1^, and averaging 8–10 scans per spectrum.

### 2.12 Nuclear Magnetic Resonance Spectroscopy

– *hIAPP/QBP1 interaction experiments*. 1D ^1^H and 2D ^1^H‐^1^H total correlation spectroscopy (TOCSY) (mixing time = 60 ms) and 2D ^1^H‐^1^H nuclear Overhauser effect spectroscopy (NOESY) (mixing times = 80 and 250 ms) spectra were recorded at 10°C in 90% H_2_O/ 10% D_2_O with 1 mm K_2_HPO_4_ and 1 mm sodium acetate buffer (pH 5.5) on 100 µM hIAPP (pre‐monomerized in a small volume of hexadeuterated DMSO) on a Bruker 800 MHz (^1^H) spectrometer equipped with a ^1^H, ^13^C, ^15^N cryoprobe, Neo console and z‐gradients. These conditions were chosen because they were previously reported [61] to greatly slow the aggregation of hIAPP, affording time to allow the spectra to be recorded. The solutions also contained 100 µM DSS as the internal chemical shift reference. Spectra were assigned with the aid of the program POKY [62], and the assignments have been deposited in the BMRB database; with access code = 53405. Next, additional spectra were recorded under the same conditions, but in the presence of 500 µM of QBP1. The spectra were then analyzed for changes which could reveal atomic‐level details on the interaction between hIAPP and QBP1.
– *hIAPP aggregation experiments*. A second set of NMR experiments were performed in PBS (90% H_2_O/ 10% D_2_O) at 37°C. These conditions were shown to promote hIAPP aggregation [63]. These experiments were performed on a Bruker 600 MHz (^1^H) spectrometer equipped with a ^1^H, ^13^C, ^15^N, ^31^P cryoprobe, Neo console, and z‐gradients. The final sample pH was verified in situ based on the phosphate ^3^ ^1^P chemical shift [64].

### 2.13 Molecular Docking and Molecular Dynamics Simulations

We examined the binding between human IAPP (hIAPP; 37‐residue peptide with a Cys2–Cys7 disulfide bond; sequence *KCNTATCATQRLANFLVHSSNNFGAILSSTNVGSNTY*) and the QBP1‐derived inhibitors: the octapeptide QBP1 (*WKWWPGIF)* [45], a hydrophobic‐core mutant (*WKAAPGIF*; two W→A substitutions within the WKWW motif), and the SC variants SC‐M11 (*WPIWSKGNDWF)* and SC‐M8 (*WPIWKGWF*). The NMR structure of hIAPP in sodium dodecyl sulfate micelles at pH 7.3 **(PDB: 2L86** [65]; Figure S1A) was used as the starting model. In parallel, rat IAPP (rIAPP; with key substitutions at Pro25/Pro28/Pro29; sequence *KCNTATCATQRLANFLVRSSNNLG* ***P*** *VL* ***PP*** *TNVGSNTY*), a non‐amyloidogenic isoform **(PDB: 2KJ7** [66]; Figure S1B), was included as a negative control target. QBP1 and SC‐M8 were also docked and simulated against rIAPP following the same protocol. The initial QBP1 structure was taken from the experimental coordinates of Ramos‐Martín et al. [45], while WKAAPGIF and SC variants were obtained by all‐atom MD pre‐sampling.

Complexes were generated with HADDOCK [67] using N‐acetylated/C‐amidated peptides. For each peptide (and isoform), the top‐10 docking poses were propagated to explicit‐solvent MD with the AMBER20 package [68], the ff19SB force field [69] and OPC water at 0.15 M NaCl [70]. After standard minimization, heating, and NVT/NPT equilibration at 300 K [71], 10 × 100 ns trajectories per pose were run (2 fs time step, SHAKE on bonds to H, Langevin thermostat 2 ps^−^^1^, PME electrostatics; ≥10 Å solvent padding; frames saved every 10 ps). Trajectories were analyzed to derive residue‐to‐residue contact maps [72, 73, 74] and to estimate binding free energies using the Molecular Mechanics/Poisson–Boltzmann Surface Area (MM/PBSA) approach with normal‐mode entropy [75]. The most populated binding modes are reported. Full simulation parameters are provided in the Supporting Information (Molecular docking and molecular dynamics simulations).

## 3 Results

### 3.1 QBP1 Outperforms SC Variants in Inhibiting Aggregation, Delaying Nucleation, and Reducing Fibril Formation of hIAPP

**FIGURE 1:**
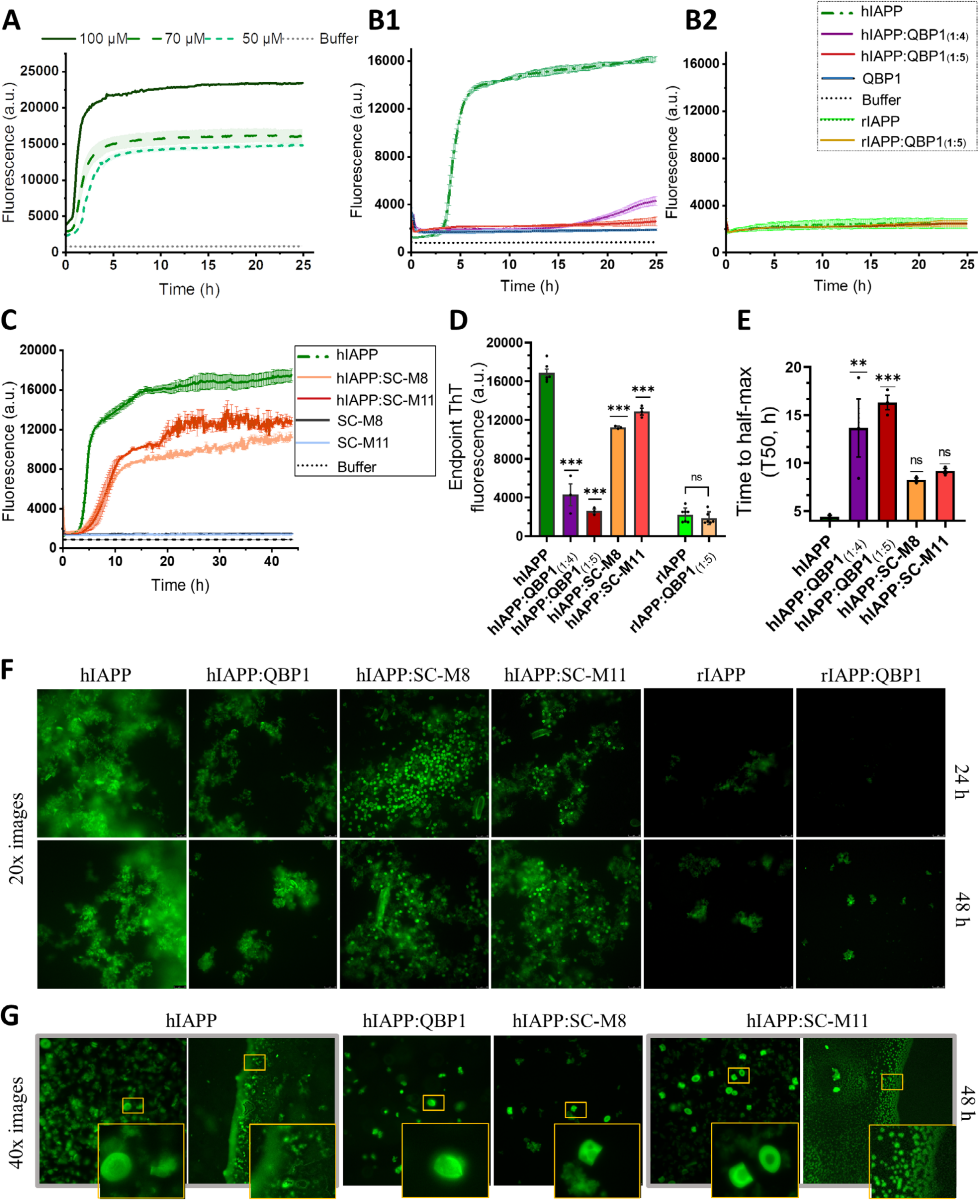
Inhibition of hIAPP amyloid fibril formation by QBP1 and its scrambled variants. (A) ThT fluorescence kinetics of hIAPP at increasing concentrations (50, 70, and 100 µM) over 24 h. Higher concentrations accelerated aggregation, reducing the lag phase and increasing plateau fluorescence. Based on these data, 70 µM was selected for subsequent assays (panels B–G). (B1) Aggregation kinetics of hIAPP in the presence of QBP1 (1:4 and 1:5 molar ratios). QBP1 inhibited fibril formation from the earliest stages, with near‐complete suppression at the 1:5 ratio. (B2) Aggregation kinetics of rIAPP in the absence or presence of QBP1 (1:5). rIAPP produced only near‐baseline ThT fluorescence, and QBP1 did not modify this profile, consistent with the non‐amyloidogenic nature of rIAPP. (C) Aggregation kinetics of hIAPP in the presence of scrambled control peptides (SC‐M8 and SC‐M11, 1:5 molar ratio). Both variants partially inhibited aggregation but still allowed fibril formation. (D) Quantification of plateau ThT fluorescence (24 h for QBP1 conditions; 48 h for SC peptides). QBP1 significantly reduced fluorescence vs. hIAPP alone (***p < 0.001), with the lowest levels observed at 1:5. SC‐M8 and SC‐M11 also reduced endpoint fluorescence (***p < 0.001), albeit to a lesser extent. rIAPP samples remained at near‐baseline levels with no significant difference upon QBP1 addition (not significant, *ns*). (E) Aggregation half‐time (T_50_). QBP1 significantly delayed aggregation (**p = 0.004 at 1:4; ***p < 0.001 at 1:5), whereas SC peptides had no significant effect (ns). Statistical comparisons in (D) and (E) were performed using one‐way ANOVA followed by Dunnett's post hoc test vs. hIAPP alone, while the comparison between rIAPP and rIAPP+QBP1 conditions was assessed separately using an unpaired two‐tailed Student's t‐test. (F) Fluorescence microscopy (20×) of ThT‐stained samples at 24 and 48 h of incubation. hIAPP alone accumulated abundant ThT‐positive aggregates; QBP1 (1:5) markedly reduced fluorescence, while SC peptides (1:5) showed intermediate levels. rIAPP samples displayed only minimal ThT fluorescence both alone and with QBP1. (G) Fluorescence microscopy (40×, 48 h). hIAPP formed dense fluorescent clusters, while QBP1 co‐incubation reduced aggregate clustering. Both conditions showed small punctate structures (*top insets, first and second columns*). SC variants (1:5) produced ring‐like structures (*top insets, third and fourth columns*), and SC‐M11 occasionally formed droplet‐like features suggestive of phase separation (*bottom inset, fourth column*). Insets show magnified regions. Panels (F–G) provide qualitative visualization supporting the quantitative ThT data shown in (D–E). Images are representative of at least three independent experiments.

We evaluated the in vitro amyloidogenic potential of hIAPP and the ability of QBP1 and its SC variants (SC‐M8; SC‐M11) [32, 37] to inhibit this process. Aggregation kinetics and endpoint fibril formation were monitored using ThT‐based fluorescence assays over 24–48 h (Figure 1). No signal was detected in buffer controls, confirming ThT specificity. As shown in Figure 1A, hIAPP aggregation followed a characteristic sigmoidal profile with a short lag phase (∼3 h) consistent with the presence of monomers and early oligomers, followed by a rapid growth phase (∼3–9 h) associated with fibril elongation, and a plateau (∼10 h) corresponding to mature fibril formation. Aggregation was clearly concentration‐dependent, with higher hIAPP levels (50, 70, and 100 µM) leading to shorter lag phases and higher plateau fluorescence, consistent with faster nucleation and increased fibril yield.

In contrast, Figure 1B1 shows that co‐incubation with QBP1 at 1:4 and 1:5 molar ratios (hIAPP:QBP1) resulted in a pronounced reduction in amyloid formation. At a 1:5 ratio, ThT fluorescence remained at near‐basal levels throughout the assay, consistent with the absence of detectable fibrils. This effect was apparent from the earliest time points, suggesting an effect at early stages of aggregation. QBP1 alone did not produce any ThT signal. Quantification of ThT fluorescence at the 24‐h plateau (Figure 1D; see Figure S2 for experimental controls) confirmed a significant reduction in fibril accumulation at both ratios vs. hIAPP alone (*p* < 0.001), with the lowest signal observed at 1:5. Consistently, T_50_ analysis (Figure 1E, T_50_ defined as the time to reach 50% of maximum fluorescence) revealed a significant delay at 1:4 (*p* = 0.004), whereas fluorescence at 1:5 remained at baseline, precluding a sigmoidal fit. Although a statistical difference was detected (*p* < 0.001), this reflects near‐complete inhibition rather than a measurable kinetic delay. No effect was detected below the 1:4 ratio (data not shown). Together, these findings indicate a threshold‐like inhibitory effect, with robust suppression of hIAPP aggregation observed only at molar ratios ≥ 1:4 (hIAPP:QBP1).

To verify that the observed inhibitory effect of QBP1 is specifically linked to the amyloidogenic behavior of hIAPP, we included rodent IAPP (rIAPP), a non‐amyloidogenic ortholog commonly used as a negative control due to the presence of proline substitutions that disrupt β‐sheet formation. As shown in Figure 1B2,D, rIAPP tested at 70 µM under the same experimental conditions produced only near‐baseline ThT fluorescence, consistent with its known inability to form amyloid fibrils. Co‐incubation with QBP1 at the most effective inhibitory ratio identified for hIAPP (1:5) did not modify this profile, confirming that QBP1 does not alter ThT fluorescence in the absence of amyloidogenic aggregation. These results support that the effect of QBP1 observed in hIAPP samples is associated with suppression of the aggregation process rather than with nonspecific interference with the ThT assay.

SC‐M8 and SC‐M11 were tested as control peptides in hIAPP aggregation assays at a 1:5 molar ratio. Both variants partially inhibited aggregation (Figure 1C), yielding reduced ThT fluorescence compared with hIAPP alone but still allowing fibril formation. Neither SC peptide produced a ThT signal on its own. Quantification at the 48‐h plateau confirmed a significant reduction in fibril accumulation for both controls (*p* < 0.001, Figure 1D); however, their effects on T_50_ were minimal and not statistically significant (Figure 1E), consistent with a limited impact on aggregation kinetics. Non‐linear Boltzmann fitting [52]—not applicable to QBP1 curves due to the absence of a sigmoidal profile—revealed that SC variants moderately reduced the maximum fluorescence intensity and slightly increased the aggregation half‐time (t_1_/_2_: hIAPP 4.82 h; SC‐M8 6.67 h; SC‐M11 8.21 h) (Figure S3). These results indicate a modest inhibitory effect of the SC variants on hIAPP aggregation, highlighting the importance of sequence and structural determinants in inhibitory efficacy compared with QBP1.

Fluorescence microscopy (20×) was used to visualize ThT‐stained samples after 24 and 48 h of incubation with or without the inhibitory peptides (Figure 1F). hIAPP alone showed abundant ThT‐positive signal that intensified and clustered over time, consistent with progressive amyloid aggregation [76, 77]. Due to the limited resolution of light microscopy, the ultrastructural organization of the aggregates could not be resolved; therefore, TEM was employed to confirm the amyloid nature of the observed assemblies (*see next section*). Co‐incubation with QBP1 (1:5) markedly reduced both the intensity and clustering of ThT‐positive signal, whereas SC‐M8 and SC‐M11 produced intermediate levels—lower than hIAPP alone but higher than QBP1—mirroring their partial inhibitory activity. In contrast, rIAPP showed near‐baseline ThT fluorescence under the same conditions, both alone and with QBP1. These observations are in agreement with the kinetic and endpoint ThT fluorescence data (Figure 1B–E). Additional low‐magnification (10×) images including all experimental controls are provided in the Figure S4.

To further assess ThT signal distribution, samples were imaged at 40× magnification using Ibidi μ‐Slides (Figure 1G). hIAPP alone formed dense fluorescent clusters, whereas QBP1 co‐incubation resulted in a weaker and more dispersed ThT signal. In both conditions, small punctate fluorescent spots were observed (*top insets, first and second columns*), which may correspond to early or off‐pathway hIAPP assemblies. Distinctively, treatments with SC‐M8 and SC‐M11 produced more defined fluorescent structures with sharp boundaries (*top insets*, third and fourth columns), including ring‐like features and, in the case of SC‐M11, larger droplet‐like areas near sample edges reminiscent of hIAPP phase separation (*bottom inset, fourth column*) [76, 78]. Confocal microscopy and 3D reconstructions (Figure S5) confirmed that these distinct fluorescence patterns persisted for up to 7 days.

Together, these results demonstrate distinct effects of QBP1 and SC variants on ThT‐detectable hIAPP assemblies, with robust suppression of amyloid formation by QBP1 and persistence of compact, ThT‐positive assemblies in the presence of SC variants.

### 3.2 QBP1 Attenuates Oligomer Formation and Limits Fibril Assembly of hIAPP

**FIGURE 2:**
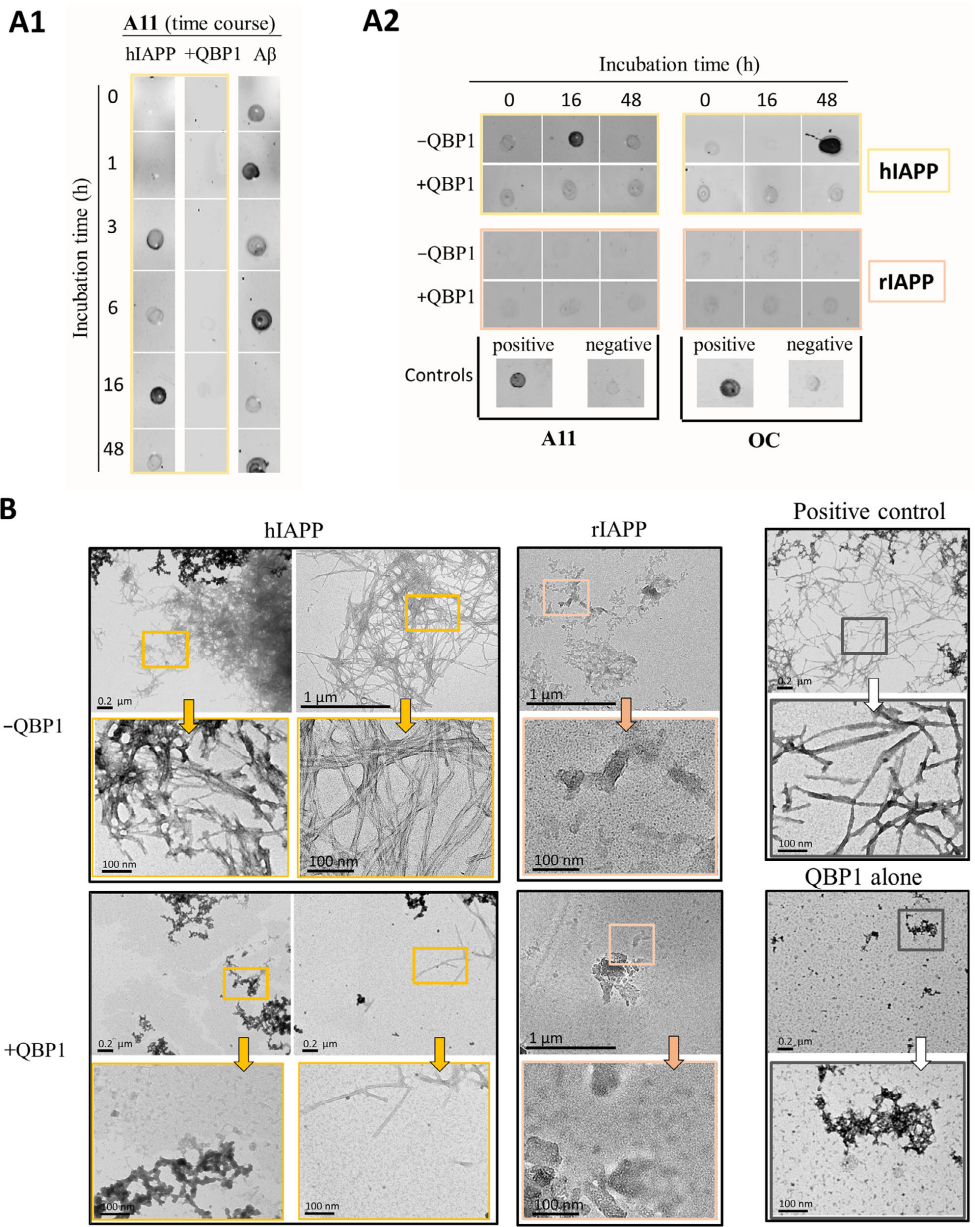
QBP1 disrupts hIAPP aggregation by attenuating oligomer formation and limiting fibril assembly. (A1) Time‐course immunodot blot analysis of hIAPP (70 µM) incubated with or without QBP1 (1:5) and probed with the oligomer‐specific antibody A11. A11‐positive species became detectable at early time points and peaked at ∼16 h in hIAPP samples, whereas QBP1 co‐incubation markedly reduced oligomer immunoreactivity throughout the time course. (A2) Immunodot blot analysis of hIAPP and rIAPP (70 µM), incubated with or without QBP1 (1:5) and probed with A11 (oligomer‐specific) or OC (fibril‐specific) antibodies at different time points (0, 16, and 48 h). hIAPP showed A11‐positive oligomers at intermediate time points and OC‐positive fibrillar assemblies at later stages, both markedly reduced by QBP1 co‐incubation. In contrast, rIAPP remained negative for both antibodies under all conditions. Controls include BSA (negative, 70 µM) and Aβ42 fibrils (positive, 70 µM). (B) Representative TEM images of hIAPP and rIAPP incubated for 72 h in the absence or presence of QBP1 (1:5). hIAPP alone formed dense fibrillar networks organized into tightly packed clusters, whereas co‐incubation with QBP1 markedly reduced fibril formation, yielding sparse short fibrils and amorphous aggregates. In contrast, rIAPP did not form fibrillar structures, displaying only sparse amorphous material both in the absence and presence of QBP1. α‐Synuclein fibrils were included as a positive control for amyloid fibril morphology, while QBP1 alone produced only amorphous, non‐fibrillar material. Insets highlight regions selected for higher magnification (100 nm). TEM images were acquired using two different microscopes, resulting in slight differences in scale bar appearance. Scale bars: 0.2 or 1 µm (upper panels) and 100 nm (lower panels). Images are representative of *n = 3* independent experiments.

To assess the impact of QBP1 on early and late amyloidogenic intermediates, we performed immunodot blot analyses using two conformation‐specific antibodies: A11, which selectively recognizes prefibrillar oligomers [53], and OC, which detects fibrillar oligomers and mature fibrils [54]. As shown in Figure 2A, hIAPP alone shifted from A11‐positive species at early time points to OC‐positive assemblies at later stages, consistent with a progression from prefibrillar intermediates to mature fibrils. A11 binding became detectable at ∼3 h (Figure 2A1) and peaked at ∼16 h, whereas OC reactivity appeared later, at ∼48 h (Figure 2A2). Aggregation kinetics observed by dot blot appeared slightly delayed compared with ThT‐based assays, probably due to methodological differences: ThT reactions are performed under constant agitation in a microplate format, which accelerates aggregation, whereas dot blot samples are incubated statically in larger volumes and undergo manual processing. Thus, both approaches report the same amyloidogenic pathway, albeit on different time scales.

In contrast, co‐incubation with QBP1 (1:5, hIAPP:QBP1) markedly reduced both A11 and OC immunoreactivity at all time points examined. Even at ∼16 h—the peak oligomer signal in hIAPP alone—A11 binding remained undetectable in the presence of QBP1, consistent with an effect of QBP1 at early stages of hIAPP aggregation and concomitant reduction in later fibrillar species. Consistent with its non‐amyloidogenic nature, rIAPP did not show detectable A11 or OC immunoreactivity under the same experimental conditions, either alone or in the presence of QBP1, confirming the absence of oligomeric and fibrillar assemblies. Control conditions (QBP1 alone and BSA) showed no reactivity with any of the antibodies, confirming assay specificity.

After 7 days of incubation, A11 and OC immunoreactivity remained strongly reduced in all peptide‐treated samples compared with hIAPP alone, including QBP1, SC‐M8, and SC‐M11 (1:5) (Figure S6). At this late time point, antibody reactivity converged toward similarly low levels across all peptide‐containing conditions. Consistent with their ThT fluorescence patterns, the weak A11/OC signals observed in SC‐treated samples are compatible with the presence of compact, ThT‐positive assemblies displaying reduced accessibility to conformational antibody epitopes, while still permitting partial hIAPP self‐association.

To directly assess the ultrastructural outcome of these aggregation pathways, TEM was performed after 72 h of incubation at multiple magnifications (Figure 2B). hIAPP alone formed dense networks of mature fibrils organized into tightly packed clusters, similar to those observed for α‐synuclein fibrils used as a positive control. In contrast, co‐incubation with QBP1 (1:5) resulted in a marked reduction in fibril formation, yielding only sparse short fibrils and poorly ordered or amorphous aggregates rather than extended amyloid networks. Consistent with the dot blot and ThT analyses, rIAPP samples did not form fibrillar structures, displaying only sparse amorphous aggregates both in the absence and presence of QBP1. QBP1 alone produced exclusively amorphous, non‐fibrillar material, confirming that it does not self‐assemble into amyloid‐like structures under these conditions.

Taken together, these results indicate that QBP1 reduces the accumulation of early oligomeric intermediates and mature fibrillar assemblies during hIAPP aggregation.

### 3.3 QBP1 Arrests the Conformational Transition of hIAPP from Random Coil to β‐sheet

**FIGURE 3:**
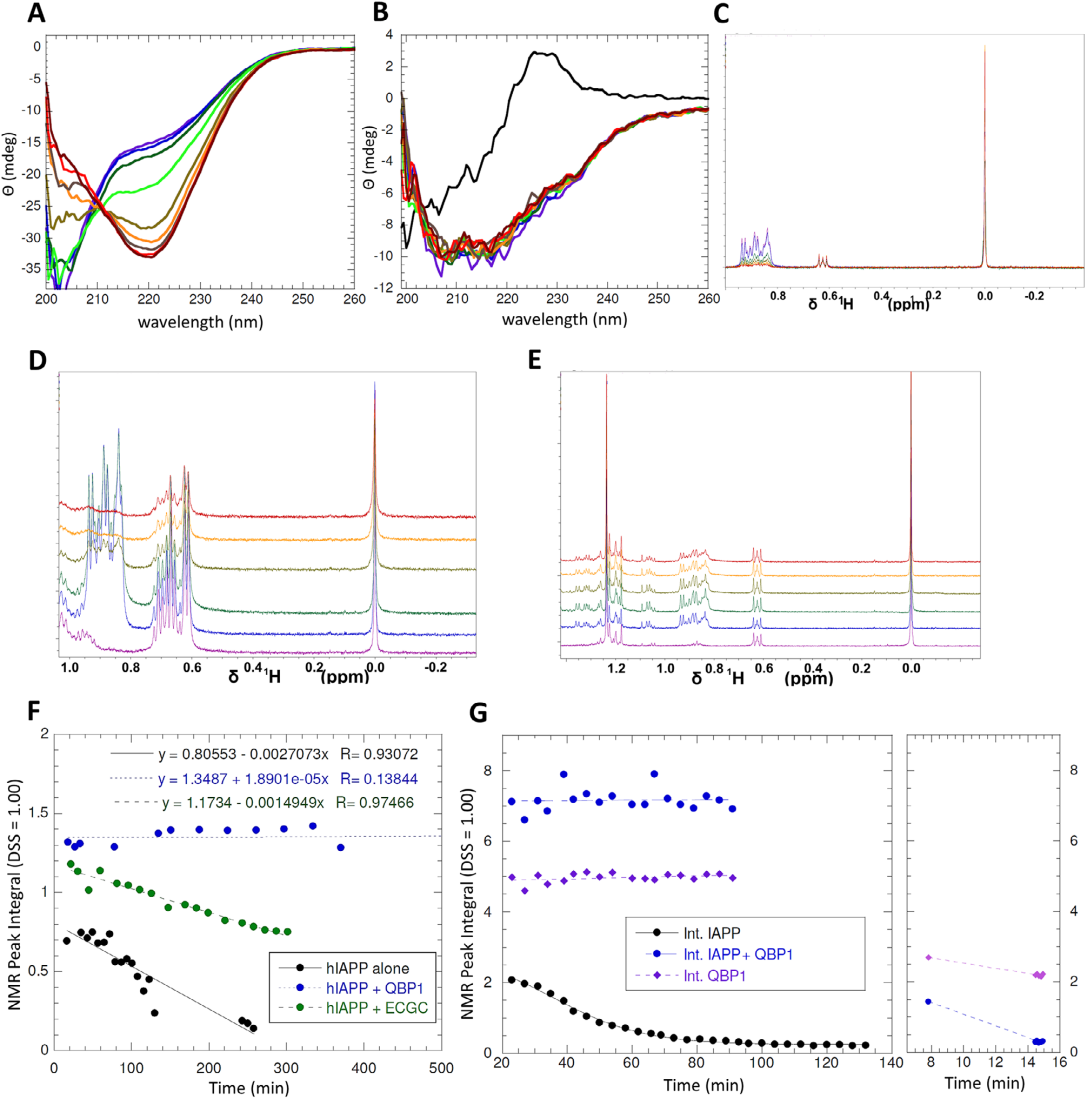
CD and NMR analyses reveal that QBP1 arrests hIAPP conformational conversion and suppresses oligomerization. Far‐UV CD spectroscopy (A & B) and solution‐state NMR spectroscopy (C, D, E, F, & G) were used to monitor hIAPP conformational changes and oligomerization kinetics in the absence or presence of QBP1. (A) Far‐UV CD spectra of 50 µM hIAPP in PBS recorded at 37°C after 4 (violet), 13 (blue), 23 (dark green), 33 (light green), 43 (olive), 50 (orange), 58 (brown), 68 (red), and 81 (maroon) minutes. The initial minimum at ∼202 nm (statistical coil) progressively shifts toward a β‐sheet‐like minimum near 220 nm. (B) Far‐UV CD spectra of 250 µM QBP1 recorded at 37°C alone (black) or in the presence of 50 µM hIAPP at 7 (purple), 24 (blue), 39 (dark green), 55 (light green), 70 (olive), 100 (orange), 114 (brown), 129 (red), and 148 (maroon) minutes after mixing. (C) One‐dimensional (1D) ^1^H NMR spectra of hIAPP (100 µM) in PBS at 37°C (peaks between 1.00 & 0.80 ppm) and 100 µM DSS (trimethyl singlet at 0.00 ppm, methylene triplet at 0.62 ppm), recorded after 23 (purple), 32 (blue), 51 (green), 70 (olive), 94 (orange), and 128 (red) minutes after mixing. Note that the hIAPP peaks decrease over time, whereas the DSS peaks remain unchanged. (D) 1D ^1^H NMR spectra of QBP1 (500 µM, signals between 0.75 and 0.60 ppm) and hIAPP (100 µM) (peaks between 1.00 & 0.80 ppm) and 100 µM DSS (trimethyl singlet at 0.00 ppm, methylene triplet at 0.62 ppm), recorded for QBP1 alone (purple) and at 9 (blue), 91 (green), 470 (olive), 869 (orange), and 1140 (red) minutes after mixing with hIAPP in PBS at 37°C. (E) 1D ^1^H NMR spectra of 150 µM green tea polyphenol EGCG (strong singlet at 1.23 ppm), 50 µM hIAPP (peaks between 1.00 & 0.80 ppm) and 100 µM DSS (trimethyl singlet at 0.00 ppm, methylene triplet at 0.62 ppm), recorded for EGCG alone (purple), and at 22 (blue), 45 (green), 111 (olive), 184 (orange), and 302 (red) minutes after mixing EGCG with hIAPP in PBS at 37°C. (F) Time‐dependent decay of hIAPP 1D ^1^H NMR peak intensities (normalized to DSS) for 50 µM hIAPP alone (black), or in the presence of 150 µM EGCG (green) or 150 µM QBP1 (blue). The lines show the fit of a linear equation to the data. (G) Time‐dependent decay of hIAPP 1D ^1^H NMR peak intensities (relative to the trimethyl resonance of DSS) of 100 µM of hIAPP alone (black) or in the presence of 500 µM QBP1 (blue). The evolution of the QBP1 resonances is shown using blue symbols. Solid lines indicate linear fits.

The far‐UV CD spectra of hIAPP (50 µM in PBS, at 37°C) (Figure 3) initially display spectral features typical of a statistical coil ensemble; namely, a minimum near 202 nm. The lack of a double minima at 208 and 222 nm rules out the presence of significant populations of *α*‐helices (Figure 3A). In subsequent spectra recorded over the next 2 h, the minimum at 202 nm weakens and a new minimum near 220 nm, which is indicative of β‐sheet, becomes increasingly stronger. These results closely resemble previously published spectra of hIAPP recorded under very similar conditions [63].

In a separate experiment, the far‐UV CD spectrum of QBP1 alone (250 µM in PBS, at 37°C) was recorded. This spectrum exhibited a positive CD band near 228 nm, which can be ascribed to a fixed conformation of its aromatic residues [45]. Subsequently, 50 µM hIAPP dissolved in HFIP was added to this QBP1 solution, and a series of CD spectra was recorded for 2.5 h (Figure 3B). The spectral features of this sample are different from QBP1, hIAPP or their sum. Whereas it is difficult to interpret that difference in terms of secondary structure, due to the contribution of aromatic groups to the far UV CD spectrum, it is important to note that these spectra remain largely the same over this time period. This result strongly suggests that QBP1 has arrested the conformational evolution of the hIAPP from random coil to β‐sheet.

To further probe whether this conformational arrest involves direct interactions with monomeric hIAPP, we next examined hIAPP by solution NMR spectroscopy. The assigned ^1^HN‐^1^Hα region of the 2D TOCSY and NOESY NMR spectra are shown in Figure S7A. They show that the ^1^HN chemical shifts occupy a narrow range of chemical shift values which is consistent with a statistical coil conformational ensemble. Additional NMR spectra were recorded in the presence of increasing concentrations of QBP1, nevertheless in these spectra, no substantial changes in the chemical shifts or linewidths of resonances from QBP1 or hIAPP could be detected (Figure S7B,C). Liquid‐state NMR spectroscopy is strongly limited by molecular size; once hIAPP oligomers grow to over 50 kDa, they become invisible to this technique. Previous NMR studies of Aβ binding to small peptide inhibitors [79] or α‐synuclein binding to EGCG at protein to ligand concentrations <60 [80] also did not observe chemical shift or linewidth changes and attributed their results to those inhibitors binding to large oligomer species of Aβ or α‐synuclein. Likewise, the lack of observed chemical shift or linewidth changes could suggest that QBP1 interacts with early aggregated hIAPP species—likely oligomeric intermediates—rather than the monomeric peptide. Alternatively, it is also possible that QBP1 binds weakly, transiently and heterogeneously to hIAPP as this kind of interaction would not produce significant changes in NMR chemical shifts or linewidths.

We next monitored the time‐dependent loss of hIAPP NMR signal intensity under aggregation‐prone conditions to evaluate the effect of QBP1 on oligomerization kinetics. When incubated alone at 100 µM in PBS at 37°C, hIAPP NMR signals progressively decreased over the course of several hours, consistent with the formation of oligomeric species with slow tumbling times that escape detection by solution NMR (Figure 3C). This is consistent with the CD experiments performed at 37°C in PBS (vide supra). In the presence of excess QBP1, however, this process is much slowed and is still incomplete after several hours (Figure 3D).

In a complementary experiment, we followed the loss of 50 µM hIAPP NMR signals when alone, or in the presence of a three‐fold excess of epigallocatechin‐3‐gallate (EGCG, a well‐established hIAPP aggregation inhibitor used as a positive control and benchmarking) or a three‐fold excess of QBP1 (Figure 3E). Here, whereas EGCG slowed the rate of hIAPP oligomerization by almost 50%, QBP1 essentially completely blocked hIAPP oligomerization over the monitored time course (Figure 3F). At higher peptide concentration (100 µM), hIAPP aggregates more quickly (Figure 3G), yet a five‐fold excess of QBP1 still suppressed oligomerization for several hours.

### 3.4 Antp‐QBP1 Retains the Inhibitory Activity of QBP1 and Is Efficiently Internalized by INS‐1E β‐Cells

**FIGURE 4:**
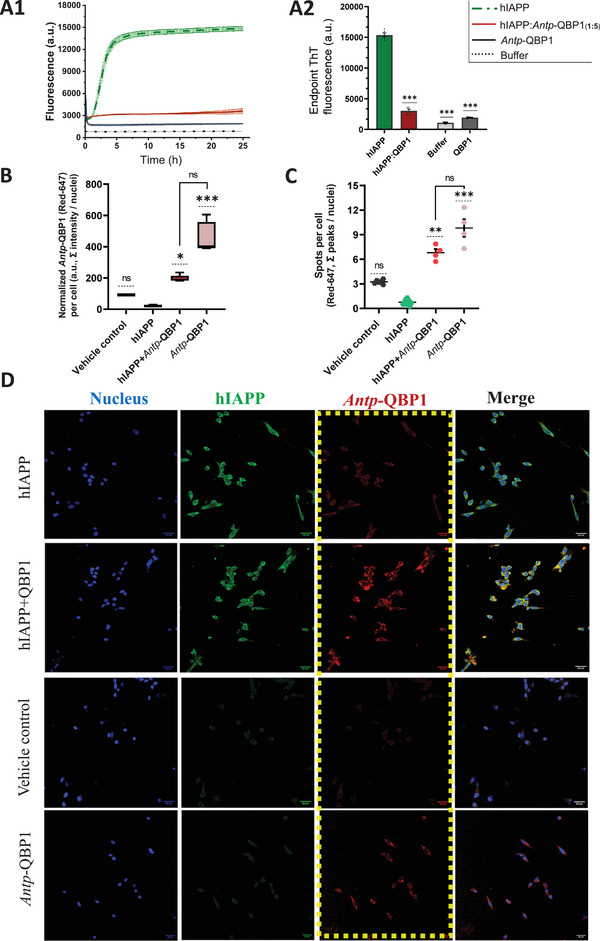
*Antp*‐QBP1 retains inhibitory activity and allows intracellular delivery. (A) ThT fluorescence kinetics of hIAPP (70 µM) alone or with *Antp‐*QBP1 (1:5). (A1) *Antp*‐QBP1 suppressed amyloid formation comparably to QBP1, with fluorescence remaining near baseline; *Antp*‐QBP1 alone showed no ThT signal. (A2) Endpoint ThT fluorescence at 24h, confirming significant reductions with *Antp*‐QBP1 (***p < 0.001). Data are mean ± SEM; statistical comparisons were performed by one‐way ANOVA with Dunnett's post hoc test vs. hIAPP; data represent at least three independent experiments. (B‐D) ICC of INS‐1E β‐cells incubated for 48 h with vehicle, hIAPP (10 µM), *Antp*‐QBP1 (10 µM), or hIAPP+*Antp*‐QBP1. Blue: nuclei (DAPI); green: hIAPP (anti‐IAPP, FITC channel); red: *Antp*‐QBP1 (anti‐biotin, Red‐647 channel). (B) Quantification of anti‐biotin (Red‐647) fluorescence intensity per cell (*Σ intensity/DAPI nuclei*). *Antp*‐QBP1 alone markedly increased the intracellular signal vs. hIAPP alone (***p < 0.001). Co‐treatment with hIAPP also resulted in significant uptake (*p = 0.015), although levels were lower than with *Antp*‐QBP1 alone (*ns*). (C) Quantification of Red‐647 fluorescence spots per cell (*Σ peaks/DAPI nuclei*). *Antp*‐QBP1, either alone (***p < 0.001) or with hIAPP (**p = 0.005), showed a significantly higher number of intracellular spots than hIAPP alone. No differences were detected between *Antp*‐QBP1 alone and co‐administration conditions (*ns*). Vehicle and hIAPP conditions showed negligible red signal (*ns*). (B,C) Different plotting styles were used in (B) and (C) to enhance data visualization: bars in (B) emphasize the relative mean differences, whereas individual data points in (C) better illustrate biological variability. Each dot represents one image; bars show mean ± SEM from at least three independent experiments. Statistical comparisons were performed using one‐way ANOVA with Dunnett's post hoc test vs. hIAPP, and Tukey's test for pairwise group comparisons. (D) Representative ICC images of INS‐1E β‐cells corresponding to the quantifications in panels B‐C. Merged channels are also shown. Images were acquired using a Leica Stellaris 8 STED super‐resolution microscope (40× oil objective).

Before performing cellular assays, we first evaluated whether fusion to the penetratin (Antp) tag alters QBP1 activity by comparing the effects of *Antp*‐QBP1 on hIAPP aggregation with those of QBP1 in ThT fluorescence assays. *Antp*‐QBP1 (1:5) strongly suppressed amyloid formation, yielding fluorescence levels close to baseline and closely mirroring the inhibitory profile of QBP1 (Figure 4A1,A2, *p* < 0.001). As expected, *Antp*‐QBP1 alone showed no ThT signal. These results indicate that fusion to the penetratin tag does not compromise QBP1 anti‐aggregation activity.

Next, to verify *Antp‐*mediated cellular uptake, we analyzed the intracellular distribution of *Antp‐*QBP1 in INS‐1E β‐cells by immunocytochemistry (ICC). Cells were treated for 48 h with exogenous hIAPP oligomers (10 µM) alone, *Antp*‐QBP1 (10 µM) alone, a combination of hIAPP oligomers and *Antp*‐QBP1 (10 µM each), or vehicle control. Co‐staining with anti‐hIAPP (FITC channel) and anti‐biotin (Red‐647 channel) antibodies allowed simultaneous evaluation of hIAPP distribution and *Antp*‐QBP1 uptake. Two complementary quantitative readouts were obtained from these images: (i) normalized intracellular fluorescence intensity per cell, reflecting peptide abundance/uptake, and (ii) the number of fluorescence spots per cell, reflecting the frequency of discrete intracellular puncta.

As shown in Figure 4B, red fluorescence intensity per cell (*a.u*.) increased markedly in cells treated with *Antp‐*QBP1 relative to hIAPP alone, consistent with effective cellular uptake. Uptake was significant whether the peptide was administered alone (*p* < 0.001) or together with hIAPP (*p* = 0.015). Interestingly, fluorescence values were slightly lower in the presence of hIAPP (∼200 *a.u*./cell) than in its absence (∼400–600 *a.u*./cell) (Table S2); this modest and non‐significant reduction is consistent with an interaction between *Antp‐*QBP1 and hIAPP, potentially involving partial sequestration of the peptide.

Similarly, Figure 4C shows that the number of intracellular fluorescent spots was significantly increased in cells treated with *Antp‐*QBP1 relative to cells treated with hIAPP alone (*p* < 0.001 for *Antp‐*QBP1 alone; *p* = 0.005 for *Antp‐*QBP1 + hIAPP). As observed for fluorescence intensity, the minor reduction during co‐treatment was not statistically significant. Both quantitative readouts displayed concordant patterns, confirming efficient intracellular delivery and sustained presence of *Antp‐*QBP1 for up to 48 h. Under all conditions, hIAPP alone did not differ from the vehicle (*ns)*, confirming that the red signal specifically corresponds to biotinylated *Antp‐*QBP1 detected by the anti‐biotin antibody. Figure 4D shows representative ICC images of INS‐1E β‐cells showing nuclei (blue), hIAPP (green), and *Antp*‐QBP1 (red), with merged channels, corresponding to the dataset quantified in Figure 4B,C.

Furthermore, to ensure that these observations were not limited to cells exposed to exogenous hIAPP, we extended the analysis to INS‐1E β‐cells stably expressing human IAPP (INS‐1E‐hIAPP) and compared them with non‐transduced control cells (INS‐1E‐Ct) under the same experimental conditions used above (*Antp‐*QBP1, 10 µM, 48 h). Under these conditions, immunostaining confirmed robust hIAPP overexpression together with efficient intracellular delivery of *Antp‐*QBP1 in INS‐1E‐hIAPP cells. These results indicate that peptide internalization is preserved even when hIAPP is produced endogenously rather than supplied exogenously. Representative images illustrating these observations are shown in Figure S8A.

Taken together, these results demonstrate efficient *Antp‐*QBP1 internalization into β‐cells, both in the absence and presence of hIAPP, without significant alteration of intracellular distribution or overall uptake.

### 3.5 Antp‐QBP1 Lowers Intracellular hIAPP Accumulation and Immunoreactivity of Amyloid Fibrils in INS‐1E β‐Cells

**FIGURE 5:**
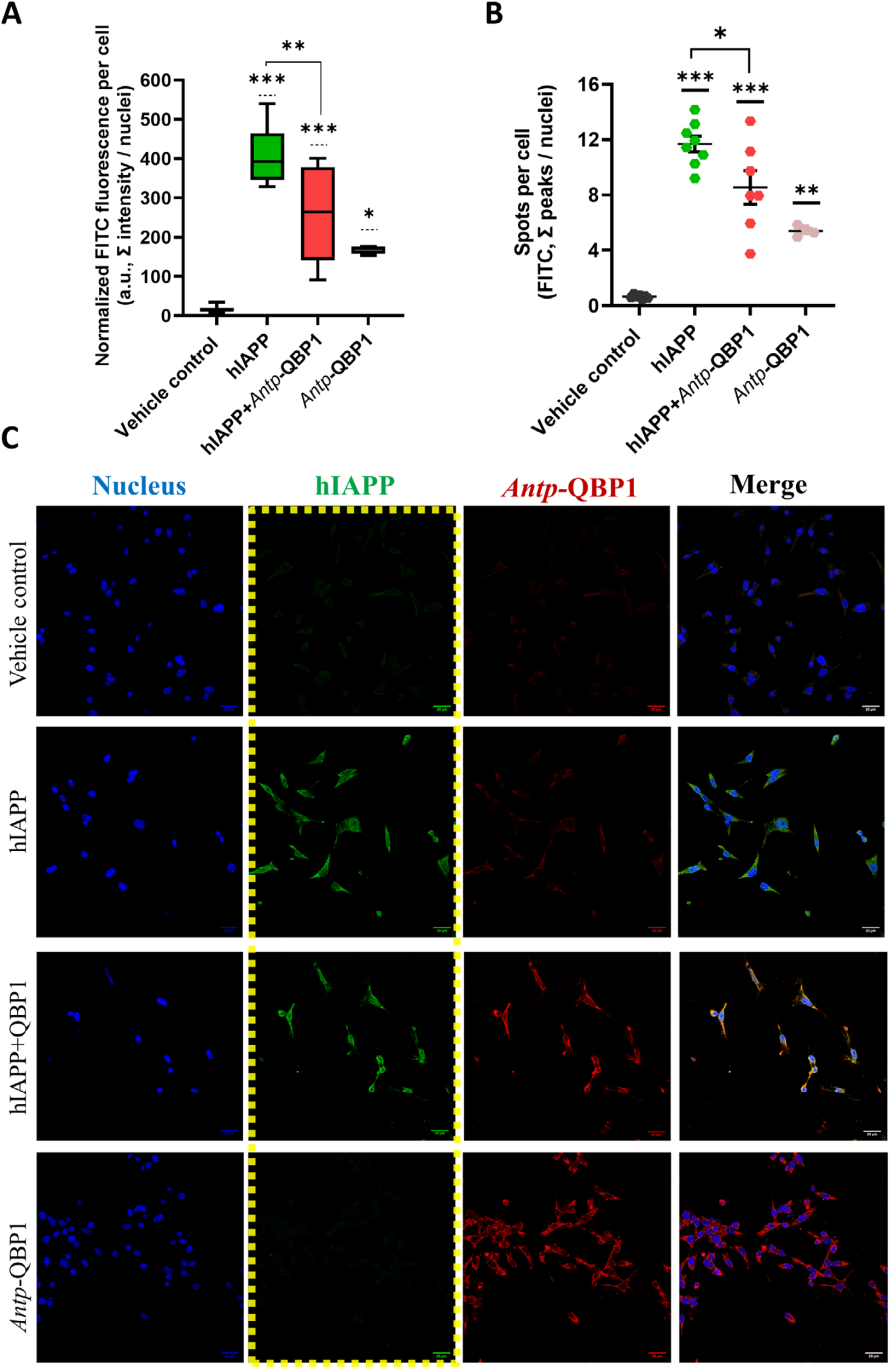
*Antp*‐QBP1 reduces intracellular hIAPP accumulation in INS‐1E β‐cells. ICC of INS‐E1 β‐cells incubated for 48 h with vehicle, hIAPP (10 µM), *Antp*‐QBP1 (10 µM), or hIAPP+*Antp*‐QBP1. Blue: nuclei (DAPI); green: hIAPP (anti‐IAPP, FITC channel); red: *Antp*‐QBP1 (anti‐biotin, Red‐647 channel). (A) Quantification of anti‐hIAPP (FITC) fluorescence intensity per cell (*Σ intensity/DAPI nuclei*). hIAPP markedly increased intracellular signal vs. vehicle (***p < 0.001). Co‐treatment with *Antp*‐QBP1 significantly reduced intensity compared with hIAPP alone (**p = 0.005). *Antp*‐QBP1 alone also yielded a low but significant signal vs. vehicle (*p = 0.016), likely reflecting antibody cross‐reactivity. (B) Quantification of hIAPP fluorescence spots per cell (*Σ peaks/DAPI nuclei*). hIAPP significantly increased the number of spots vs. vehicle (***p < 0.001), whereas co‐treatment with *Antp*‐QBP1 reduced them compared with hIAPP alone (*p = 0.024). A significant increase in spot number was also detected in *Antp*‐QBP1 alone vs. vehicle (**p = 0.002). Different plotting styles were used in (A) and (B) to enhance data visualization: bars in (A) emphasize the relative mean differences, whereas individual data points in (B) better illustrate biological variability. Each dot represents one image; bars show mean ± SEM from at least three independent experiments. Statistical comparisons were performed using one‐way ANOVA followed by Dunnett's post hoc test for comparisons vs. hIAPP, and Tukey's test where pairwise group comparisons were required. (C) Representative ICC images of INS‐1E β‐cells corresponding to the quantifications in panels A–B, including merged channels, acquired with a Leica Stellaris 8 STED microscope (40× oil).

**FIGURE 6:**
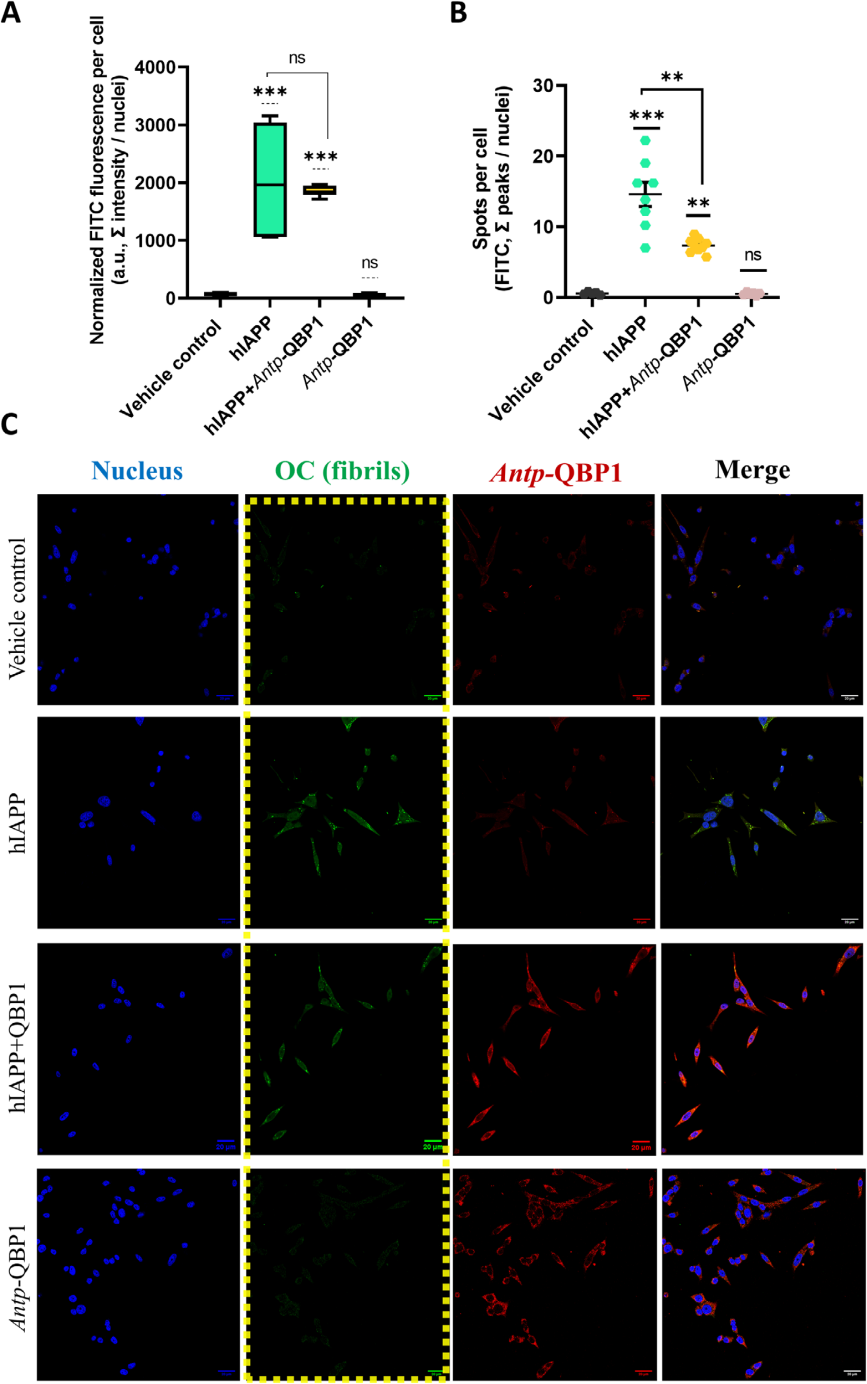
*Antp*‐QBP1 decreases the abundance of OC‐positive fibrillar spots in INS‐1E β‐cells. ICC of INS‐1E β‐cells incubated for 48 h with vehicle, hIAPP (10 µM), *Antp*‐QBP1 (10 µM), or hIAPP+*Antp*‐QBP1. Blue: nuclei (DAPI); green: mature fibrils (OC antibody, FITC); red: *Antp*‐QBP1 (anti‐biotin, Red‐647). (A) Quantification of OC (FITC) fluorescence intensity per cell (*Σ intensity/DAPI nuclei*). hIAPP markedly increased OC signal vs. vehicle (***p < 0.001). Co‐treatment with *Antp*‐QBP1 showed a non‐significant reduction compared with hIAPP alone (*ns*), although values showed reduced dispersion and lower median values. (B) Quantification of OC‐positive spots per cell (*Σ peaks/DAPI nuclei*). hIAPP significantly increased the number of fibrillar spots vs. vehicle (***p < 0.001), whereas co‐treatment with *Antp*‐QBP1 reduced spots compared with hIAPP alone (**p = 0.002). *Antp*‐QBP1 alone did not produce OC signal (*ns*). Different plotting styles were used in (A) and (B) to enhance data visualization: bars in (A) emphasize the relative mean differences, whereas individual data points in (B) better illustrate biological variability. Each dot represents one image; bars show mean ± SEM from at least three independent experiments. Statistical comparisons were performed using one‐way ANOVA with Dunnett's post hoc test vs. hIAPP, and Tukey's test for pairwise group comparisons. (C) Representative ICC images of INS‐1E β‐cells corresponding to panels A–B, including merged channels, acquired with a Leica Stellaris 8 STED microscope (40× oil).

Once its efficient uptake in β‐cells was confirmed, we then evaluated whether *Antp*‐QBP1 prevents the intracellular accumulation of hIAPP and the formation of fibrils. To do this, we quantified ICC datasets centered on the FITC channel, corresponding to hIAPP immunostaining (Figure 5) and OC‐positive fibrils (Figure 6), using the same two readouts: normalized fluorescence intensity per cell and the number of fluorescence spots per cell.

As shown in Figure 5A, hIAPP treatment markedly increased intracellular fluorescence intensity vs. vehicle (*p* < 0.001), indicating robust peptide uptake and accumulation. Co‐treatment with *Antp‐*QBP1 significantly reduced this signal (*p* = 0.005), resulting in lower intracellular hIAPP levels compared with hIAPP treatment alone. A modest but significant increase above the vehicle was also observed in cells treated with *Antp‐*QBP1 alone (*p* = 0.016), likely attributable to minor antibody cross‐reactivity, which should be considered when interpreting the magnitude of the inhibitory effect.

Consistent with these results, exposure to hIAPP significantly increased the number of intracellular spots per cell (p < 0.001), indicating a higher frequency of discrete hIAPP‐positive puncta (Figure 5B). Co‐treatment with *Antp‐*QBP1 reduced spot count compared to hIAPP alone (*p* = 0.024), resulting in fewer discrete hIAPP‐positive puncta relative to hIAPP alone. While spot values showed greater dispersion, reflecting cell‐to‐cell variability, the group treated with hIAPP alone consistently showed the highest and most uniform accumulation. A significant increase in spot number compared to the vehicle was also observed in cells treated with *Antp‐*QBP1 alone (*p* = 0.002). Representative ICC images illustrating these patterns are shown in Figure 5C.

Finally, to determine whether the intracellular hIAPP signal corresponded to fibrillar assemblies, we examined OC immunostaining under the same experimental conditions. As shown in Figure 6, treatment with hIAPP significantly increased both OC fluorescence intensity per cell and the number of OC‐positive spots vs. vehicle (*p* < 0.001), indicating the intracellular presence of OC‐reactive fibrillar species. Co‐treatment with *Antp‐*QBP1 showed a clear trend toward reduced OC intensity, with values clustering around ∼18 000 *a.u./*cell, in contrast to the broader distribution observed in hIAPP‐treated cells (∼15 000–30 000 *a.u./*cell), although this difference did not reach statistical significance (Figure 6A; and Table S2).

Conversely, *Antp‐*QBP1 co‐treatment significantly reduced the number of OC‐positive spots per cell (*p* = 0.002) (Figure 6B), without evidence of increased OC fluorescence intensity. As expected, *Antp‐*QBP1 alone produced no detectable OC signal (*ns*), confirming antibody specificity. Representative ICC images illustrating these patterns are shown in Figure 6C.

Taken together, these results indicate that *Antp*‐QBP1 reduces intracellular hIAPP accumulation in INS‐1E β‐cells. Although total OC fluorescence intensity showed only a non‐significant trend toward reduction, the number of OC‐positive spots per cell decreased significantly, indicating a lower frequency of discrete OC‐positive puncta within the analyzed time frame.

### 3.6 Antp‐QBP1 Protects INS‐1E β‐Cells from hIAPP‐Induced Cytotoxicity and Associated Transcriptional Stress Responses

**FIGURE 7:**
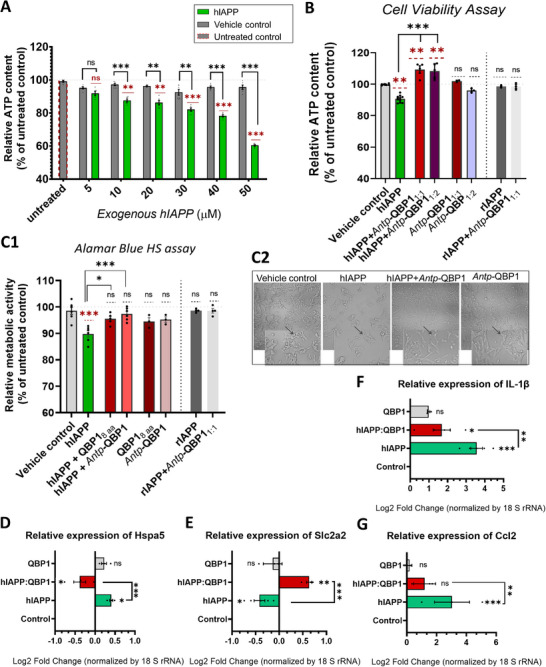
*Antp*‐QBP1 protects INS‐1E β‐cells from hIAPP‐induced cytotoxicity and associated transcriptional stress responses. (A) Dose–response analysis of intracellular ATP levels in INS‐1E β‐cells exposed to increasing concentrations of exogenous hIAPP oligomers (5–50 µM, 48 h). ATP content was quantified using the CellTiter‐Glo assay. hIAPP induced a dose‐dependent reduction in ATP levels, which became significant starting at 10 µM (**p = 0.001; ***p < 0.001 vs. untreated; red asterisks), whereas vehicle‐treated controls showed no effect. Additional pairwise comparisons between each hIAPP concentration and the corresponding vehicle control revealed significant differences at ≥10 µM (**p < 0.01; ***p < 0.001; black asterisks). (B) Intracellular ATP levels in INS‐1E β‐cells treated with hIAPP (10 µM, 48 h) in the absence or presence of *Antp‐*QBP1 (10 or 20 µM). hIAPP significantly reduced ATP levels compared with vehicle controls (**p < 0.01; red asterisks), whereas co‐treatment with *Antp‐*QBP1 restored ATP levels (both **p < 0.01; red asterisks) and significantly rescued viability relative to hIAPP alone (***p < 0.001). rIAPP alone or in combination with *Antp‐*QBP1 did not significantly alter ATP levels under the same experimental conditions, and *Antp‐*QBP1 alone also had no significant effect. (C1) Metabolic activity measured by the Alamar Blue HS assay in INS‐1E β‐cells treated with hIAPP (10 µM, 48 h) in the absence or presence of QBP1 (10 µM) or *Antp‐*QBP1 (10 µM). hIAPP significantly reduced metabolic activity compared with vehicle controls (***p < *0.001*; red asterisks), whereas both peptides prevented this reduction and restored metabolic activity to levels comparable to vehicle controls (*p = 0.015; ***p < 0.001 vs. hIAPP). In contrast, rIAPP alone or in combination with *Antp‐*QBP1 did not significantly alter metabolic activity. QBP1 and *Antp‐*QBP1 alone also had no significant effect. (C2) Representative phase‐contrast images of INS‐1E β‐cells under the indicated conditions. hIAPP treatment resulted in reduced cell density and morphological alterations, whereas co‐treatment with *Antp‐*QBP1 preserved cell morphology. Scale bars: 75 µm. (D–G) Relative mRNA expression levels of stress‐, metabolic‐, and inflammation‐related genes in INS‐1E β‐cells after 48 h treatment with hIAPP (10 µM), *Antp‐*QBP1 (10 µM), or their combination. Gene expression was normalized to 18S rRNA and expressed relative to vehicle controls. (D) *Hspa5* (BiP/GRP78) expression. Exogenous hIAPP significantly upregulated Hspa5 (*p = *0.027*), indicating activation of an ER stress–associated transcriptional response. Co‐treatment with *Antp‐*QBP1 significantly reduced *Hspa5* expression (***p < 0.001 vs. hIAPP; *p = 0.036 vs. control). (E) *Slc2a2* (GLUT2) expression. hIAPP treatment significantly downregulated Slc2a2 (*p = *0.024*), consistent with metabolic dysregulation, whereas *Antp‐*QBP1 co‐treatment significantly increased *Slc2a2* expression (***p < 0.001 vs. hIAPP; **p = 0.002 vs. control), restoring and slightly exceeding basal levels. (F) *IL‐1β* expression. hIAPP significantly increased *IL‐1β* levels (***p < 0.001), an effect that was significantly reduced upon *Antp‐*QBP1 co‐treatment (**p = 0.007 vs. hIAPP). (G) *Ccl2* expression. hIAPP treatment strongly induced Ccl2 (***p < 0.001), whereas *Antp‐*QBP1 co‐treatment significantly attenuated this response (**p = 0.002 vs. hIAPP). *Antp‐*QBP1 alone did not significantly differ from vehicle‐treated controls for any of the genes analyzed. Data represent mean ± SEM from at least three independent experiments. Statistical analysis was performed using one‐way ANOVA with Dunnett's post hoc test against the appropriate control and Tukey's test for pairwise comparisons. Statistical significance is indicated as *p < 0.05, **p < 0.01, ***p < 0.001; ns, not significant.

To evaluate whether *Antp*‐QBP1 protects INS‐1E β‐cells from hIAPP‐induced cytotoxicity, cells were exposed to exogenous hIAPP oligomers for 48 h after overnight culture in high‐glucose medium (16.7 mm), mimicking T2D‐like hyperglycemic conditions. hIAPP preparations were pre‐incubated, verified as A11‐positive, and sonicated prior to addition to ensure the presence of prefibrillar assemblies. This experimental setting enabled systematic evaluation of ATP content, cell morphology, metabolic activity, and transcriptional responses under hIAPP‐induced stress (Figure 7).

We first determined the minimal concentration required to induce measurable cytotoxicity by exposing INS‐1E β‐cells to increasing concentrations of exogenous hIAPP oligomers (5–50 µM, 48 h), followed by quantification of intracellular ATP levels (Figure 7A). Consistent with the expected cytotoxic profile of prefibrillar hIAPP assemblies, exposure produced a clear dose‐dependent decrease in intracellular ATP. A slight, non‐significant decline was detected at 5 µM, indicating a subthreshold concentration for measurable metabolic impairment. In contrast, significant reductions emerged at 10 µM (*p* = 0.001) and intensified at higher doses, reaching *p* < 0.001 at 30–50 µM. Vehicle‐treated controls showed no change, confirming the specificity of the toxic effect. These data identify 10 µM as the minimal effective concentration required to induce hIAPP‐driven cytotoxicity, and this condition was therefore selected to evaluate whether *Antp*‐QBP1 can prevent β‐cell damage during early oligomer formation.

To specifically target early stages of aggregation, *Antp*‐QBP1 was pre‐incubated with hIAPP and subsequently co‐administered to INS‐1E β‐cells. As expected, hIAPP alone (10 µM, 48 h) significantly reduced ATP levels compared with the vehicle condition (*p* < 0.01) (Figure 7B). In contrast, co‐treatment with *Antp*‐QBP1 led to a robust recovery of intracellular ATP, with significant improvements observed at both 10 µM and 20 µM (both *p* < 0.01). Direct comparison with hIAPP‐treated cells confirmed a highly significant recovery (*p* < 0.001), with ATP levels increasing by ∼19% and slightly exceeding those observed in the vehicle control condition. Notably, increasing the *Antp*‐QBP1 concentration from 10 to 20 µM did not produce additional gains, suggesting a plateau effect at higher doses.

Consistent with its non‐amyloidogenic nature, rIAPP did not reduce ATP levels, either alone or in combination with *Antp‐*QBP1, confirming the absence of cytotoxic effects under the same experimental conditions. Similarly, *Antp‐*QBP1 alone did not alter ATP levels, supporting a selective protective effect under hIAPP‐induced stress.

To complement the ATP‐based viability assays with an independent metabolic readout, we employed the Alamar Blue HS assay under identical treatment conditions (Figure 7C). In these experiments, we also included the QBP1 octapeptide, whose co‐incubation with hIAPP allows sufficient time to interfere with oligomer formation prior to cellular exposure. As expected, hIAPP alone (10 µM, 48 h) significantly reduced metabolic activity compared with vehicle‐treated controls (*p* < 0.001), confirming its cytotoxic effect. In contrast, co‐treatment with either QBP1 or *Antp‐*QBP1 (10 µM each; hIAPP: inhibitor, 1:1) completely prevented this reduction and restored metabolic activity to control levels (*p* = 0.015 and *p* < 0.001 vs. hIAPP, respectively) (Figure 7C1), consistent with their in vitro anti‐aggregation activity. *Antp‐*QBP1 exhibited a modestly stronger protective effect than QBP1, consistent with its additional intracellular delivery via the penetratin tag.

Phase‐contrast microscopy at 48 h corroborated these findings (Figure 7C2), showing a marked reduction in cell density and morphological deterioration following hIAPP exposure, whereas co‐treatment with Antp‐QBP1 preserved cell morphology and partially restored cell density toward control levels.

In agreement with the ATP measurements, rIAPP did not affect metabolic activity, either alone or in combination with *Antp‐*QBP1. Unlike the ATP assay, neither peptide increased metabolic activity above baseline in the Alamar Blue assay, suggesting that the increase observed in ATP measurements may reflect assay‐dependent effects [48, 81]. Overall, both peptides attenuated the hIAPP‐associated reduction in metabolic activity and restored values to baseline levels.

We next examined whether the protective effect of *Antp‐*QBP1 was accompanied by transcriptional changes in stress‐ and metabolism‐related pathways by analyzing the expression of selected β‐cell genes (*Hspa5*, *Slc2a2*, *IL‐1β*, and *Ccl2*) by RT‐qPCR. Slc2a2 (Solute carrier family 2, member 2; GLUT2) is a key β‐cell glucose transporter required for glucose sensing and insulin secretion; Hspa5 (Heat shock protein family A [Hsp70] member 5, BiP/GRP78) encodes an endoplasmic reticulum (ER)‐resident chaperone central to proteostasis and the unfolded protein response (UPR); and IL‐1β (interleukin‐1β) and Ccl2 (C–C motif chemokine ligand 2, MCP‐1) encode inflammatory mediators linked to cytokine‐induced β‐cell stress and dysfunction [8, 82, 83]. To this end, gene expression was normalized to 18S rRNA, and relative changes were compared across control (vehicle), hIAPP, *Antp‐*QBP1, and hIAPP + *Antp‐*QBP1 conditions (Figure 7D–G; and Table S1).

Exposure to hIAPP (10 µM, 48 h) led to transcriptional alterations consistent with proteotoxic and inflammatory stress. *Hspa5* expression was significantly upregulated following hIAPP treatment (*p* = 0.027) (Figure 7D), indicating activation of an ER stress‐associated transcriptional response consistent with UPR activation [84, 85, 86]. In contrast, co‐treatment with *Antp‐*QBP1 (1:1) significantly decreased *Hspa5* expression compared with hIAPP alone (*p* < 0.001), and levels were even lower than those observed in control cells (*p* = 0.036). This result indicates not only attenuation of the hIAPP‐associated increase but also a reduction below basal condition. Given that cells were maintained under high‐glucose culture conditions, which impose a degree of baseline metabolic stress, this decrease may reflect a broader normalization of stress‐associated transcriptional activity. *Antp‐*QBP1 alone did not significantly affect *Hspa5* expression relative to control cells, indicating that its transcriptional effect is primarily observed under hIAPP‐induced stress.

In parallel, *Slc2a2* expression was significantly downregulated by hIAPP exposure (*p* = 0.024) (Figure 7E), consistent with reduced expression of the GLUT2 glucose transporter in β‐cells under amyloidogenic stress [87, 88]. In contrast, co‐treatment with *Antp‐*QBP1 significantly upregulated *Slc2a2* expression (*p* < 0.001 vs. hIAPP; *p* = 0.002 vs. control), restoring and even exceeding basal expression levels. Given the central role of GLUT2 in glucose sensing and β‐cell metabolic function, these data indicate that *Antp‐*QBP1 counteracts the hIAPP‐associated transcriptional downregulation of this key metabolic gene.

Finally, both *IL‐1β* and *Ccl2* were strongly induced by hIAPP treatment (*p* < 0.001 vs. control; Figure 7F,G), indicating activation of inflammatory transcriptional responses under amyloidogenic stress [10, 51, 82, 89]. Co‐treatment with *Antp‐*QBP1 significantly reduced the expression of both genes (*p* = 0.007 for *IL‐1β*, *p* = 0.002 for *Ccl2* vs. hIAPP), consistent with attenuation of the pro‐inflammatory response. Importantly, *Antp‐*QBP1 alone did not significantly alter the expression of these transcripts relative to control, indicating that its transcriptional impact is primarily evident in the context of hIAPP‐induced stress.

Together, these findings indicate that *Antp‐*QBP1 protects INS‐1E β‐cells from hIAPP‐induced cytotoxicity while attenuating associated ER stress, inflammatory signaling, and metabolic dysfunction. However, the functional implications of these transcriptional changes warrant further investigation.

In addition, we utilized the hIAPP‐overexpressing INS‐1E model (INS‐1E‐hIAPP) as a complementary system to evaluate QBP1 cellular uptake and its activity under conditions of sustained intracellular peptide production. hIAPP overexpression significantly reduced intracellular ATP levels compared with control cells (*p* < 0.001), confirming that sustained intracellular hIAPP expression compromises β‐cell viability [90, 91]. In contrast, *Antp*‐QBP1 treatment (5–20 µM, 48 h) restored ATP levels across all concentrations tested, with a modest but significant effect at 5 µM (*p* < 0.05), a robust rescue at 10 µM (*p* < 0.001), and sustained improvement at 20 µM (*p* < 0.05) compared with untreated INS‐1E‐hIAPP cells (Figure S8B1). *Antp*‐QBP1 did not alter ATP levels in control (Ct) cells, supporting a selective protective effect under hIAPP overexpression–induced stress.

Direct comparison of INS‐1E‐hIAPP and Ct cells exposed to identical *Antp*‐QBP1 concentrations revealed significant differences under all conditions, with 10 µM producing the most pronounced effect: at this dose, ATP levels in INS‐1E‐hIAPP cells were not only restored to control levels but modestly exceeded them (*p* < 0.001). Phase‐contrast microscopy supported these findings (Figure S8B2), showing morphological alterations, including cell rounding and partial detachment, in INS‐1E‐hIAPP cells, whereas *Antp*‐QBP1 treatment preserved a morphology comparable to that of control cells. Together, these observations are consistent with the protective effects observed in the exogenous hIAPP oligomer model, supporting the ability of *Antp*‐QBP1 to mitigate both extracellular and intracellular hIAPP‐associated toxicity.

### 3.7 Aromatic Interactions Drive the Binding of QBP1 to the hIAPP Amyloid Core

To investigate the molecular determinants underlying the interaction between **amylin** (**PDB ID: 2L86** [65]; represented in Figure S1A) and the inhibitory peptides QBP1, SC‐M11, and SC‐M8 [38, 45], we performed computational modelling using molecular docking followed by MD simulations.

**FIGURE 8:**
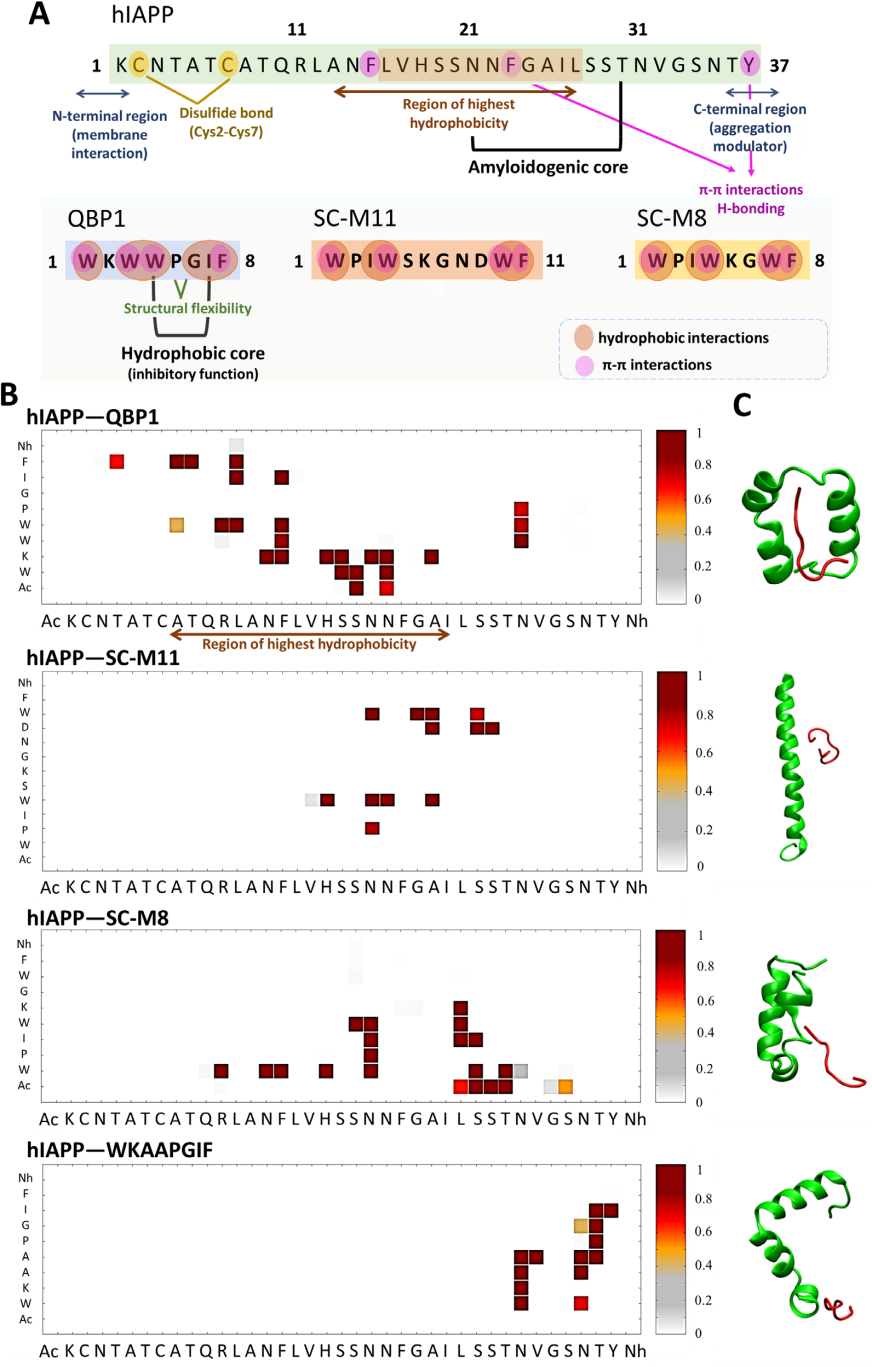
Binding modes and contact interactions between hIAPP and QBP1‐based variants (QBP1, SC‐M11, SC‐M8, WKAAPGIF). (A) Schematics of primary structural and physicochemical features of hIAPP and the inhibitory peptides. The hydrophobic core of hIAPP, key aromatic residues, and regions involved in intermolecular interactions are highlighted, illustrating their potential role in self‐aggregation and inhibition. The inhibitory peptides are depicted with their respective hydrophobic and flexible regions, which may influence their binding to hIAPP. (B) Contact‐probability maps across MD trajectories. From top to bottom: hIAPP–QBP1, hIAPP–SC‐M11, hIAPP–SC‐M8 and hIAPP–WKAAPGIF. The color scale (0‐1) encodes the probability of residue–residue contact (red = high, gray = low). One‐letter amino acid codes are used; *Ac* and *Nh* denote the N‐ and C‐terminal capping groups, respectively. *C* indicates the cysteine residues forming the Cys2–Cys7 disulphide bond, which may contribute to structural stability. QBP1 concentrates contacts over the hydrophobic amyloidogenic core (residues ∼22–29), whereas SC variants display attenuated or fragmented interactions; the WKAAPGIF mutant shifts its engagement toward the distal C‐terminal region, showing reduced occupancy within the core. (C) Representative complex conformations highlighting the predominant contact regions (hIAPP in green; peptides in red). QBP1 adopts a compact, well‐aligned pose with high shape complementarity; SC‐M11/SC‐M8 show reduced complementarity; WKAAPGIF exhibits a terminal “end‐capping” configuration, tethered at the distal C‐terminus rather than embracing the amyloidogenic core.

Figure 8A summarizes the main structural and physicochemical features associated with the hIAPP sequence and the three peptides. hIAPP contains a highly hydrophobic central region (∼residues 15–26), a segment repeatedly associated with aggregation propensity and fibril formation [11, 13, 17], providing a plausible hotspot for peptide binding. The C‐terminal segment also contributes to aggregation modulation through its enrichment in aromatic and polar residues, which mediate intermolecular packing and β‐sheet alignment. Notably, Tyr37 can engage in *π*–*π* stacking and hydrogen‐bonding interactions that influence early oligomerization and fibril nucleation, although its role in fibril stabilization remains a subject of debate [92, 93].

QBP1 has a compact hydrophobic core composed of Trp (W), Phe (F), and Pro (P) residues [45], which likely contributes to its interaction with the hydrophobic domains of hIAPP. The presence of Gly (G) confers structural flexibility, whereas Pro residues may disfavor β‐sheet propagation [94, 95]. In contrast, SC‐M11 and SC‐M8 have their hydrophobic and aromatic residues rearranged, which may compromise their ability to form stable *π*–*π* stacking and hydrogen‐bonding interactions with hIAPP, thus reducing their inhibitory capacity [45].

To better understand the molecular basis of amylin recognition, we analyzed the residue–residue contact probability maps and the binding‐energy decompositions per‐residue obtained from the MD trajectories (Figure 8; Figures S9–S12). QBP1 concentrated high‐probability contacts over the region of greatest hydrophobicity, covering the hIAPP amyloidogenic core (∼22–29; NNFGAILS) and extending toward the preceding hydrophobic segment (around F15–L16), with only minor occupation at the distal C‐terminus (Figure 8B; Figure S9). The interaction pattern reflected aromatic/hydrophobic pairing, consistent with a *π*–*π*‐driven interface where W/F residues of QBP1 engage the core and mask aggregation‐prone motifs. Additional contacts near the Cys2–Cys7 disulfide loop contributed to a more defined interaction surface, supporting a mechanism in which QBP1 stabilizes native conformations rather than disrupting amylin non‐specifically (Figures S10A,S11A). Consistently, QBP1 showed the most favorable binding free energy (ΔG_bind_ = –5.70 kcal·mol^−^^1^; Table 1) [96, 97] and adopted a compact, well‐aligned conformation with high shape complementarity to hIAPP (Figure 8C).

**TABLE 1:** MM/PBSA binding‐energy components for hIAPP complexes with QBP1, SC‐M11, SC‐M8, and the Trp‐depleted mutant WKAAPGIF. Reported values (kcal.mol^−^^1^) are van der Waals interactions (*ΔE_vdW_*), reflecting non‐covalent attractive forces; electrostatic interactions (*ΔE_ele_*), capturing charge‐charge interactions; polar solvation (*ΔG_PB_*) and non‐polar solvation (*ΔG_NP_*), representing solvent effects on binding; entropy (−*TΔS*); total binding free energy (*ΔG_bind_*), and enthalpy (*ΔH* = *ΔE_vdW_* + *ΔE_ele_* + *ΔG_PB_* + *ΔG_NP_*). More negative values indicate stronger stabilizing contributions; *ΔG_bind_* = *ΔH* − *TΔS*. QBP1 shows the most favorable *ΔG_bind_* (−5.704), SC‐M11 and SC‐M8 are less favorable (−3.793 and −3.520), and WKAAPGIF is unfavorable (+4.563), consistent with the loss of Trp‐driven aromatic stabilization. The conformations corresponding to these energy calculations are shown in Figure 8C.

	ΔE_vdW_	ΔE_ele_	ΔG_PB_	ΔG_NP_	−TΔS	ΔG_bind_	Enthalpy
**QBP1**	−55.487	2.718	23.464	−6.205	29.840	**—5.704**	−35.544
**SC‐M11**	−31.271	−0.294	12.673	−3.068	18.180	**—3.793**	−21.982
**SC‐M8**	−39.640	−1.515	15.457	−3.997	26.175	**—3.520**	−29.713
**WKAAPGIF**	−21.960	−1.753	8.615	−3.029	22.728	**+4.563**	−18.165

Conversely, SC‐M11 and SC‐M8 exhibited weaker and lower‐probability interactions, indicating suboptimal binding motifs. SC‐M11 showed limited occupation of the hydrophobic core, while SC‐M8 maintained only partial contact within the 20–29 region (Figure 8B; Figure S9). These binding patterns are consistent with their lower inhibitory activity observed experimentally and suggest that the rearrangement of aromatic residues may compromise the *π*–*π* stacking and hydrogen‐bonding geometry compatible with stable binding. Energy decomposition per‐residue revealed attenuated and more fragmented stabilizing contributions, including weaker Trp‐mediated interactions (Figures S10B,C and S11B,C), and MM/PBSA yielded less favorable ΔG_bind_ values (–3.79 and –3.52 kcal·mol^−^^1^ for SC‐M11 and SC‐M8, respectively; Table 1). Structurally, both SC peptides exhibited reduced shape complementarity with respect to QBP1 (Figure 8C).

To further investigate the role of aromatic density, we computationally designed a Trp‐to‐Ala mutant of QBP1 (WKAAPGIF). MD simulations revealed attenuated contact density over the 20–29 core, a shift of engagement toward the C‐terminal segment, and a marked reduction in stabilizing contributions per‐residue (Figure 8B; Figure S9). The loss of Trp flattened the energy landscape (fewer strong contributors and more uniformly weak stabilization) consistent with diminished *π*–*π* interactions and weaker overall binding profiles (Figures S10D,S11D). Accordingly, MM/PBSA yielded an unfavorable binding free energy (ΔG_bind_ = +4.563 kcal·mol^−^^1^; Table 1), supporting a marked reduction in binding propensity upon loss of aromatic residues. The mutant adopted an “end‐capping” configuration, interacting predominantly with the distal terminus rather than docking to the amyloidogenic core (Figure 8C).

Furthermore, in order to obtain a quantitative view of the interaction energetics we performed binding free‐energy decomposition (Table 1; and Figures S11). In the hIAPP–QBP1 complex, van der Waals interactions constituted the dominant stabilizing component (ΔE_vdW_ = −55.49 kcal·mol^−^^1^), while electrostatics contributed only marginally (ΔE_ele_ = +2.72). Although polar solvation opposed binding (ΔG_PB_ = +23.46), this desolvation cost was partly offset by nonpolar solvation (ΔG_NP_ = −6.21) along with the strong van der Waals term, resulting in the most favorable enthalpy (ΔH = −35.54) and ΔG_bind_ among the peptides examined. In the case of the SC variants, van der Waals stabilization was markedly weaker (SC‐M8: ΔE_vdW_ = −39.64, SC‐M11: −31.27), and both showed modest electrostatic contributions (ΔE_ele_ = −1.52/−0.29). These differences resulted in less favorable enthalpies (ΔH = −29.71/−21.98) and correspondingly less negative ΔG_bind_ values. Interestingly, SC‐M11 showed slightly more favorable energetics than SC‐M8 despite its weaker ΔE_vdW_ term, due to a lower desolvation penalty (ΔG_PB_ = +12.67 *vs* +15.46) and a reduced entropic cost (−TΔS = +18.18 *vs* +26.18). The Trp‐depleted mutant showed the weakest van der Waals stabilization (ΔE_vdW_ = −21.96) and an overall shallow stabilization profile, resulting in a modest enthalpy (ΔH = −18.17) and an unfavorable ΔG_bind_.

Consistent with these energetic trends, QBP1 established a broader network of aromatic contacts involving several Trp and Phe residues (particularly W3 and W4; Figure S12A). In contrast, the SC‐M11 and SC‐M8 interactions collapsed into a single dominant site (mainly W4) with minimal contribution from other aromatic side chains (Figure S12B–C), while the Trp‐depleted mutant maintained only one weak contact (Figure S12D). This progressive reduction in aromatic‐contact density parallels the ΔE_vdW_ ranking (QBP1< SC‐M8< SC‐M11< WKAAPGIF; the more negative, the stronger) and highlights Trp‐mediated van der Waals packing as a major contributor to binding affinity. The balance between strong nonpolar stabilization and limited electrostatic contribution points to a binding mechanism primarily governed by hydrophobic collapse rather than charge complementarity. The markedly more negative ΔG_bind_ of QBP1 thus reflects an optimized packing of hydrophobic and aromatic residues at the QBP1–amylin interface, stabilizing a compact complex resistant to amyloid conversion. Conversely, the lower hydrophobic contribution and weaker interfacial cohesion observed in SC‐M11 and SC‐M8 are expected to impair their ability to efficiently engage and stabilize early hIAPP assemblies, consistent with their experimentally observed lower inhibitory capacity.

**FIGURE 9:**
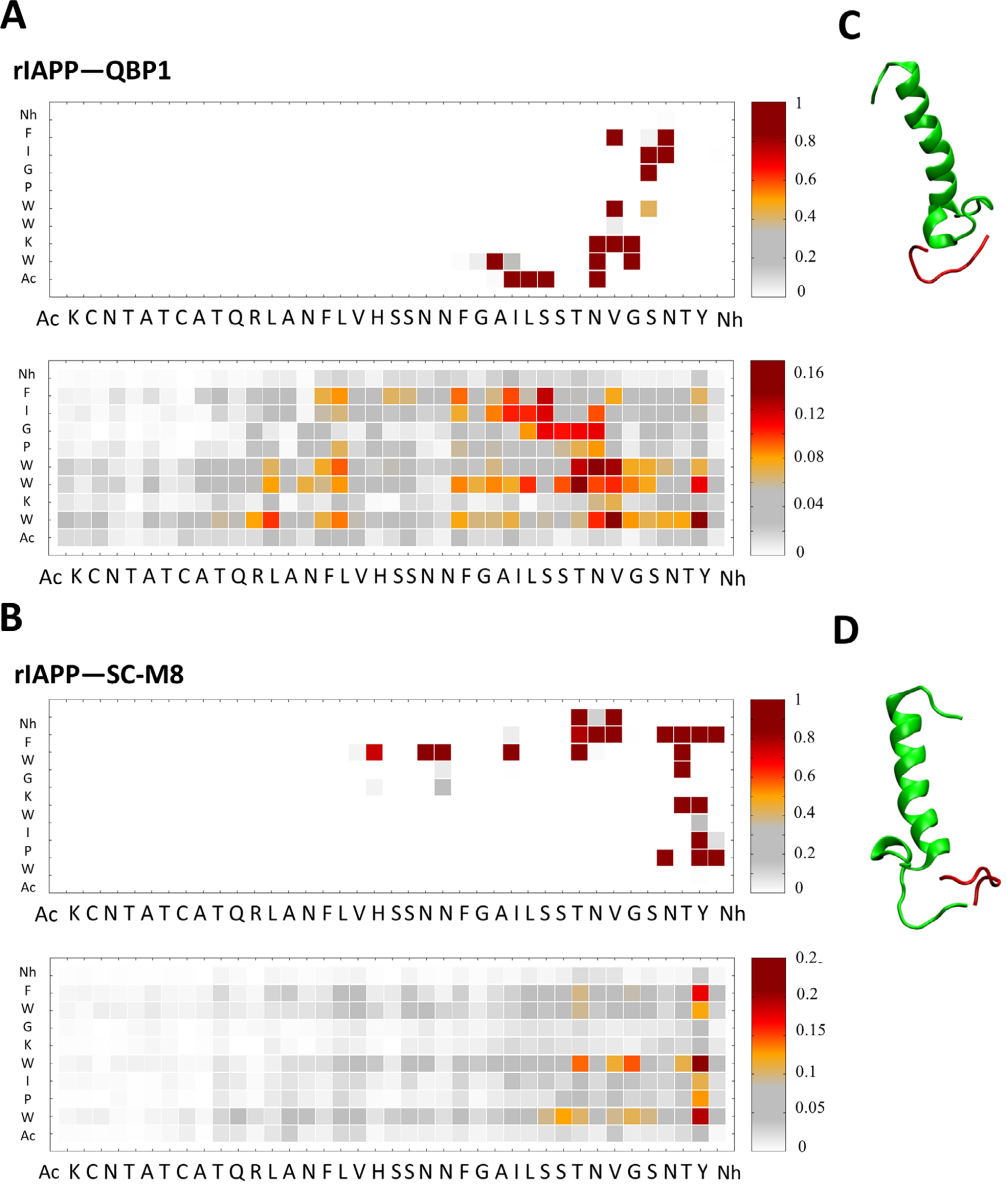
Binding modes and contact interactions between non‐amyloidogenic rIAPP with QBP1 and SC‐M8. Contact‐probability maps and representative conformations derived from molecular dynamics (MD) simulations of rIAPP–peptide complexes. The color scale (0–1) represents the probability of residue–residue contact (red, high probability; gray, low probability). One‐letter amino acid codes are used; Ac and Nh denote N‐ and C‐terminal capping groups, respectively. (A) rIAPP–QBP1 complex. Top panel: representative contact‐probability map. Bottom panel: averaged contact probabilities across all sampled conformations. (B) rIAPP–SC‐M8 complex. Top panel: representative contact‐probability map. Bottom panel: averaged contact probabilities across all sampled conformations. (C) Representative conformation of the rIAPP–QBP1 complex, highlighting predominant interaction sites (rIAPP shown in green; peptide in red). (D) Representative conformation of the rIAPP–SC‐M8 complex, highlighting predominant interaction sites (rIAPP shown in green; peptide in red). In both complexes, QBP1 and SC‐M8 interact preferentially with the C‐terminal region of rIAPP. In contrast to hIAPP complexes, neither peptide forms a compact, well‐aligned interface with the amyloid core, but instead adopts a terminal “end‐capping” binding mode at the distal end of the helix, consistent with the loss of amyloid‐core recognition in non‐amyloidogenic rIAPP.

Finally, to assess the amyloid specificity of QBP1 binding to hIAPP, we performed equivalent simulations using the non‐amyloidogenic **rIAPP** (sequence: KCNTATCATQRLANFLVRSSNNLGPVLPPTNVGSNTY) (**PDB: 2KJ7** [66]; Figure S1B), which contains key substitutions (H18R, F23L, A25P, S28P, S29P, and I33V) known to disrupt β‐sheet formation [98]. Unlike hIAPP, where QBP1 engages an extended amyloidogenic core, both rIAPP–QBP1 and rIAPP–SC‐M8 (Figure 9A,B) complexes exhibited localized, asymmetric interaction patterns predominantly localized to the C‐terminal region. Although dense contact zones were detected in the averaged maps, these interactions remained confined to this distal segment and did not extend over the structured core (Figure 9C,D), indicating reduced cooperativity and a loss of the hydrophobic clustering characteristic of hIAPP complexes.

Energetically, the rIAPP–QBP1 complex showed weaker stabilization (ΔE_vdW_ = –33.5 kcal·mol^−1^; ΔG_bind_ = –3.54 kcal·mol^−1^), dominated by nonspecific van der Waals contributions with minimal electrostatic or solvation compensation. Although the rIAPP–SC‐M8 complex displayed a comparatively favorable van der Waals contribution (ΔE_vdW_ = –48.2 kcal·mol^−1^), this interaction was accompanied by a large positive electrostatic term (ΔE_ele_ = +32.1 kcal·mol^−1^) and an unfavorable solvation profile, consistent with a poorly oriented and transient interface (Table 2; and Figure S13). Consistent with the contact maps, SC‐M8 interactions with rIAPP remained confined to the distal C‐terminal region rather than engaging the amyloidogenic core. Accordingly, the apparently favorable binding of SC‐M8 to the non‐amyloidogenic isoform likely reflects nonspecific and unproductive surface adhesion rather than the formation of a compact or structurally meaningful complex. Given the intrinsically non‐amyloidogenic nature of rIAPP, such interactions—despite their apparent energetic favorability—are unlikely to interfere with aggregation‐prone regions, indicating that binding affinity alone may not fully account for anti‐amyloid activity.

**TABLE 2:** Binding energy components of rIAPP–QBP1 and rIAPP–SC‐M8 complexes. The table summarizes the energetic contributions to the total binding free energy (Δ*G_bind_*) for the rat amylin (rIAPP) complexes with QBP1 and SC‐M8, as derived from MD simulations. The components include: van der Waals interactions (Δ*E_vdW_*), reflecting non‐covalent attractive forces; electrostatic interactions (Δ*E_ele_*), capturing charge–Charge interactions; polar solvation energy (Δ*G_PB_*) and non‐polar solvation energy (Δ*G_NP_*), representing solvent effects on binding. The total binding free energy (ΔG__bind_) and enthalpy are also reported, with enthalpy representing the binding energy excluding the entropic contribution. All values are expressed in kcal·mol^−^^1^, where more negative values denote stronger stabilizing effects.

	ΔE_vdW_	ΔE_ele_	ΔG_PB_	ΔG_NP_	−TΔS	ΔG_bind_	Enthalpy
**QBP1**	−33.526	−6.524	17.893	−4.618	23.269	—**3.540**	−26.809
**SC‐M8**	−48.213	32.067	−11.359	−6.099	18.807	—**14.835**	−33.642

Collectively, these computational analyses indicate that aromatic density and van der Waals stabilization contribute to QBP1 binding to the hIAPP amyloid core, consistent with its experimentally observed anti‐aggregation activity.

## 4 Discussion

Given that hIAPP aggregation is established as a pivotal driver of β‐cell dysfunction and depletion in T2D [6, 7], disrupting its amyloidogenic pathway has emerged as a prime therapeutic strategy [99, 100, 101]. However, despite extensive efforts, only a limited number of aggregation inhibitors have shown translational success [102, 103]. Among these, pramlintide reduces amyloidogenicity [15] but is limited by formulation and delivery constraints [29]. In contrast, next‐generation amylin analogues such as davalintide, cagrilintide, and DACRAs have been developed to improve metabolic efficacy [30, 31], yet they do not directly target hIAPP aggregation. These limitations highlight the need for strategies that selectively modulate early aggregation events.

In this work, we have demonstrated that the anti‐amyloidogenic peptide QBP1, specifically its minimal active core [32, 47], delays early hIAPP amyloidogenesis and reduces hIAPP‐associated β‐cell stress while preserving cell viability in vitro. CD spectroscopic data show that QBP1 arrests the coil‐to‐β‐sheet transition, while solution NMR indicates a delay in the formation of early aggregated hIAPP species, as inferred from reduced signal loss over time. These observations are consistent with an MD‐derived model in which QBP1 interacts with hIAPP through Trp‐mediated hydrophobic and π–H interactions. By preferentially targeting early aggregation intermediates, QBP1 limits the build‐up of aggregation‐prone assemblies, thereby reducing cellular stress and preserving β‐cell viability [41, 45, 46]. Figure S14 summarizes how each experimental approach (CD, NMR, MD, ThT, A11/OC, microscopy) relates to specific aggregation stages and illustrates QBP1 involvement in this pathway.

This mechanism aligns with previous evidence showing that QBP1 targets early conformational transitions in various amyloidogenic proteins [36, 37, 38, 39, 40, 42]. Our findings now extend this protecting property to islet amyloidosis in T2D. Other inhibitors, such as resveratrol and insulin, act similarly at early aggregation stages: resveratrol binds histidine (H18) to prevent oligomer formation, while insulin interacts with H18 and Y37 under acidic conditions to stabilize hIAPP [104, 105]. Likewise, EGCG binds early to hIAPP, disrupting inter‐ and intra‐peptide β‐sheet contacts and redirecting aggregation toward off‐pathway, amorphous species [106, 107, 108]. Together, these findings reinforce the relevance of upstream intervention strategies in hIAPP‐driven β‐cell stress and position QBP1 as a promising lead compound for slowing amyloid formation in pancreatic islets.

Consistently, using complementary biophysical and imaging approaches, we found that QBP1 at a five‐fold molar excess inhibits hIAPP amyloidogenesis in vitro by delaying fibril nucleation and elongation [21, 77, 109]. ThT kinetics, combined with A11 [53] and OC [54] immunoreactivity assays, revealed a substantial reduction in both oligomeric and fibrillar species, with TEM imaging showing a scarcity of mature fibrils and a predominance of amorphous aggregates upon incubation [110]. These findings, in alignment with CD and NMR data, suggest that QBP1 effectively impairs β‐sheet transition and the initial stages of oligomerization. Although fibril formation is not completely abolished—similar to other short peptide inhibitors such as SNNFGA and GAILSS [22, 111]—QBP1 appears to destabilize early nuclei and limit the build‐up of cytotoxic species [34, 35, 41].

In addition to its anti‐amyloidogenic activity in vitro, QBP1 preserved the viability of INS‐1E pancreatic β‐cells exposed to hIAPP [2, 14, 59] and reduced intracellular peptide accumulation and fibrillar load, suggesting that it may also limit amyloid formation in the cellular environment [56, 57]. These protective effects were associated with its efficient intracellular delivery using the penetratin tag (*Antp*‐QBP1) [38, 40], which retained full inhibitory activity and showed no cytotoxicity up to 100 µM [36, 38, 112, 113]. Compared with other delivery platforms—such as viral vectors or liposomes—this PTD system offers high in vivo efficiency, low toxicity, and controllable dosing [33, 38].

To examine the effects of *Antp*‐QBP1 under controlled amyloidogenic conditions, we employed a cellular model based on the application of exogenous preformed hIAPP aggregates to INS‐1E β‐cells [10, 59], allowing precise control over peptide concentration and aggregation state [2, 14]. Within this framework, A11‐positive oligomeric species increased with hIAPP concentration and correlated with reduced β‐cell viability, with 10 µM identified as the minimal reproducible toxic dose [10, 51, 102]. To further promote amyloidogenic stress, cells were cultured under high‐glucose conditions (16.7 mM), known to enhance amyloid formation and exacerbate hIAPP‐associated toxicity [8, 58]. Under these defined conditions, *Antp*‐QBP1 was found to preserve β‐cell viability at equimolar and moderate excess concentrations, with no additional benefit at higher doses, suggesting a saturable interaction or limited availability [114, 115].

Consistent with this, ICC analyses showed a marked reduction in intracellular hIAPP oligomer accumulation upon *Antp*‐QBP1 treatment, suggesting interference with oligomer formation and/or cellular uptake. In agreement with this observation, OC staining revealed fewer β‐sheet–rich structures despite comparable overall signal intensity, supporting a mechanism in which QBP1 primarily limits early nucleation and seeding, and does not appear to interfere with the formation of mature fibrils. Given that hIAPP oligomers exert toxicity through membrane interactions [116, 117] and propagate via uptake‐dependent mechanisms [118, 119], the reduced intracellular signal suggests that *Antp‐*QBP1 may also limit membrane‐associated toxicity and cellular uptake of these assemblies [120, 121]. However, residual assemblies may continue to mature into stable β‐sheet aggregates, suggesting that sustained QBP1 availability could be required for long‐term suppression of amyloid build‐up.

The inclusion of the non‐amyloidogenic peptide rIAPP as a control [2, 14] further supports the specificity of QBP1. As expected, rIAPP did not generate A11‐reactive oligomeric species, OC‐positive fibrillar structures, or cytotoxic effects under our experimental conditions. These results confirm that the protective activity of QBP1 is strictly linked to the mechanistic specificity of hIAPP aggregation rather than non‐specific peptide interactions.

An exploratory targeted gene expression analysis revealed that hIAPP exposure induced ER stress and inflammatory responses, as reflected by increased *Hspa5, Il1b*, and *Ccl2* expression and reduced *Slc2a2* levels. These genes were selected based on previous studies demonstrating their sensitivity to hIAPP‐induced proteotoxic and inflammatory stress in β‐cells [8, 10, 59, 83]. In contrast, *Antp‐*QBP1 co‐treatment reversed these alterations, restoring proteostatic and metabolic balance while reducing inflammatory signalling. Notably, *Antp‐*QBP1 alone did not affect basal gene expression, indicating that its activity is specifically triggered under amyloidogenic stress. The reciprocal regulation of *Hspa5* and *Slc2a2* suggests that relief of ER stress contributes to the restoration of β‐cell metabolic competence [84, 87, 88]. This differential response may reflect the mild baseline stress imposed by high‐glucose culture conditions, under which control cells already exhibit altered proteostatic and metabolic states, potentially explaining why *Antp‐*QBP1 does not simply restore gene expression to control levels but further reduces *Hspa5* and increases *Slc2a2* expression [59].

Together, these findings support a mechanism in which inhibition of early aggregation mitigates downstream cellular stress responses, including ER stress and inflammation [84, 87, 89, 122, 123], and these transcriptional changes should be interpreted as complementary indicators of cellular stress rather than a comprehensive mechanistic framework. Further investigation is required to fully define the underlying regulatory mechanisms.

To complement these findings, we evaluated QBP1 in a β‐cell model stably overexpressing hIAPP (INS‐1E‐hIAPP), which reproduces chronic amyloidogenic stress [57]. Although intracellular overexpression systems present limitations, including variability in expression levels and limited control over aggregation state [10, 57], they provide a complementary context to assess sustained intracellular toxicity. Consistent with the exogenous model, *Antp‐*QBP1 attenuated hIAPP‐associated cellular alterations and improved cell viability, supporting its protective potential under chronic intracellular stress [90].

It is important to note that, while glucose‐stimulated insulin secretion (GSIS) assays are widely used to assess β‐cell function, immortalized β‐cell lines such as INS‐1E are not optimal systems for this purpose, as they exhibit reduced glucose responsiveness and altered secretion dynamics compared with primary islets [10, 57, 124]. Moreover, hIAPP overexpression itself induces a baseline impairment in GSIS, limiting its utility as a discriminative readout to evaluate protective effects [57, 125]. In this context, a conclusive functional validation of QBP1 will require more physiologically relevant systems, such as primary islets or in vivo models. Consistent with this, transgenic models expressing hIAPP develop progressive amyloid deposition and β‐cell dysfunction resembling human T2D [82, 126], supporting future evaluation of QBP1 in these systems.

A key finding from our data is that QBP1 offers protection against hIAPP‐induced toxicity both intracellularly and extracellularly [14], likely by limiting early oligomer formation. Although these compartments differ mechanistically—intracellular aggregation primarily perturbs secretory and metabolic pathways [57, 90, 127], whereas extracellular species interact with membranes and trigger ER stress [10, 59]—both ultimately converge on β‐cell dysfunction. The efficacy of QBP1 in both contexts underscores its mechanistic versatility and supports its potential as a broad‐spectrum modulator of hIAPP aggregation. Future studies dissecting these context‐specific pathways will be essential to refine QBP1‐based therapeutic strategies and tailor them to different stages of islet amyloid pathology.

Finally, MD simulations provided structural insight on the interaction between QBP1 and hIAPP. Consistent with its broad anti‐amyloid specificity [41], QBP1 preferentially associates with amyloidogenic segments of hIAPP (residues 8–20, 20–29, and 30–37) [16, 17], through predominantly nonpolar interactions, including van der Waals and π–H contacts mediated by aromatic residues such as Trp and Phe [41, 45]. This binding mode is compatible with interference in β‐sheet stacking and early nucleation events, in agreement with the experimentally observed delay in amyloid formation [11, 13, 107]. Although the estimated binding free energy (ΔG_bind_ = –5.70 kcal·mol^−^^1^) is relatively modest compared with high‐affinity small‐molecule ligands [96, 97], values in this range are commonly reported for short peptides interacting with transient and heterogeneous aggregation interfaces.

The importance of the aromatic WW motif was further supported by mutational analysis. Substitution or scrambling of Trp residues markedly reduced binding and inhibitory activity [41, 45, 128], while the W→A mutant (WKAAPGIF) abolished effective binding, showing loss of contacts with the 20–29 amyloid core and an unfavorable binding energy. In contrast, SC variants formed compact assemblies reminiscent of peptide condensates [76, 78], which may sequester hIAPP without fully preventing its self‐association, consistent with the accumulation of oligomeric intermediates at their surface [129]. Together, these observations indicate that sequence specificity (not just the amino acid composition), aromatic content, and structural organization are critical for QBP1 function.

Compared with other peptide‐based hIAPP inhibitors, QBP1 exhibited robust anti‐amyloid activity at substantially lower concentrations [102, 103]. For example, FLPNF required approximately 200 µM to reduce hIAPP‐induced cytotoxicity [77], whereas similar protection was achieved with 10 µM QBP1, and D‐ANFLVH required a ten‐fold molar excess relative to IAPP to prevent toxicity in Rin1056A β‐cells [24, 25], whereas equimolar QBP1 was sufficient. These findings highlight the high potency of QBP1, which should be advantageous for clinical translation by reducing dose requirements and potential off‐target effects.

QBP1 has also shown activity in unrelated aggregation‐prone systems, such as polyQ‐driven toxicity models, where *Antp*‐QBP1 reduced inclusion‐body formation in COS‐7 cells at low micromolar concentrations [38]. Originally designed to target expanded polyQ stretches, QBP1 achieved near‐complete inhibition of thio‐Q62 aggregation at a 3:1 molar ratio (thio‐Q62:QBP1) [33], although higher doses were required for more aggregation‐prone targets such thio‐Q81 (1:10, thio‐Q81: QBP1) [32], likely reflecting increased aggregation propensity and binding site availability. Consistent with the rationale outlined above, future studies should evaluate PTD‐QBP1 in mammalian T2D models to assess its stability, pharmacokinetics, and in vivo efficacy. In addition, given the sequence similarity and reported cross‐seeding between hIAPP and Aβ [130, 131, 132], it will be of interest to determine whether QBP1 can also modulate Aβ–hIAPP interactions.

## 5 Conclusions

Our results stablish QBP1 as a potent and versatile conformational modulator of human IAPP amyloidogenesis, demonstrating comparable or superior efficacy to that shown by other target amyloids and by other inhibitors. By delaying the onset of amyloid formation, QBP1 not only limits the accumulation of toxic species but also alleviates the harmful impact of human IAPP amyloid formation on pancreatic cells. Consequently, QBP1 emerges as a promising therapeutic candidate for the prevention and management of type 2 diabetes, offering new perspectives in the development of treatments targeting this and other amyloid‐related disorders. Furthermore, the insights gained into the cellular and molecular mechanisms of QBP1 pave the way for future preclinical developments. In particular, the molecular mechanistic data provide a blueprint for structure‐based rational design to further optimize QBP1, if needed.

## Conflicts of Interest

M.M.T.‐O. and M.C.‐V. are co‐inventors of a PCT patent recently filed to protect the application of QBP1 for T2D (PCT/EP2025/064030).

## Supporting information




**Supporting File**: advs75053‐sup‐0001‐SuppMat.docx.

## Data Availability

The data that support the findings of this study are available in the supplementary material of this article.
