# Supplementary material for: QBP1 Peptide as a Potential Anti‐Amyloidogenic Therapy for Type 2 Diabetes: An In Vitro Study
Source: Adv Sci (Weinh). 2026 Apr 22;13(37):e08344. doi: 10.1002/advs.202508344 (PMC13326014; doi:10.1002/advs.202508344)
Supplement: Supplementary file 1 — Supporting File: advs75053‐sup‐0001‐SuppMat.docx. [file ADVS-13-e08344-s001.docx]

**Supporting Information**:

**QBP1 peptide as a potential anti-amyloidogenic therapy for type 2 diabetes: an *in vitro* study**

María M. Tejero-Ojeda^1, 2^, Ada Bernaus Vives^1^, Michał Wojciechowski^3^, Dinh Quoc Huy Pham^3^, Mateusz Chwastyk^3^, Mario Vallejo^4, 5^, Anna Novials^4, 6^, Douglas V. Laurents^7^ and Mariano Carrión-Vázquez^1 *^

^1^ Instituto Cajal, Consejo Superior de Investigaciones Científicas (CSIC), Madrid, Spain

^2^ PhD Program in Neuroscience, Universidad Autónoma de Madrid/Instituto Cajal, Madrid, Spain 28029

^3^ Institute of Physics, Polish Academy of Sciences, Warsaw, Poland

^4^ Centro de Investigación Biomédica en Red de Diabetes y Enfermedades Metabólicas Asociadas CIBERDEM, Madrid, Spain

^5^ Instituto de Investigaciones Biomédicas Sols-Morreale, CSIC/Universidad Autónoma de Madrid, Madrid, Spain

^6^ Institut d'Investigacions Biomèdiques August Pi i Sunyer (IDIBAPS), Barcelona, Spain

^7^ Instituto de Química Física Blas Cabrera, CSIC, Madrid, Spain

^*^ Corresponding author: [mcarrion@cajal.csic.es](mailto:mcarrion@cajal.csic.es)

**Table S1:**

| **Gene** | **Species** | **Forward (5′→3′)** | **Reverse (5′→3′)** |
| --- | --- | --- | --- |
| ***Hspa5*** | *Rattus norvegicus* | CATCTGGGTGGGGAAGACTT | AATCCTCGCTTGATGCTGAG |
| ***Il1b*** | *Rattus norvegicus* | ACAGCAATGGTCGGGACATA | CTGAGAGACCTGACTTGGCA |
| ***Slc2a2*** | *Rattus norvegicus* | GATGCTACACTTGGCTCAGC | CACAAGCAGCACAGAGACAG |
| ***Ccl2*** | *Rattus norvegicus* | TTCCTCCACCACTATGCAGG | AGCCGACTCATTGGGATCAT |
| ***Rn18s*** | *Rattus norvegicus* | CGTTCTTAGTTGGTGGAGCG | CCGGACATCTAAGGGCATCA |

***Table S1. Oligonucleotide primers used for gene expression analyses by real-time quantitative PCR (RQ-PCR).*** Forward (Fw) and reverse (Rv) primer sequences used for RQ-PCR analysis of *Hspa5, Il1b*, *Slc2a2, Ccl2*, and *Rn18s* (reference gene) in *Rattus norvegicus*. The sequences were designed and validated to ensure optimal specificity and amplification efficiency for quantitative gene expression studies.

**Fig. S1:**


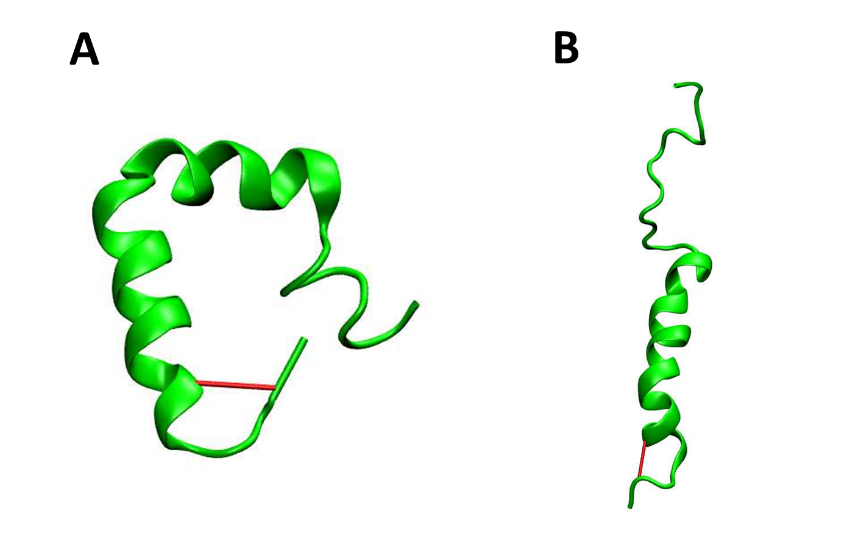


***Figure S1. Three-dimensional structures of amylin conformers used as starting models for docking and molecular dynamics simulations.*** The Cys2–Cys7 disulfide bonds are highlighted in red. **(A)** Human islet amyloid polypeptide (hIAPP) NMR solution structure in SDS micelles at pH 7.3 (**PDB ID: 2L86**; (Nanga et al. 2011)). This conformer was used as the receptor to model binding of QBP1 (WKWWPGIF), the Trp-depleted variant WKAAPGIF, and the scrambled variants SC-M8 and SC-M11. The native Cys2–Cys7 disulfide bond is preserved. **(B)** Rodent islet amyloid polypeptide (rIAPP), a non-amyloidogenic ortholog of hIAPP (**PDB ID: 2KJ7**; (Nanga et al. 2009)), was included as a negative-control background under the same docking/MD protocol. rIAPP contains key substitutions (H18R, F23L, A25P, S28P, S29P, and I33V) that disrupt β-sheet formation (Ridgway et al. 2020).

**Fig. S2:**

***Figure S2. Expanded ThT plateau quantification including full control set****.* ThT fluorescence was measured at 24 h for QBP1:hIAPP samples and at 40 h for SC:hIAPP samples to capture their respective plateau phases. Both QBP1:hIAPP molar ratios (1:4, 1:5) significantly reduced ThT fluorescence compared with hIAPP alone (****p < 0.001*), with the 1:5 ratio showing the strongest inhibition. SC-M8 and SC-M11 (1:5) also produced a significant reduction (****p < 0.001*), although their plateau fluorescence remained higher than that observed for QBP1, consistent with a more modest inhibitory activity. Control conditions (buffer, QBP1, SC-M8, SC-M11 alone) did not produce detectable ThT fluorescence in the absence of amyloidogenic hIAPP. Consistent with its non-amyloidogenic nature, rIAPP showed only near-baseline ThT fluorescence, and co-incubation with QBP1 (1:5) did not alter this profile. *Bars represent mean ± SEM from at least three independent experiments. Statistical analyses were performed using one-way ANOVA followed by Dunnett’s post hoc test vs. hIAPP, whereas the comparison between rIAPP and rIAPP+QBP1 conditions was assessed separately using an unpaired two-tailed Student’s t-test (ns).*

**Fig. S3:**


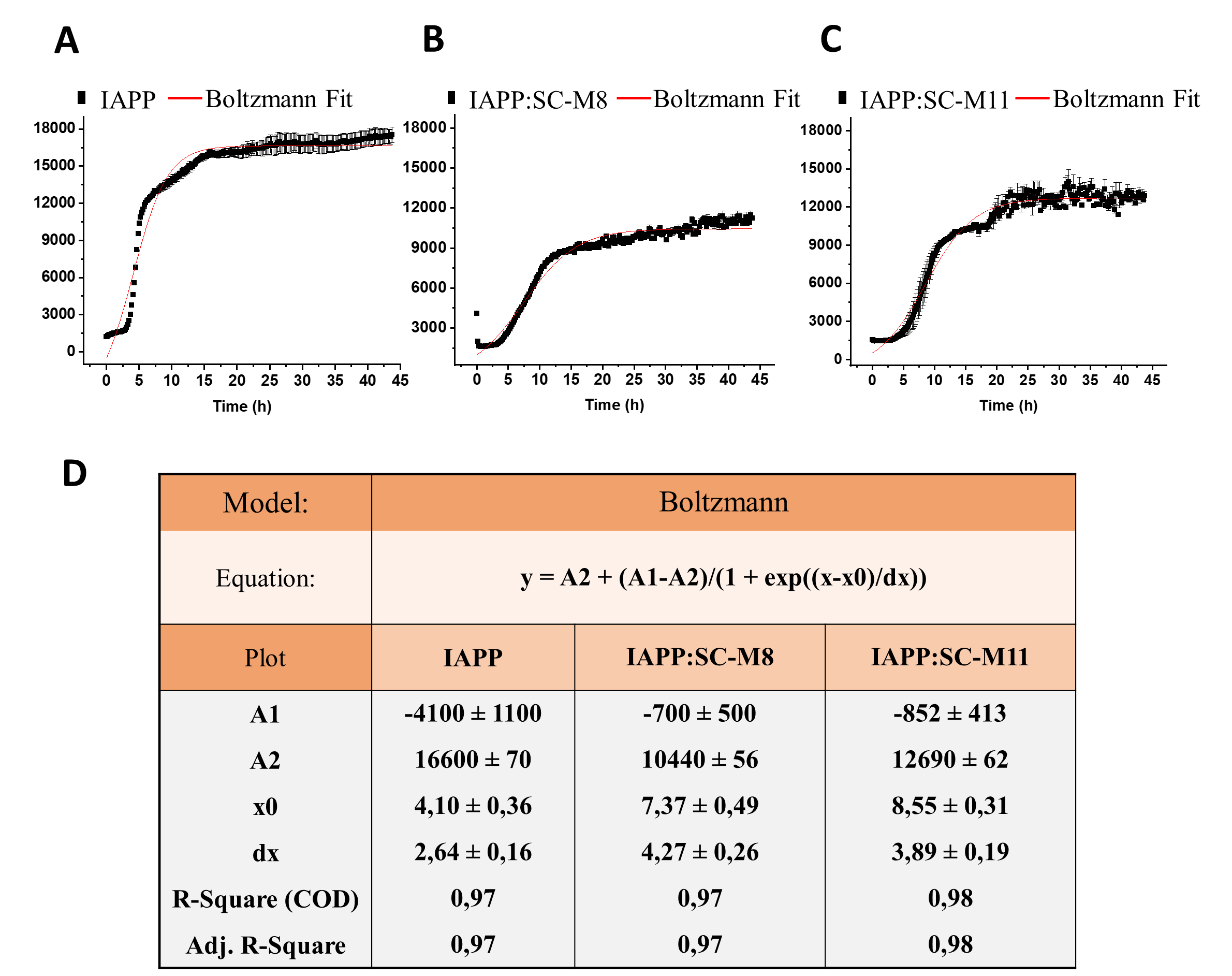


***Figure S3. Kinetic modelling of hIAPP aggregation in the presence and absence of scrambled variants (SC-M8 and SC-M11).* (A–C)** ThT fluorescence aggregation curves fitted to a non-linear Boltzmann model using OriginPro for (A) hIAPP alone, (B) hIAPP:SC-M8 (1:5 molar ratio), and (C) hIAPP:SC-M11 (1:5 molar ratio). All conditions exhibited excellent fits (R² ≥ 0.97), enabling reliable extraction of kinetic parameters. **(D)** Summary of Boltzmann fitting parameters, including baseline fluorescence (A1), maximum fluorescence intensity (A2), transition midpoint (x₀), slope (dx), and goodness-of-fit metrics (R² and adjusted R²). Compared with hIAPP alone (A2 = 16,600 ± 70 *a.u.*; x₀ = 4.10 ± 0.36 h), both SC variants reduced the maximum fluorescence intensity (SC-M8 = 10,440 ± 56 *a.u.*; SC-M11 = 12,690 ± 62 *a.u.*) and delayed the transition midpoint (SC-M8 = 7.37 ± 0.49 h; SC-M11 = 8.55 ± 0.31 h), consistent with partial inhibition of amyloid aggregation.

**Fig. S4:**

**
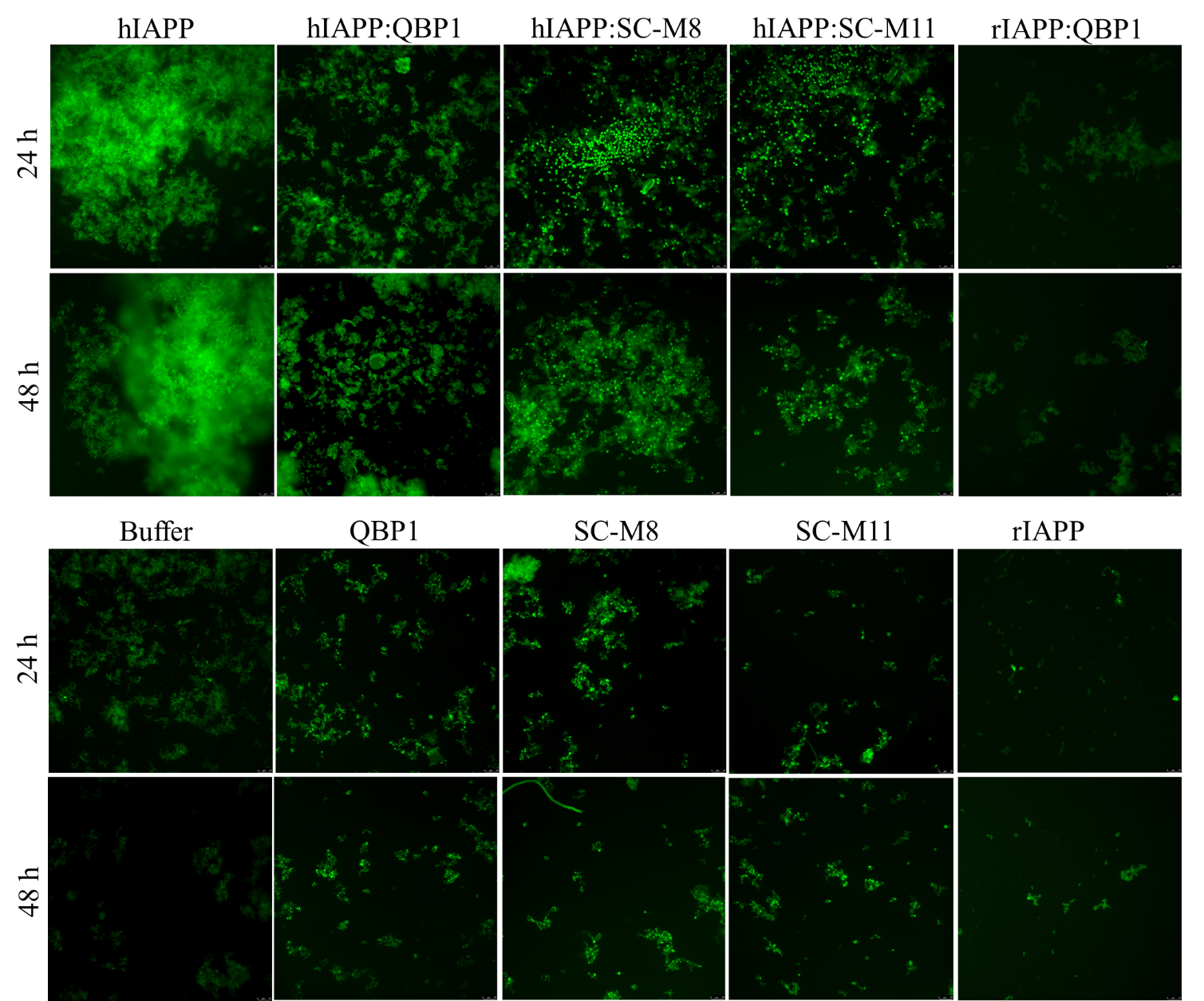
**

***Figure S4. Low-magnification fluorescence microscopy of ThT-stained samples including full control set.*** Representative fluorescence microscopy images (10×) of ThT-stained hIAPP samples incubated for 24 and 48 h in the absence or presence of inhibitory peptides (QBP1, SC-M8, and SC-M11; 1:5 molar ratio). hIAPP alone exhibited strong, time-dependent ThT fluorescence, whereas co-incubation with QBP1 markedly reduced signal intensity. SC-treated samples showed intermediate fluorescence levels, consistent with their partial inhibitory activity. The bottom panels display additional control and reference conditions, including buffer and peptides alone (QBP1, SC-M8, and SC-M11), all of which showed negligible ThT signal. rIAPP samples, either alone or co-incubated with QBP1, also exhibited minimal fluorescence, consistent with the non-amyloidogenic nature of this ortholog. This low-magnification imaging provides a broader field of view and complements the higher-resolution images shown in Fig. 1F. Images report ThT signal distribution only and do not provide structural or morphological information. Scale bars: 75 µm. Images are representative of at least three independent experiments.

**Fig S5:**

**
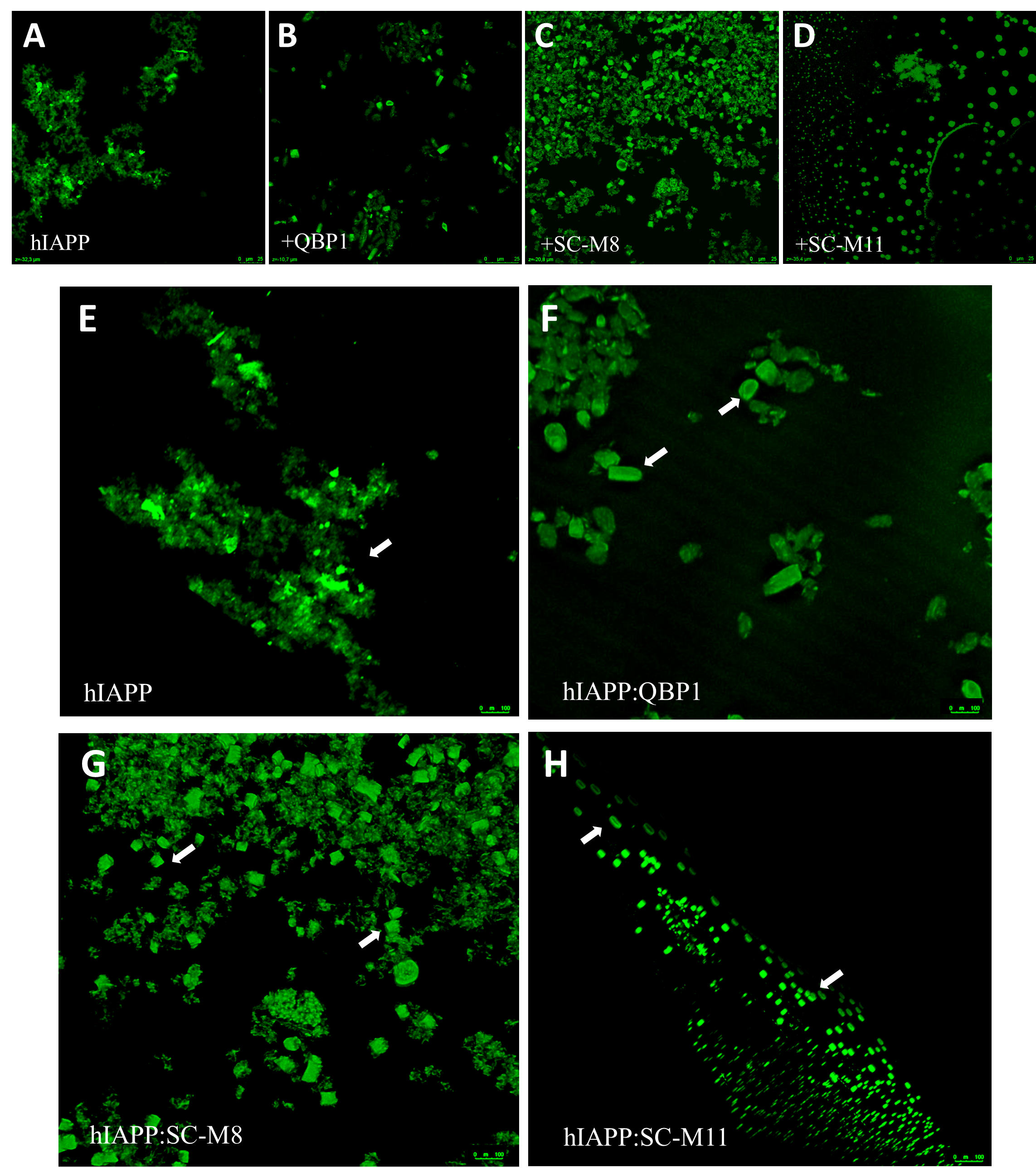
**

***Figure S5. Confocal fluorescence microscopy and three-dimensional reconstructions of ThT-stained hIAPP samples after prolonged incubation.*** hIAPP samples incubated for 7 days in the absence or presence of inhibitory peptides (QBP1, SC-M8, and SC-M11; 1:5 molar ratio) were imaged using a Leica Stellaris 8 STED system with a 40× oil-immersion objective. Panels A–D show representative 2D confocal images, and panels E–H display the corresponding three-dimensional reconstructions derived from confocal z-stacks, providing enhanced spatial visualization of ThT signal distribution. **(A, E)** hIAPP alone formed dense ThT-positive clusters. **(B, F)** Co-incubation with QBP1 markedly reduced and fragmented the fluorescence signal. **(C, G)** SC-M8 treatment produced compact, sharply defined ThT-positive assemblies. **(D, H)** SC-M11 treatment generated predominantly spherical or ring-like ThT-positive structures, with apparent central voids in the 3D reconstructions. White arrows highlight representative structures. Scale bars: 25 µm. Images are representative of at least three independent experiments.

**Fig. S6:**

**
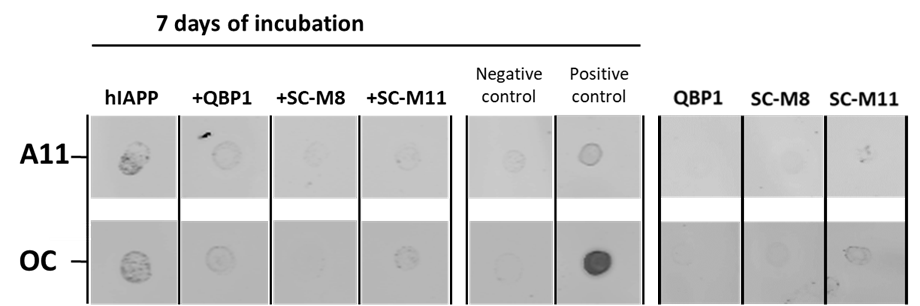
**

***Figure S6. Immunodot-blot analysis of hIAPP aggregation after prolonged incubation in the presence of QBP1 and scrambled variants.*** Samples containing hIAPP (70 μM) alone or co-incubated with QBP1, SC-M8, or SC-M11 (1:5 molar ratio) were probed with A11 (oligomer-specific) and OC (fibril-specific) antibodies after 7 days of incubation. hIAPP alone showed clear A11 and OC immunoreactivity, whereas all peptide-treated samples displayed markedly reduced signal intensity. Control reactions containing QBP1, SC-M8, or SC-M11 alone (*rightmost panels*) showed negligible signal. BSA and Aβ42 served as negative and positive controls, respectively. Representative results from at least three independent experiments.

**Fig. S7:**

***
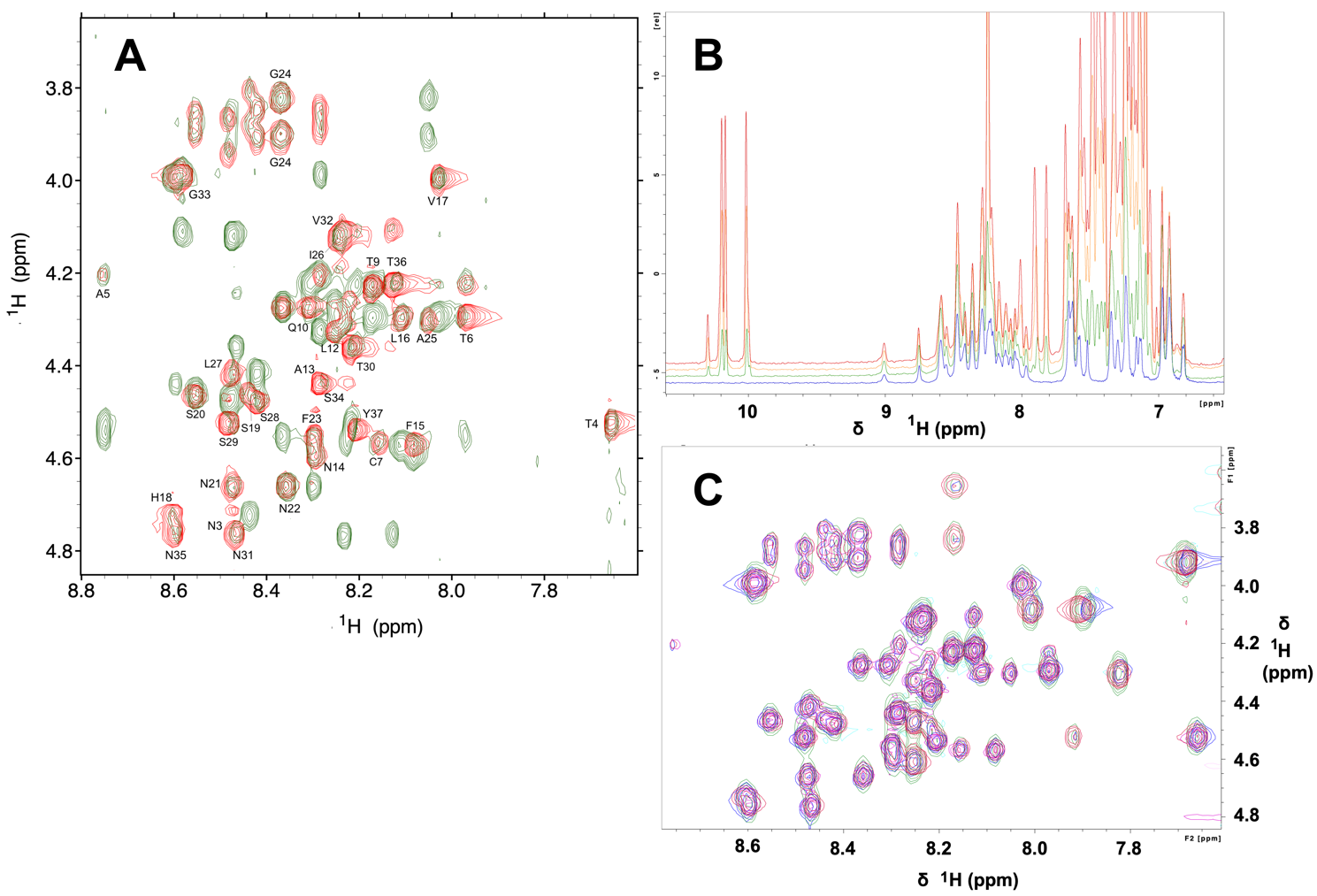
***

***Figure S7. NMR spectra of hIAPP in the absence or presence of QBP1.* (A)** Assigned ^1^HN-^1^Hα region of the 2D TOCSY (red) and 2D NOESY (green) NMR spectra of hIAPP (100 μM) recorded at pH 5.6 and 10 °C. Under these conditions, hIAPP aggregation is very sluggish. Intraresidue ^1^HN-^1^Hα cross-peak assignments are labeled. **(B)** One-dimensional (1D) ^1^H NMR spectra of 100 μM hIAPP at pH 5.6 and 10 °C alone (blue) or in the presence of 1 equivalent (green), 3 equivalents (orange) and 5 equivalents (red) of QBP1. The signals near 10 ppm are the ^1^HN indole of the Trp residues of QBP1; hIAPP contains no Trp residues. **(C)** 2D ^1^HN-^1^Hα region NMR spectra of 100 μM hIAPP at pH 5.6 and 10 °C alone (purple) or in the presence of 1 equivalent (blue), 3 equivalents (green) and 5 equivalents (red) of QBP1.

**Table S2:**


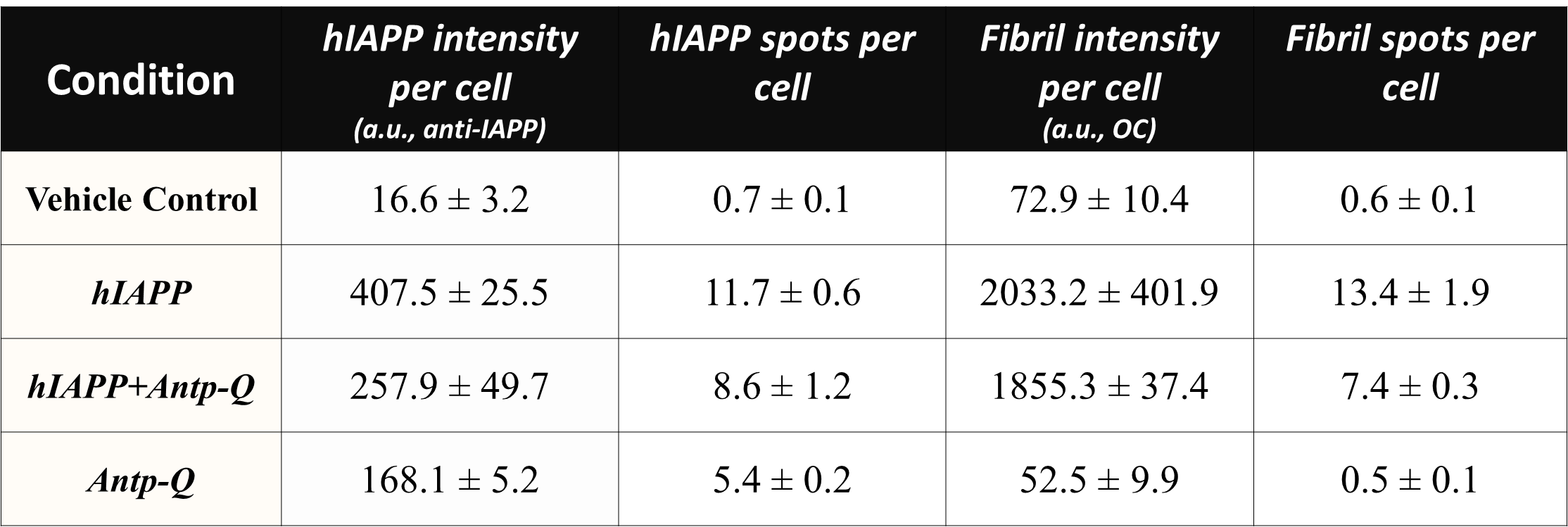


***Table 2. Intracellular hIAPP and fibrillar aggregate quantification by immunofluorescence.*** Data represent (i) *mean normalized fluorescence intensity per cell*, reflecting intracellular hIAPP accumulation (anti-hIAPP) or OC immunoreactivity, and (ii) the number of fluorescence spots per cell, indicating subcellular distribution patterns. Values are expressed as mean ± SEM and were quantified from confocal images processed with ImageJ/Fiji (NIH, USA). A custom Fiji macro based on the *Find Maxima* algorithm was used to automatically detect local intensity peaks across fluorescence channels.

**Fig. S8:**

**
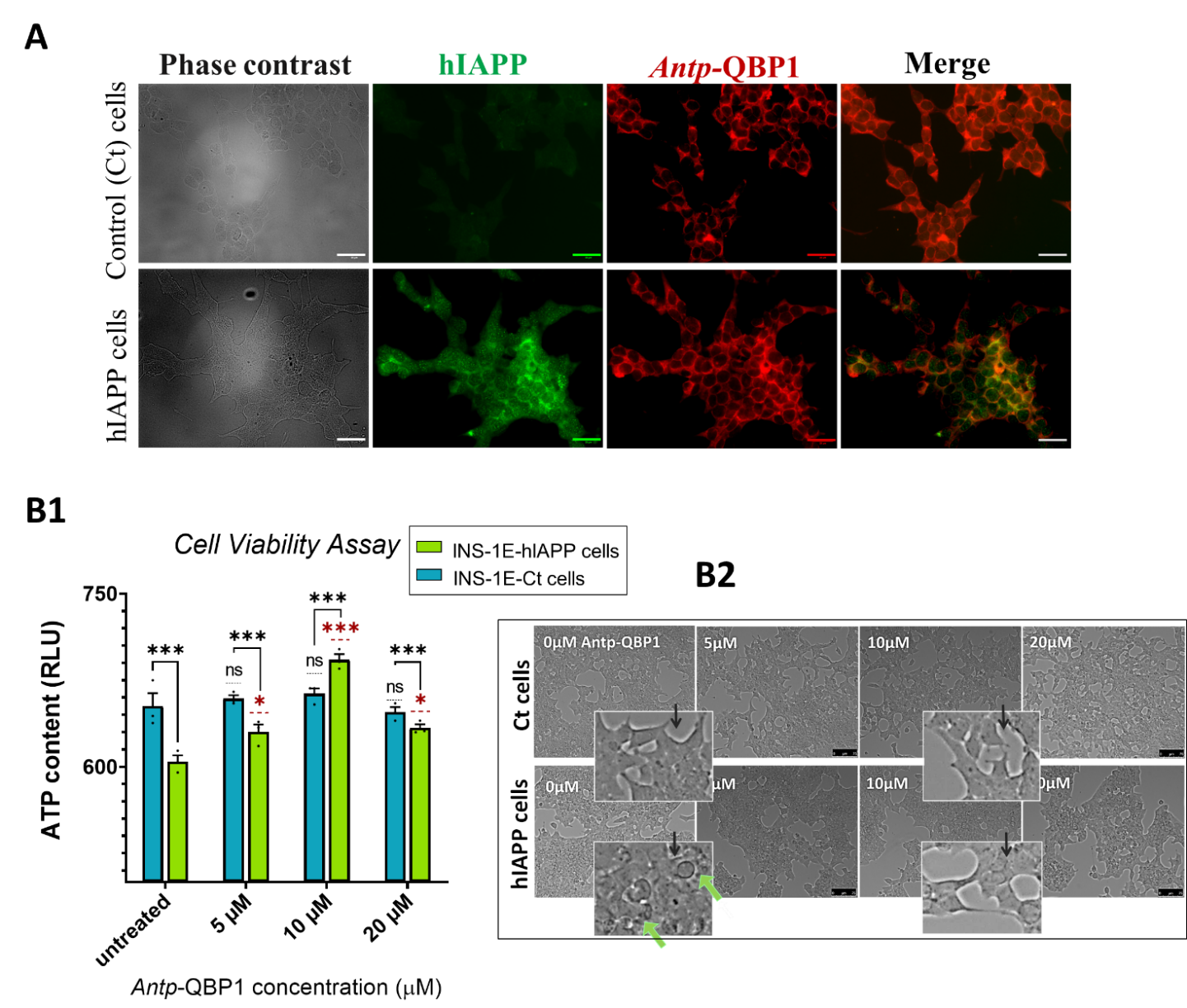
**

***Figure S8. Intracellular delivery and protective effects of Antp-QBP1 in INS-1E β-cells stably expressing hIAPP.* (A)** Representative immunocytochemistry (ICC) images of INS-1E cells stably expressing human IAPP (INS-1E-hIAPP) and non-transduced control cells (INS-1E-Ct) treated with Antp-QBP1 (10 µM, 48 h). hIAPP is shown in green (anti-IAPP, FITC channel) and *Antp-*QBP1 in red (anti-biotin, Red-647 channel); merged images are also shown. Phase-contrast images are included to visualize cell morphology. Images were acquired using a Leica DMI 6000 fluorescence microscope with a 40× oil objective. Robust hIAPP expression is detected in INS-1E-hIAPP cells together with efficient intracellular *Antp*-QBP1 signal. **(B1)** Intracellular ATP levels in INS-1E-hIAPP and INS-1E-Ct cells treated with *Antp-*QBP1 (5–20 µM, 48 h), measured using the CellTiter-Glo® assay. INS-1E-hIAPP cells showed significantly reduced ATP levels compared with Ct cells (****p < 0.001*). *Antp-*QBP1 treatment significantly restored ATP levels in INS-1E-hIAPP cells (**p < 0.05*; ****p < 0.001* *vs*. untreated; red asterisks), with the strongest effect observed at 10 µM. No significant changes were detected in Ct cells (*ns*). Direct comparisons between INS-1E-hIAPP and Ct cells under identical *Antp-*QBP1 concentrations remained significant across all conditions. **(B2)** Representative phase-contrast images of INS-1E-hIAPP and Ct cells treated with increasing concentrations of *Antp-*QBP1 (0–20 µM, 48 h). Untreated INS-1E-hIAPP cells show morphological alterations, including cell rounding and partial detachment (green arrows), whereas *Antp-*QBP1 treatment preserves cell morphology. Insets show selected areas at higher magnification. Scale bars: 75 µm. *Data in (B1) represent mean ± SEM from at least three independent experiments. Statistical analysis was performed using one-way ANOVA followed by Dunnett’s post hoc test for comparisons vs*. *untreated INS-1E-hIAPP cells and Tukey’s multiple comparisons test for pairwise comparisons between INS-1E-hIAPP and Ct conditions. Statistical significance is indicated as *p < 0.05, **p < 0.01, ***p < 0.001; ns, not significant.*

**Fig. S9:**


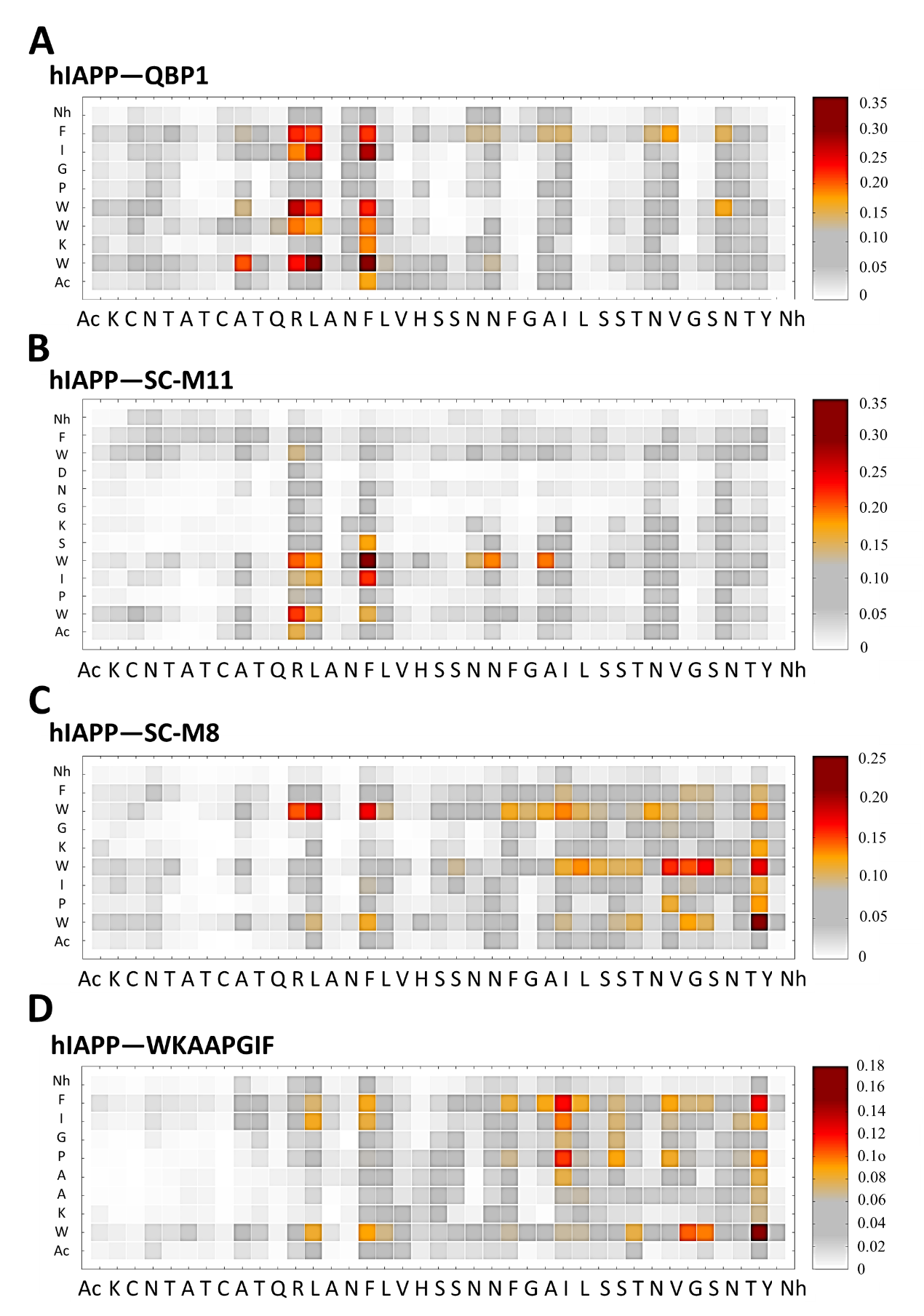


***Figure S9. Averaged residue–residue contact maps of hIAPP–peptide complexes.*** Averaged contact probability maps derived from MD trajectories, showing residue–residue interaction patterns for **(A)** hIAPP–QBP1, **(B)** hIAPP–SC-M11, **(C)** hIAPP–SC-M8, and **(D)** the Trp-depleted variant hIAPP–WKAAPGIF. The x-axis corresponds to hIAPP residues, while the y-axis represents residues of the interacting peptide. ACE and NHE denote N-terminal acetylation and C-terminal amidation capping groups, respectively. The color scale indicates the probability of contact formation during the simulation (red = high probability; grey = low probability). These maps highlight key binding regions and differences in interaction patterns, reinforcing the consistency of the binding sites identified in the simulations.

**Fig. S10:**


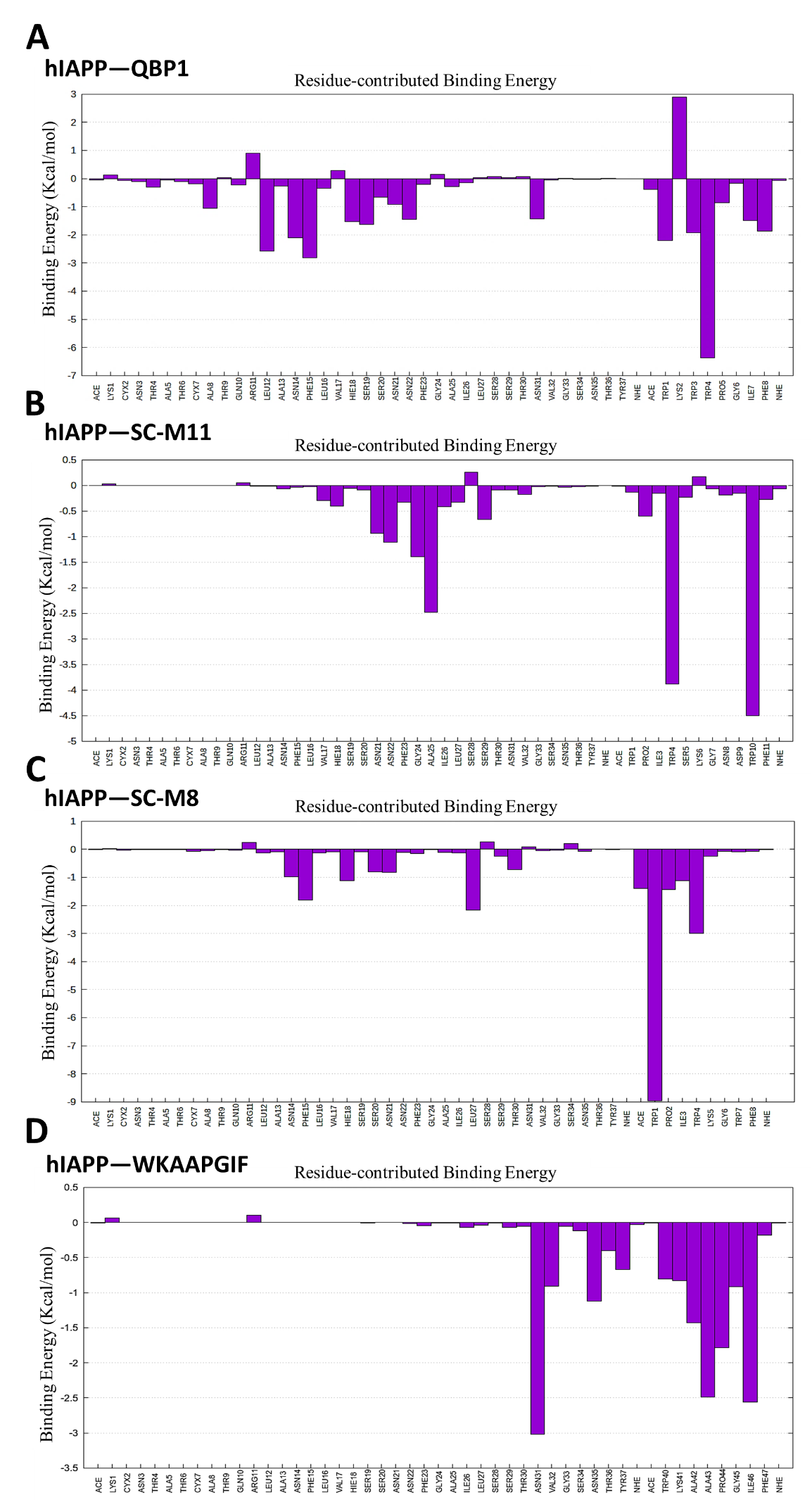


***Figure S10.*** ***Per-residue binding free energy contributions in hIAPP–peptide complexes.*** Per-residue MM/PBSA binding free energy decomposition for **(A)** hIAPP–QBP1, **(B)** hIAPP–SC-M11, **(C)** hIAPP–SC-M8, and **(D)** the Trp-depleted variant hIAPP–WKAAPGIF. Bar plots represent the contribution of individual residues to the total binding free energy, with hIAPP residues shown on the left portion of the x-axis, followed by residues of the interacting peptide. ACE and NHE denote N-terminal acetylation and C-terminal amidation capping groups, respectively. Negative values indicate favorable (stabilizing) contributions, whereas positive values indicate unfavorable contributions (kcal·mol⁻¹). Stabilizing contributions are predominantly associated with aromatic and hydrophobic residues in QBP1, whereas SC variants and the WKAAPGIF mutant display reduced and more localized energetic contributions, consistent with their weaker overall binding.

**Fig. S11:**


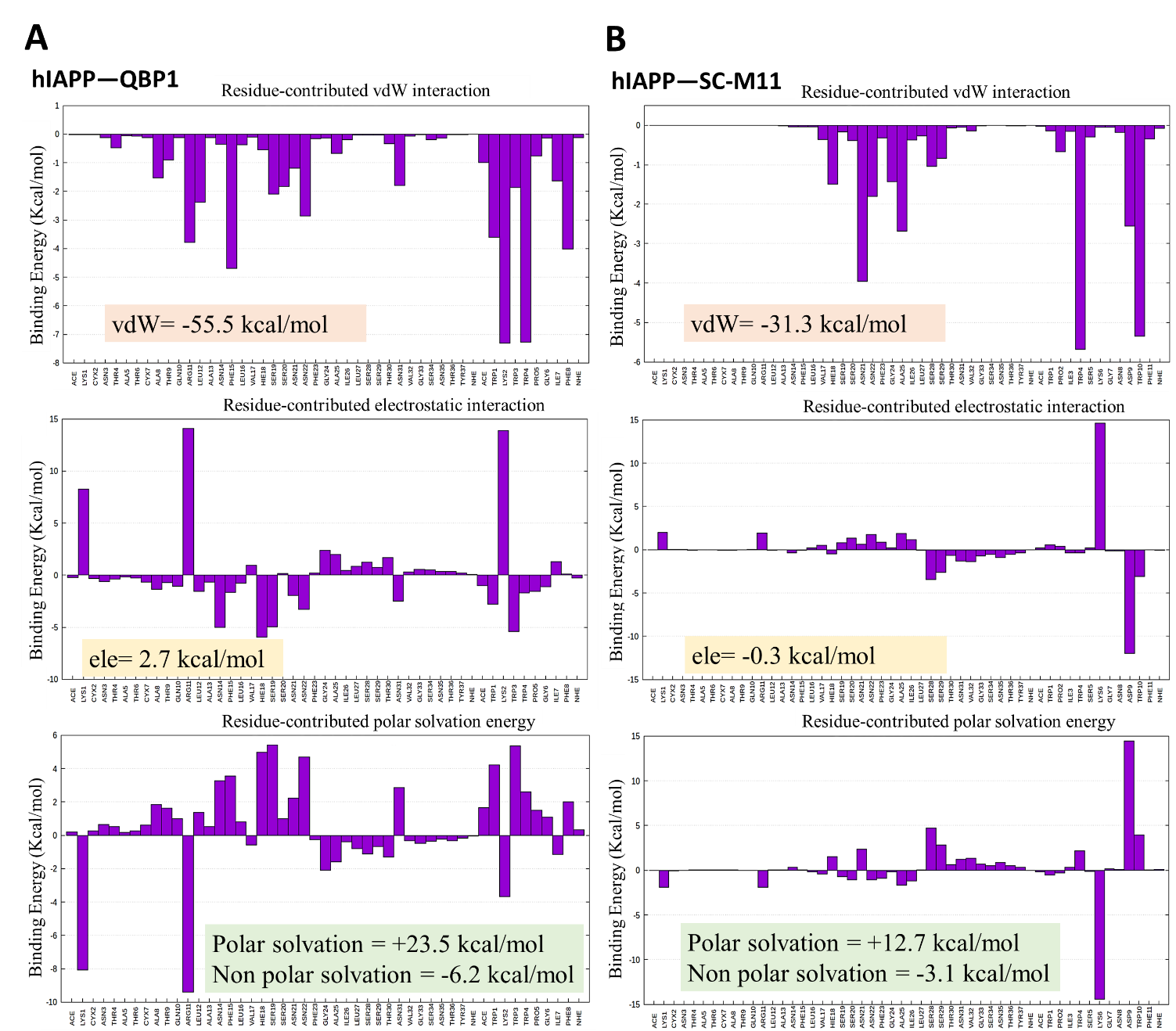


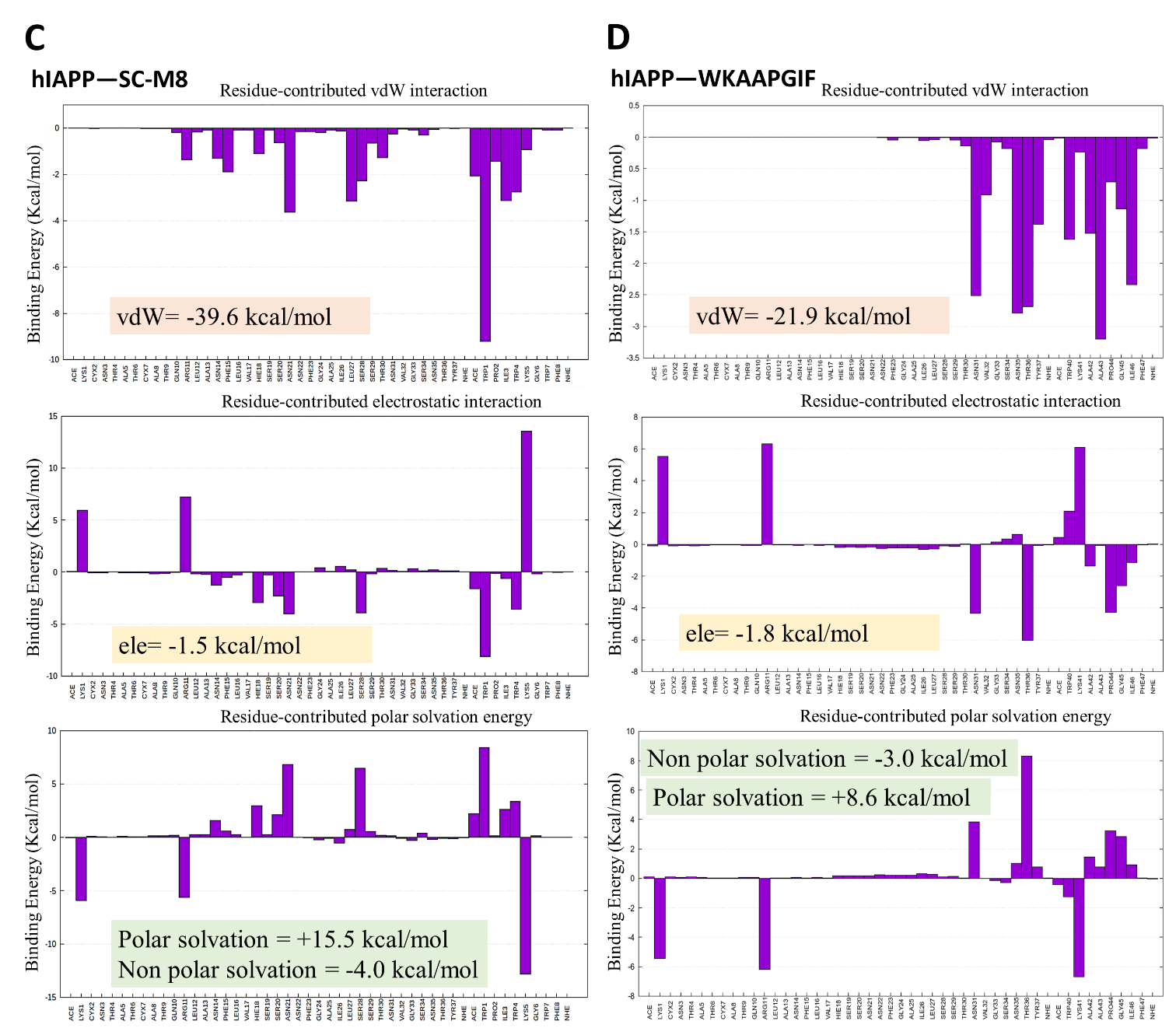


***Figure S11. Per-residue binding-energy decomposition of hIAPP–peptide complexes*.** Bar plots showing the per-residue contribution to the binding free energy of van der Waals (vdW), electrostatic (ele), and polar solvation terms for the following complexes: **(A)** hIAPP–QBP1, **(B)** hIAPP–SC-M11, **(C)** hIAPP–SC-M8, and **(D)** hIAPP–WKAAPGIF. Residues at the N-terminus correspond to hIAPP, whereas C-terminal residues correspond to the interacting peptide (QBP1, SC-M11, SC-M8, or WKAAPGIF). ACE and NHE denote N-terminal acetylation and C-terminal amidation capping groups, respectively. Negative values indicate favorable (stabilizing) contributions, while positive values indicate unfavorable contributions (kcal·mol⁻¹). Among the variants analyzed, QBP1 exhibits the strongest and most favorable vdW contributions, largely driven by hydrophobic and aromatic residues (notably Trp and Phe), consistent with its enhanced binding stability relative to SC variants and the WKAAPGIF mutant.

**Fig. S12:**


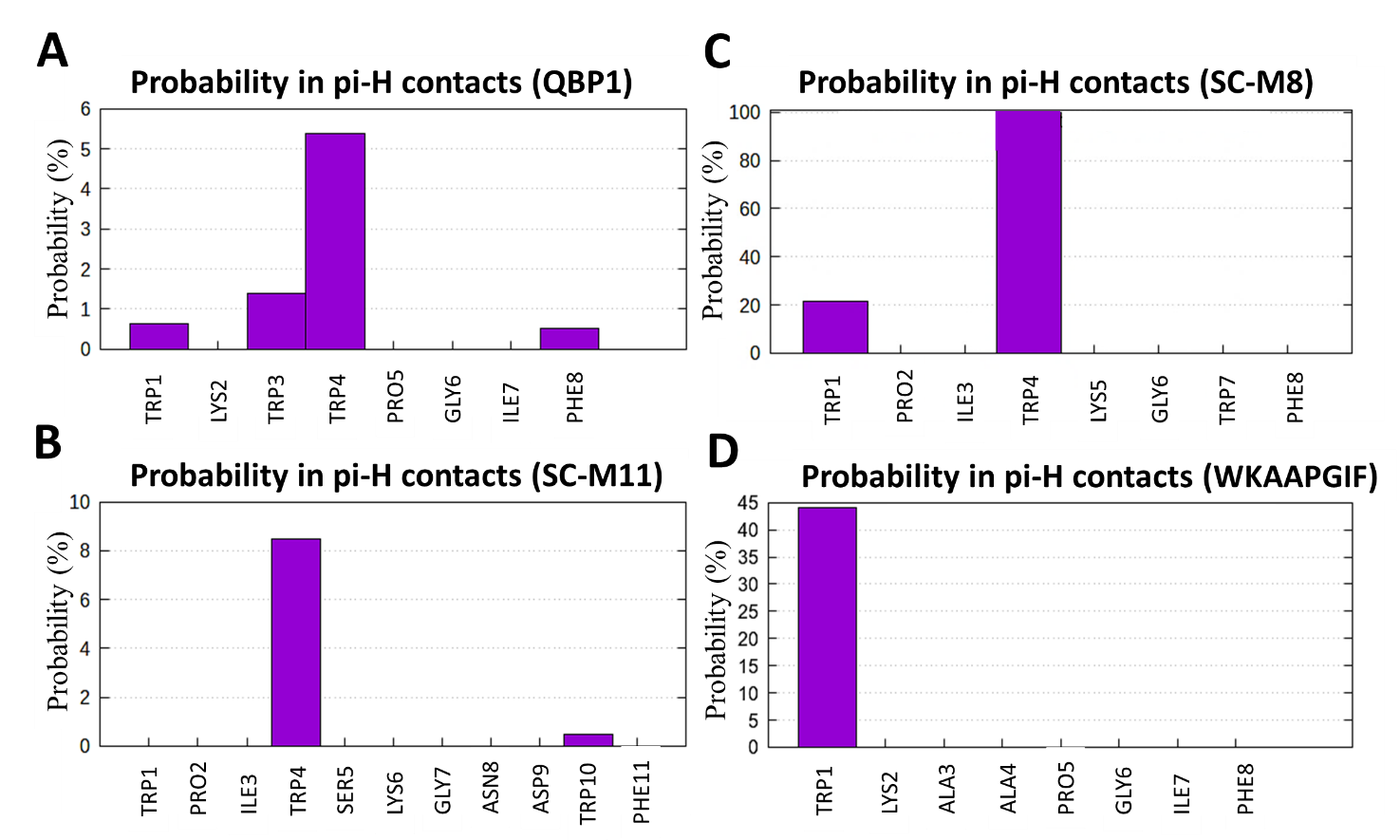


***Figure S12. Probability of π–H interaction formation in hIAPP–peptide complexes*.** The Probability of π–H bond formation calculated from MD trajectories for **(A)** hIAPP–QBP1, **(B)** hIAPP–SC-M11, **(C)** hIAPP–SC-M8, and **(D)** the Trp-depleted variant hIAPP–WKAAPGIF. A π–H interaction was defined by a distance < 4 Å between the hydrogen donor and the aromatic ring center of mass, and an angle < 60° between the ring normal and the hydrogen–donor vector. Bar plots report the probability (%) of π–H interaction events for individual residue pairs identified during the simulations. QBP1 exhibits the highest frequency of π–H interactions, predominantly involving aromatic residues (notably Trp and Phe), whereas SC variants display fewer and more sporadic events. In the WKAAPGIF mutant, π–H interactions are markedly diminished, consistent with the loss of aromatic side chains and reduced binding stabilization observed in the energetic and contact analyses.

**Fig. S13:**

**
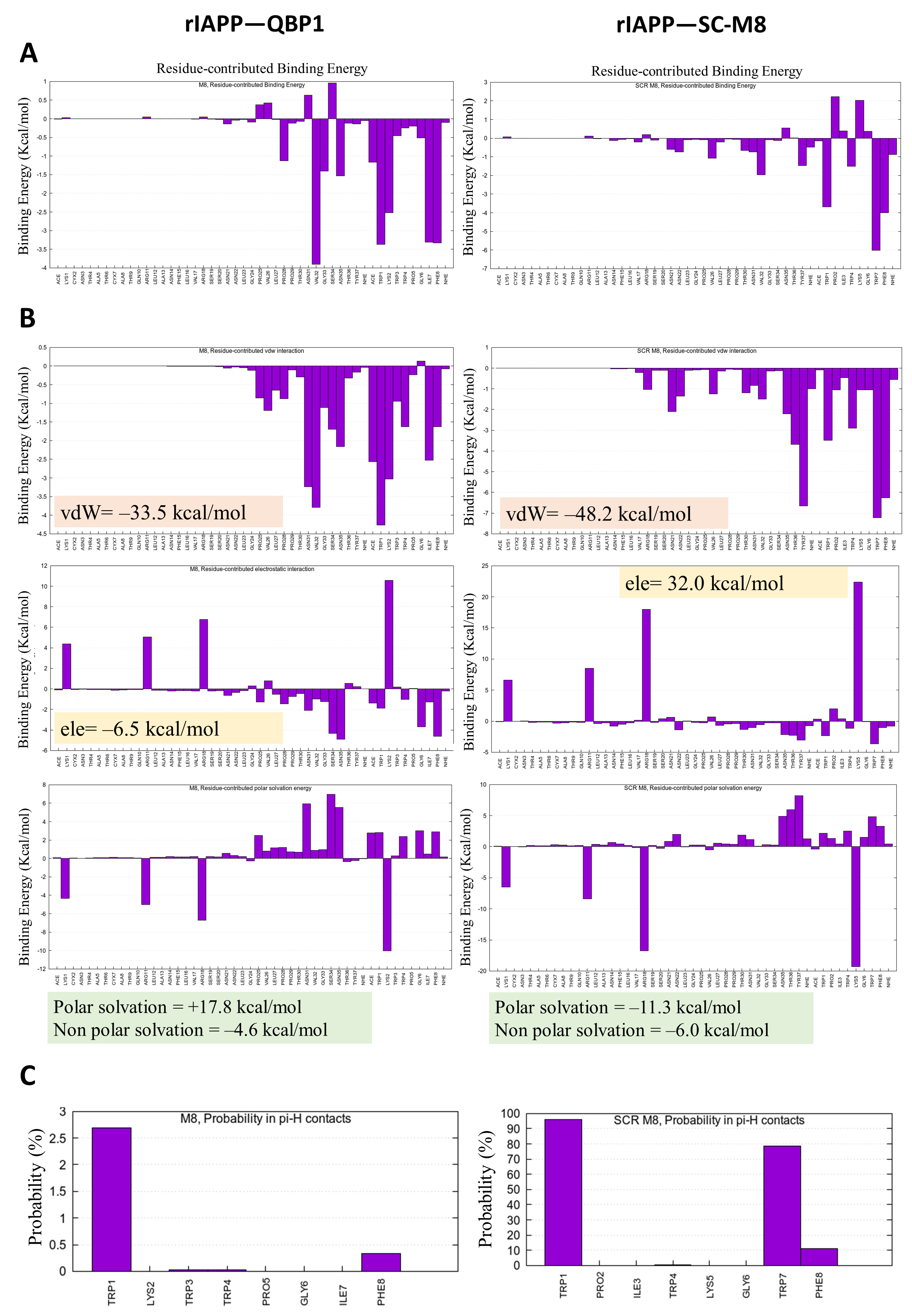
**

***Figure S13. Energetic and interaction analyses of rodent amylin complexes with QBP1 and SC-M8.*** Energetic and interaction analyses derived from molecular dynamics (MD) simulations of rIAPP–peptide complexes. ACE and NHE denote N-terminal acetylation and C-terminal amidation capping groups, respectively. Negative energy values indicate favorable (stabilizing) contributions (kcal·mol⁻¹). **(A)** Per-residue MM/PBSA binding free energy decomposition for the rIAPP–QBP1 complex (left) and the rIAPP–SC-M8 complex (right). Residues at the N-terminal portion correspond to rIAPP, followed by residues of the interacting peptide. **(B)** Per-residue decomposition of van der Waals (top), electrostatic (middle), and polar solvation (bottom) contributions to the total binding free energy for the rIAPP–QBP1 (left) and rIAPP–SC-M8 (right) complexes. **(C)** Probability of π–H interaction formation between rIAPP and QBP1 (left) or SC-M8 (right), calculated from MD trajectories. A π–H interaction was defined by a distance of < 4 Å between the hydrogen donor and the centroid of the aromatic ring, and an angle of < 60° between the ring normal and the donor–hydrogen vector.

**Figure S14:**

**
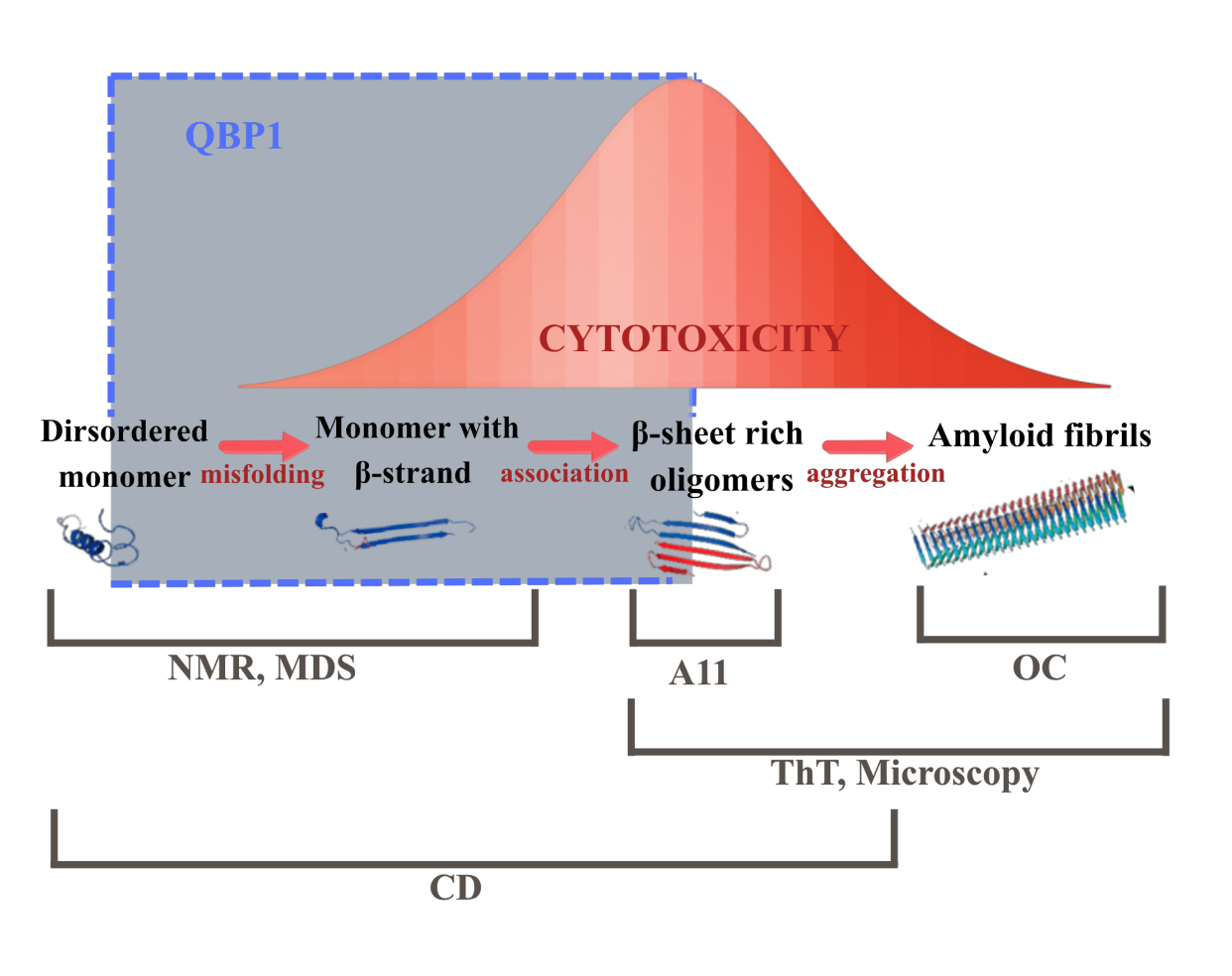
**

***Figure S14. Schematic representation of the hIAPP aggregation pathway and experimental methods used to characterize each stage.*** QBP1 preferentially acts at early misfolding and oligomerization stages, delaying β-sheet acquisition and cytotoxic aggregate build-up. The methods indicated (NMR, MD simulations, CD spectroscopy, A11/OC immunoassays, ThT fluorescence, and microscopy) correspond to the conformational states or assemblies they detect. The diagram also illustrates how QBP1 reduces cytotoxic species accumulation by stabilizing disordered and early intermediate states.

**Supplementary Methods:**

***Molecular docking and molecular dynamics simulations.*** We examined the binding between human IAPP (hIAPP; 37-residue peptide with a Cys2–Cys7 disulfide bond; sequence *KCNTATCATQRLANFLVHSSNNFGAILSSTNVGSNTY*) and the QBP1-derived inhibitors: the octapeptide QBP1 (*WKWWPGIF)* (Ramos-Martín et al. 2014), a hydrophobic-core mutant (*WKAAPGIF*; two W→A substitutions within the WKWW motif), and the scrambled variants SC-M11 (*WPIWSKGNDWF)* and SC-M8 (*WPIWKGWF*). The NMR structure of hIAPP in SDS micelles at pH 7.3 **(PDB: 2L86** (Nanga et al. 2011); **Fig. S1A)** was used as the starting model. In parallel, rodent IAPP (rIAPP; with key substitutions at Pro25/Pro28/Pro29; sequence *KCNTATCATQRLANFLVRSSNNLG****P****VL****PP****TNVGSNTY*), a non-amyloidogenic isoform **(PDB: 2KJ7** (Nanga et al. 2009); **Fig. S1B)**, was included as a negative control target. QBP1 and SC-M8 were docked and simulated against rIAPP following the same protocol. The initial QBP1 structure was taken from the experimental coordinates of Ramos-Martín *et al.* (2014), while WKAAPGIF and SC variants were obtained by all-atom MD pre-sampling.

Complexes of hIAPP (with QBP1, WKAAPGIF, SC-M11, SC-M8) or rIAPP (with QBP1, SC-M8) were generated using a docking procedure performed with the **HADDOCK server** (De Vries, Van Dijk, and Bonvin 2010). In each case, both peptides included N-terminal acetyl (ACE) and C-terminal amide (NHE) blocking groups to match the experimental setup. The docking procedure produced 10 of the most probable configurations, all of which were used as starting structures for MD simulations to generate snapshot ensembles for further analysis. To ensure reliable sampling, ten 100-ns trajectories were simulated for each configuration. During each trajectory, conformations were saved every 10 ps, resulting in a total of 100,000 structures per configuration, providing a broad sampling of accessible conformations.

The MD simulations were conducted using the **Amber20 package** (Case et al. 2020) with the AMBER 19SB force field (Tian et al. 2020) and the OPC water model (Izadi, Anandakrishnan, and Onufriev 2014), which are well-suited for simulating biological systems in explicit water. The docked structures were placed at the center of a simulation box with a buffer of at least 10 Å from the box edge. This buffer was sufficient to prevent the solute from interacting with its periodic images when a cut-off of 9 Å was applied. To mimic physiological conditions, the system was neutralized with sodium (Na^+^) and chloride (Cl^−^) ions, and the number of ions was adjusted to achieve an ionic strength of *I = 0.15 M*.

The equations of motion were integrated using the leap-frog algorithm (Leimkuhler and Matthews 2013) with a time step of 2 fs, and the SHAKE algorithm (Ryckaert, Ciccotti, and Berendsen 1977) was applied to constrain all bonds involving hydrogen atoms. The temperature was maintained at 300 K using a Langevin thermostat (Brooks et al. 1983) with a collision frequency of 2 ps^−1^. Long-range electrostatic interactions were computed using the particle mesh Ewald (**PME**) method (Darden, York, and Pedersen 1993).

Before the production run, the system underwent three preparatory steps: (1) Energy minimization of the solvated complex to eliminate steric clashes and optimize the initial structure; (2) Gradual heating of the system to 300 K under the NVT ensemble (constant number of particles, volume, and temperature), ensuring a controlled and stable temperature increase without volume fluctuations; (3) A final equilibration under the NPT ensemble (constant number of particles, pressure, and temperature) to achieve the target system density of approximately 1.0 g/cm^3^. Following these steps, conformations for analysis were collected after 500 ps of equilibration in the NVT ensemble to ensure structural stability (Roe and Cheatham 2013).

Data analysis was performed based on contact maps and binding energy calculations. Contact maps were generated independently for each collected conformation using overlap criteria (Mioduszewski et al. 2023; Chwastyk, Bernaola, and Cieplak 2015). Binding energies of the amylin complexes were calculated using the Molecular Mechanics/Poisson–Boltzmann Surface Area (**MM/PBSA**) approach (Case et al. 2016), which estimates the free energy of binding by considering both enthalpic and entropic contributions. Throughout all simulation trajectories of the amylin complexes, we monitored the binding conformations, defined as sets of contacts between the two peptides. Each specific set of contacts, which may appear or disappear temporarily, represents a distinct binding mode. Among all the binding modes observed in our simulations, we selected the most stable ones—those characterized by the most frequently occurring group of contacts. The corresponding contact maps are shown in **Figs. 8, 9** and **S9**. These binding modes provide insights into the binding capabilities of the respective peptides.

References

Brooks, Bernard R., Robert E. Bruccoleri, Barry D. Olafson, David J. States, S. Swaminathan, and Martin Karplus. 1983. “CHARMM: A Program for Macromolecular Energy, Minimization, and Dynamics Calculations.” *Journal of Computational Chemistry* 4 (2): 187–217. https://doi.org/10.1002/jcc.540040211.

Case, D.A., K. Belfon, I.Y. Ben-Shalom, S.R. Brozell, D.S. Cerutti, T.E. Cheatham, III, and D.M. York and P.A. Kollman V.W.D. Cruzeiro, T.A. Darden, R.E. Duke, G. Giambasu, M.K. Gilson, H. Gohlke, A.W. Goetz, R Harris, S. Izadi, S.A. Iz- mailov, K. Kasavajhala, A. Kovalenko, R. Krasny,. 2020. “Amber Manual 2020.” *AMBER* University. http://ambermd.org/contributors.html.

Case, D.A. DA, R.M. RM Betz, DS D.S. Cerutti, Cheatham III T.E., T.A. Darden, R.E. Duke, T.J. Giese, et al. 2016. “Amber 2016.” *University of California, San Francisco*, 191–727.

Chwastyk, Mateusz, Adolfo Poma Bernaola, and Marek Cieplak. 2015. “Statistical Radii Associated with Amino Acids to Determine the Contact Map: Fixing the Structure of a Type i Cohesin Domain in the Clostridium Thermocellum Cellulosome.” *Physical Biology* 12 (4): 46002. https://doi.org/10.1088/1478-3975/12/4/046002.

Darden, Tom, Darrin York, and Lee Pedersen. 1993. “Particle Mesh Ewald: An Nxlog(N) Method for Ewald Sums in Large Systems.” *The Journal of Chemical Physics* 98 (12): 10089–92. https://doi.org/10.1063/1.464397.

Izadi, Saeed, Ramu Anandakrishnan, and Alexey V Onufriev. 2014. “Building Water Models : A Different Approach.” *The Journal of Physical Chemistry Letters* 5 (21): 3863–71. https://doi.org/dx.doi.org/10.1021/jz501780a.

Leimkuhler, Benedict, and Charles Matthews. 2013. “Rational Construction of Stochastic Numerical Methods for Molecular Sampling.” *Applied Mathematics Research EXpress* 2013 (1): 34–56. https://doi.org/10.1093/amrx/abs010.

Mioduszewski, Łukasz, Jakub Bednarz, Mateusz Chwastyk, and Marek Cieplak. 2023. “Contact-Based Molecular Dynamics of Structured and Disordered Proteins in a Coarse-Grained Model: Fixed Contacts, Switchable Contacts and Those Described by Pseudo-Improper-Dihedral Angles.” *Computer Physics Communications* 284: 108611. https://doi.org/10.1016/j.cpc.2022.108611.

Nanga, Ravi Prakash Reddy, Jeffrey R. Brender, Subramanian Vivekanandan, and Ayyalusamy Ramamoorthy. 2011. “Structure and Membrane Orientation of IAPP in Its Natively Amidated Form at Physiological PH in a Membrane Environment.” *Biochimica et Biophysica Acta - Biomembranes* 1808 (10): 2337–42. https://doi.org/10.1016/j.bbamem.2011.06.012.

Nanga, Ravi Prakash Reddy, Jeffrey R. Brender, Jiadi Xu, Kevin Hartman, Vivekanandan Subramanian, and Ayyalusamy Ramamoorthy. 2009. “Three-Dimensional Structure and Orientation of Rat Islet Amyloid Polypeptide Protein in a Membrane Environment by Solution NMR Spectroscopy.” *Journal of the American Chemical Society* 131 (23): 8252–61. https://doi.org/10.1021/ja9010095.

Ramos-Martín, Francisco, Rubén Hervás, Mariano Carrión-Vázquez, and Douglas V. Laurents. 2014. “NMR Spectroscopy Reveals a Preferred Conformation with a Defined Hydrophobic Cluster for Polyglutamine Binding Peptide 1.” *Archives of Biochemistry and Biophysics* 558: 104–10. https://doi.org/10.1016/j.abb.2014.06.025.

Ridgway, Zachary, Charles Eldrid, Alexander Zhyvoloup, Aisha Ben-younis, Konstantinos Thalassinos, Daniel P Raleigh, Stony Brook, Gower Street, Structural Biology, and Stony Brook. 2020. “Analysis of Proline Substitutions Reveals the Plasticity and Sequence Sensitivity of Human IAPP Amyloidogenicity and Toxicity.” *Biochemistry.* 59 (6): 742–54. https://doi.org/10.1021/acs.biochem.9b01109.Analysis.

Roe, Daniel R., and Thomas E. Cheatham. 2013. “PTRAJ and CPPTRAJ: Software for Processing and Analysis of Molecular Dynamics Trajectory Data.” *Journal of Chemical Theory and Computation* 9 (7): 3084–95. https://doi.org/10.1021/ct400341p.

Ryckaert, Jean Paul, Giovanni Ciccotti, and Herman J.C. Berendsen. 1977. “Numerical Integration of the Cartesian Equations of Motion of a System with Constraints: Molecular Dynamics of n-Alkanes.” *Journal of Computational Physics* 23 (3): 327–41. https://doi.org/10.1016/0021-9991(77)90098-5.

Tian, Chuan, Koushik Kasavajhala, Kellon A.A. Belfon, Lauren Raguette, He Huang, Angela N. Migues, John Bickel, et al. 2020. “Ff19SB: Amino-Acid-Specific Protein Backbone Parameters Trained against Quantum Mechanics Energy Surfaces in Solution.” *Journal of Chemical Theory and Computation* 16 (1): 528–52. https://doi.org/10.1021/acs.jctc.9b00591.

Vries, Sjoerd J. De, Marc Van Dijk, and Alexandre M.J.J. Bonvin. 2010. “The HADDOCK Web Server for Data-Driven Biomolecular Docking.” *Nature Protocols* 5 (5): 883–97. https://doi.org/10.1038/nprot.2010.32.
